# Supplementary material for: Temporal dynamics of the multi-omic response to endurance exercise training
Source: Nature. 2024 May 1;629(8010):174–83. doi: 10.1038/s41586-023-06877-w (PMC11062907; doi:10.1038/s41586-023-06877-w)
Supplement: Supplementary file 1 — Supplementary Methods, Tables, discussion, author contributions and references. [file 41586_2023_6877_MOESM1_ESM.pdf]

nature portfolio

https://doi.org/10.1038/s41586-023-06877-w

Supplementary information

Temporal dynamics of the multi-omic response to endurance exercise training

In the format provided by the authors and unedited

Supplementary Information

EXPERIMENTAL DESIGN AND SAMPLE HANDLING 3
- Animal handling 3
- Sample distribution 6

DATA PRODUCTION AND QUANTIFICATION 8
- DNA methylation by reduced representation bisulfite sequencing 8
- Assay for transposase-accessible chromatin using sequencing 10
- RNA sequencing 11
- Liquid chromatography tandem mass spectrometry (LC-MS/MS) proteomics 12
- Non-targeted metabolomics 21
- Targeted metabolomics 28
- Metabolomics data processing and normalization 39
- Multiplexed bead-based immunoassays 40

STATISTICAL ANALYSES 42
- Outlier identification 42
- Differential analysis 43
- Training group and sex permutation analyses 44
- Meta-analysis of metabolomics results 44
- Graphical clustering of differential analysis results 45
- Comprehensive feature-to-gene map 47
- Pathway enrichment analysis of graphical clusters 47
- Molecular interaction networks 48
- Network connectivity analysis 48
- Transcription factor enrichment analysis methods 49
- Rat-to-human ortholog map 49
- Gene and PTM set enrichment analysis 49
- Mapping PTMs from rat to human proteins 50
- Correlation of training-differential features with cell type markers 50
- Immune cell type deconvolution 51
- Enrichment of LM22 immune cell types 51
- Quantification of sex differences in the training response 51
- Comparison with extant training response omics data 52
- Disease ontology enrichment analysis 53
- Metabolite enrichment by RefMet class 53

SUPPLEMENTARY DISCUSSION 54
- Multi-omic analysis of exercise training 54
  - Details for each omic analysis performed 54
  - Permutation tests demonstrate significant responses to endurance training 54

Multi-tissue response to training 55
Sexually conserved responses reveal tissue-dependent adaptations to endurance training 55
Endurance training induces a multi-tissue protein-specific increased in the abundance of heat shock proteins 56
Unique transcript and protein responses 56
TF activity and phosphosignatures 57
TF enrichment in non-skeletal muscle tissues 57
Phosphosignature analysis reveal mechanisms for exercise adaptation in liver and heart 57
Molecular hubs of exercise adaptation 58
Analysis of up-regulated features at 8 weeks of training in both sexes 58
Network connectivity analysis 59
Connection to human diseases and traits 60
Sex-specific responses to exercise 60
Transcriptional remodeling of the adrenal gland 60
Immune-related transcriptional changes in the small intestine 61
Multi-tissue changes in lipids 61
Conclusions 62
AUTHOR CONTRIBUTIONS 64
REFERENCES 70

METHODS

EXPERIMENTAL DESIGN AND SAMPLE HANDLING

Animal handling

Animals

Adult male and female Fischer 344 (F344) inbred rats were obtained from the National Institute on Aging (NIA) rodent colony in three cohorts of 20-30 rats. Rats arrived on site at least 4 weeks prior to initiation of exercise training and were handled daily to reduce stress. Upon arrival at the University of Iowa, rats were adapted to a reverse dark-light cycle with lights off at 9:00 AM and lights on at 9:00 PM so that the treadmill training occurred during the normal active part of the day for rats. Rats were housed two per cage (146.4 square inches of floor space) in ventilated racks (Thoren Maxi-Miser IVC Caging System) on Tekland 7093 Shredded Aspen bedding. Animals were fed ad libitum (not measured) with the pelleted Charles River Rat and Mouse 18% (Auto) 5L79 LabDiet (Gateway Lab Supply, St. Louis, Missouri), which has the following calorie composition: 21.196% protein, 14.774% fat (ether extract), 64.030% carbohydrates. These are the standard bedding and diet used at the NIA rodent colony. The animal housing room was monitored daily and maintained at a temperature of 68-77°F and relative humidity of 25-55%. Red lights were used during the rat's dark cycle to provide adequate lighting for the staff to perform routine housing tasks and rodent handling and training. All animal procedures were approved by the Institutional Animal Care and Use Committee at the University of Iowa.

Treadmill familiarization and training

Treadmill exercise was performed on a Panlab 5-lane rat treadmill (Harvard Instruments, Model LE8710RTS). All animal handling and exercise was performed in the active, dark phase of the rats. Upon arrival, rats were acclimated to the reverse light cycle for a minimum of 10 days. Following the initial acclimation period, rats went through a 12-day treadmill familiarization protocol to expose the rats to the treadmill and to identify potential non-compliant rats. Those rats that were unable to run on the treadmill for 5 minutes at a speed of 10 m/min and grade of 0° were classified as non-compliant and removed from the study. Rats that successfully completed the 12-day familiarization protocol were entered in the rat database and randomized into a control or training group so that mean body weight of the groups were equal. The 8-week rats were randomly assigned to control or training within sex and tertile of weight. 4-week rats were assigned to control without randomization. 1- and 2- week rats were randomly assigned to 1- or 2-week training within sex and tertile of weight.

Exercise training began at 6 months of age in male and female rats and lasted for a duration of 1, 2, 4 or 8 weeks. At 6 months of age, this rat strain has reached a growth plateau with respect to lean muscle mass. Rats were exercised on the treadmill 5 days per week using a progressive training protocol designed to exercise the rats at approximately 70% of VO_2max. The starting treadmill speed was based on VO_2max measurements obtained following familiarization and prior to training in the compliant rats. Training occurred during the dark cycle of the rat, started no earlier than 10:00 AM, and ended no later than 5:00 PM over 5 consecutive days per week. Training was initiated with the treadmill set at a grade of 5°, speeds of 13m/min for males and 16m/min for females, and a duration of 20 minutes. As outlined in Table 1, the duration of exercise was increased by one minute each day until day 31 of training (start of week 7), when a duration of 50 min was reached. The treadmill grade was increased from 5° to 10° at the start of week 3 and stayed at 10° for the remainder of the training. The treadmill speed increased at the start of week 2, 4, 5, 6 and 7. At the start of week 7, speed, grade and duration were fixed and maintained for the final 10 days of the protocol to ensure steady-state had been achieved. If a rat was unable to perform at least 4 days of training per week, it was removed from the study and euthanized. Rats assigned to the control group were placed on the treadmill (0 m/min) for 15 min/day, 5 days per week, and followed a schedule similar to the 8-week training group. The control animals were age-matched to the 8-week training group. Importantly, 6-9 month old rats are mature and stable with limited physiological differences until 12 months of age[47].

Table 1. Progressive training protocol for male and female 6-month-old rats.

**Week**	**Day**	**Speed (meters/min) Male/Female**	**Grade (degrees)**	**Duration (mins)**
1	1	13/16	5	20
	2	13/16	5	21
	3	13/16	5	22
	4	13/16	5	23
	5	13/16	5	24
2	6	15/18	5	25
	7	15/18	5	26
	8	15/18	5	27
	9	15/18	5	28
	10	15/18	5	29
3	11	15/18	10	30
	12	15/18	10	31
	13	15/18	10	32
	14	15/18	10	33
	15	15/18	10	34
4	16	18/21	10	35
	17	18/21	10	36
	18	18/21	10	37
	19	18/21	10	38
	20	18/21	10	39
5	21	20/23	10	40

	22	20/23	10	41
	23	20/23	10	42
	24	20/23	10	43
	25	20/23	10	44
6	26	23/26	10	45
	27	23/26	10	46
	28	23/26	10	47
	29	23/26	10	48
	30	23/26	10	49
7	31	25/28	10	50
	32	25/28	10	50
	33	25/28	10	50
	34	25/28	10	50
	35	25/28	10	50
8	36	25/28	10	50
	37	25/28	10	50
	38	25/28	10	50
	39	25/28	10	50
	40	25/28	10	50

Body composition

Body composition was determined for all rats 13 days prior to the start of training period using the minispec LF90II Body Composition Rat and Mice Analyzer (Bruker, 6.2 MHz Time Domain Nuclear Magnetic Resonance (TD-NMR) system). This analyzer was used for *in vivo* measurement of lean tissue, body fat, and body fluid in fully awake animals. Post-training body composition was determined for rats in the 4- and 8-week training groups, 5 days prior to tissue harvesting.

Maximum oxygen consumption (VO_2max)

VO_2max was determined prior to the onset of training in all rats, and during the last week of training, for the 4- and 8-week exercise groups. Rats were acclimated to a single-lane enclosed treadmill (Columbus Instruments Metabolic Modular Treadmill) two days prior to testing. On the day of testing the rat was placed in the treadmill and testing began once oxygen consumption stabilized. Testing began with a warm up for 15 minutes with the treadmill set at a speed of 9 m/min and 0° incline. Following the warm up period, the incline was increased to 10° and treadmill speed was increased by 1.8 m/min every 2 minutes[48]. During the test, shock was used sparingly. Shock was used only when the rat stopped running and sat on the shock area. Testing stopped when the rat sat on the shock area 3 consecutive times and did not respond to increased shock. Upon cessation of the test, the rat was removed from the enclosure and blood was taken from the tail to measure lactate. Criteria for reaching VO_2max was a leveling off of oxygen uptake despite increased workload, a respiratory exchange ratio above 1.05, and an unhemolyzed blood lactate concentration ≥6 mM.

Tissue collection

Tissues were collected from all rats 48 hours following the last exercise bout. On the day of collection, food was removed at 8:30 AM, three hours prior to the start of dissections, which occurred between 11:30 AM and 2:30 PM. Rats were sedated with inhaled isoflurane (1-2%). Under isoflurane anesthesia, blood was drawn via cardiac puncture. Under continued isoflurane anesthesia, the right triceps surae muscles (soleus, gastrocnemius, and plantaris), subcutaneous white fat on the right side, right lobe of the liver, heart, and lungs were removed in that specific order. Removal of the heart resulted in death. Immediately following removal of the heart, a guillotine was used for decapitation. The brain was removed from the skull, and the following regions were dissected out in the specified order: hypothalamus, right and left hippocampus, right and left cerebral cortex. Following decapitation, specific organs were removed in the following order: right kidney, right & left adrenal glands, spleen, brown adipose tissue, small intestine (jejunum), colon (transverse and descending) and feces, right testes or ovaries, right vastus lateralis, and tibia. All tissues were flash-frozen in liquid nitrogen immediately upon removal, placed in cryovials, and stored at -80°C. All tissues were subsequently shipped on dry ice to the Biospecimens Repository at the University of Vermont.

Sample distribution

Archiving

Frozen tissue samples received by the Biospecimens Repository at the University of Vermont were logged in to Freezerworks (Dataworks Development Inc., Mountlake Terrace, Washington) and stored in -80°C freezers. All samples were labeled with a unique 11-digit barcode.

Selection of animals for molecular assays

Tissue samples were collected for between 12 and 20 animals per sex, per training group (i.e., sedentary controls or animals trained for 1, 2, 4, or 8 weeks). To reduce the effects of inter-animal variation on data integration, the omic assays were performed on tissues from the same individual animals whenever possible. Therefore, six animals per sex per training group were randomly selected for molecular profiling; all six replicates were used for proteomic assays; a subset of five replicates were used for genomic and metabolomic assays. Additional rats were selected at random if there were insufficient aliquots for specific molecular assays, as was the case for the adrenal gland, brown adipose tissue, and ovary immunoassays. Exact sample sizes per assay, tissue, sex, and exercise training time point are provided in Extended Data Figure 1f.

Cryopulverization

Tissues samples were stored at -80°C until time of processing. Frozen tissue samples were transferred from storage vials to Covaris tissueTUBEs (Covaris, Inc, Woburn, Massachusetts) flash-chilled in liquid nitrogen. Larger tissues were first broken into smaller pieces on a chilled, foil-covered stainless steel block using a foil-covered hammer or pestle. The tissue piece(s)

were centered in the tissueTUBE pouch (primary impact zone), and a pre-chilled glass transfer tube was attached. Filled tissueTUBEs were placed on dry ice while the Covaris CryoPREP CP02 (Covaris Inc, Woburn, Massachusetts) was set to the appropriate setting for the tissue type. TissueTUBEs were dipped in liquid nitrogen, and the glass transfer tube was loosened ¼ turn to prevent pressure build-up inside the tissueTUBE before being inserted in the Covaris CP02 to pulverize. Tissues requiring an additional round of pulverization were first inspected to ensure tissueTUBE integrity and dipped in liquid nitrogen before being put back into the Covaris CP02. After pulverization, tissueTUBEs were dipped in liquid nitrogen and then inverted to move the pulverized sample into the glass transfer tube. Pulverized samples were transferred to cryogenic storage vials for long term storage at -80°C.

Aliquoting

Frozen tissue storage vials were removed from the -80°C freezer and placed on dry ice. Aliquot vials were set up in a prechilled CoolRack XT CFT24 (Corning, Corning, New York) sitting in dry ice inside an AirClean 600 Dead Air Workstation (AirClean Systems, Creedmoor, North Carolina). Working with one storage vial at a time, disposable plastic transfer scoops of predetermined volumes were chilled in liquid nitrogen and then used to measure and transfer tissue to aliquot vials. A chilled metal spatula was used to break up any tissue clumps in the storage tubes before aliquoting. Aliquot vials were capped and stored at -80°C before shipping to chemical analysis labs. By experimentation we determined that the target weights could be aliquoted with a reproducibility of +/- 15% of the target. Different sized scoops were needed for different tissues depending on fat content.

Reference standards

Aliquots of assay- and tissue-specific reference standards were included in molecular assays in order to evaluate technical differences across batches. The pooled reference standards used for RNA-Seq, ATAC-seq, RRBS, and immunoassays were from non-compliant animals collected at the University of Florida for a separate MoTrPAC study. Samples from the same tissue and sex were pulverized together to create a homogenous pool. One aliquot each of the male and female reference standards was included on each plate of study samples. Tissue-matched reference standards were used when possible. Metabolomics and lipidomics reference standards were from pilot samples collected at the University of Florida, Joslin Diabetes Center, and the University of Iowa for a separate MoTrPAC study. Samples were split to create pools of sedentary and immediate-post-exercise reference standards. One aliquot each of the sedentary and exercised reference standards was included with each batch of study samples. For proteomics, a universal reference standard was generated within each tissue type by pooling equal amounts of peptide from each experimental sample. All samples were from Fischer 344 (F344) inbred rats obtained from the NIA rodent colony.

DATA PRODUCTION AND QUANTIFICATION

DNA methylation by reduced representation bisulfite sequencing

DNA extraction and library preparation

Reduced representation bisulfite sequencing (RRBS)[49] was performed at the Icahn School of Medicine at Mount Sinai. Rat tissues (10-30 mg of white adipose tissue) were disrupted in GenFind v2 lysis buffer (Beckman Coulter, Indianapolis, IN) using a tissue ruptor (Omni International, Kennesaw, GA) and the genomic DNA was extracted in a BiomekFx automation workstation (Beckman Coulter, Chaska, Minnesota), according to the manufacturer's instructions. Two tissue-specific consortium reference standards were included to monitor the sample processing QC. DNA samples were quantified by Qubit assay (dsDNA HR assay, Thermo Fisher Scientific), and the quality was determined by the Nanodrop A260/280 and A260/230 ratios.

The Ovation® RRBS Methyl-Seq kit from Tecan Genomics (Baldwin Park, CA) was used to generate the RRBS libraries according to the manufacturer's instructions. All library preparations were carried out in an automated workstation (Biomek Fx, Beckman Coulter). Briefly, purified genomic DNA (100 ng) was digested by the methylation-insensitive restriction enzyme MspI to generate short fragments that contain CpG dinucleotides at the ends. After end-repair, A-tailing, and ligation to methylated Illumina adapters, the CpG-rich DNA fragments (40-220 base pairs) were size-selected and subjected to bisulfite conversion according to the manufacturer's instructions. The single-stranded uracil-containing DNA was purified after desulfonation using the magnetic beads provided in the kit, and eluted DNA was converted to double-stranded DNA by PCR with the following cycling parameters: 95°C for 2 minutes, followed by the optimal number of cycles of 95°C for 15 seconds, 60°C for 1 minute, and 72°C for 30 seconds, and a final step at 72°C for 10 minutes. The optimal number of cycles for the enrichment PCR was calculated based on qPCR values. A final clean-up step was performed by 1x AMPure XP beads. The quantity of the libraries was measured by Qubit High Sensitivity assays (ThermoFisher Scientific), and the quality was evaluated using Bioanalyzer High Sensitivity DNA Chip (Agilent Technologies, Santa Clara, CA). The libraries were pooled and sequenced in a MiSeq Nano flow cell to determine the sequencing quality of the libraries. Unmethylated lambda DNA (Promega, D1521) was spiked (0.05%) in each sample to monitor bisulfite conversion. Jurkat genomic DNA (ThermoFisher, SD1111) and CpG Methylated Jurkat genomic DNA (ThermoFisher, SD1121) were used as positive controls with each batch.

DNA sequencing, data processing, and normalization

Sequencing of RRBS libraries was performed on a NovaSeq 6000 platform (Illumina, San Diego, CA, USA) using a paired-end 100 base-pair run configuration. The pooled libraries were spiked in with 10% PhiX and sequenced to a minimal depth of 30 million paired-end reads per library using a custom 1-index primer as per Illumina guidelines. In addition to the 8-base barcode, the adapter contained 8-base unique molecular identifiers (UMIs) immediately

following the library index. The UMIs were used for duplicate read determination (see below). To take advantage of this feature, the libraries were sequenced using 16 cycles for the i7 index read.

Reads were demultiplexed with bcl2fastq (version 2.20) using options --use-bases-mask Y*,I8Y*,I*,Y* --mask-short-adapter-reads 0 --minimum-trimmed-read-length 0 (Illumina, San Diego, CA, USA), and UMIs in the index FASTQ files were attached to the read FASTQ files. The regular 5' and 3' adapters were trimmed with TrimGalore (v1.18), and the diversity adapter that is about 0 to 3 bases of RDD (R={A or G} and D={A, G, or T}) that is added before YGG (Y={C or T} depending on the methylation) from the YGG MspI cut signature was trimmed with the NuGEN script "trimRRBSdiversityAdaptCustomers.py" (https://github.com/nugentechnologies/NuMetRRBS). FastQC (v0.11.8) was used to generate pre-alignment QC metrics[50]. Bismark (v0.20.0) was used to index and align reads to release 96 of the Ensembl *Rattus norvegicus* (rn6) genome and gene annotation[51]. As the lambda genome was spiked into each sample to determine the bisulfite conversion efficiency, the lambda genome (GenBank: J02459.1) was also indexed. Default parameters were used for Bismark's bismark_genome_preparation in the alignment step. Bismark output BAM files were first formatted using a custom script; Bismark's deduplicate_bismark with "-p --barcode" options was used to remove PCR duplicates from the bam files; and Bismark's "bismark_methylation_extractor --comprehensive --bedgraph" was used to quantify methylated and unmethylated coverages for all the CpG sites. Bowtie 2 (v2.3.4.3) was used to index and align reads to globin, rRNA, and phix sequences in order to quantify the percent of reads that mapped to these contaminants and spike-ins[52]. SAMtools (v1.3.1) was used to compute mapping percentages to different chromosomes[53]. UMIs were used to accurately quantify PCR duplicates with NuGEN's "nodup.py" script (https://github.com/tecangenomics/nudup). QC metrics from every stage of the quantification pipeline were compiled, in part with multiQC (v1.6)[54]. The openWDL-based implementation of the RRBS pipeline on Google Cloud Platform is available on GitHub[43]. Only CpG sites with methylation coverage of >=10 in all samples were included for downstream analysis, and normalization was performed separately in each tissue. Individual CpG sites were divided into 500 base-pair windows and were clustered using the Markov Clustering algorithm via the *MCL* R package[55]. To apply MCL, for each 500 base-pair window an undirected graph was constructed, linking individual sites if their correlation was >=0.7. MCL was chosen for this task as it: (1) determines the number of clusters internally, (2) identifies homogeneous clusters, and (3) keeps single sites that are not correlated with either sites as singletons (clusters of size one). The resulting sites/clusters were used as input for normalization and differential analysis with edgeR[56]. To generate normalized sample-level data, the methylation coverages of filtered sites/clusters were first $\log_2$-transformed, and normalization was performed using preprocessCore's quantile normalization *preprocessCore::normalize.quantiles.robust*[57]. As all samples for a given tissue were processed in a single batch, batch correction was not possible or necessary.

Assay for transposase-accessible chromatin using sequencing

Nuclei extraction and library preparation

Assay for transposase-accessible chromatin using sequencing (ATAC-seq)[58] was performed at Stanford University and the Icahn School of Medicine at Mount Sinai. Only Stanford ATAC-seq data were used in this manuscript. All sample processing steps were performed in batches of less than 12 samples. Nuclei from aliquoted tissue samples (30 mg for white adipose, 15 mg for brown adipose, 10 mg for hippocampus, kidney, lung, gastrocnemius, heart, and liver tissues) were extracted using the Omni-ATAC protocol with modifications[59]. Two tissue-specific consortium reference standards were included for sample processing QC. The white adipose, brown adipose, and hippocampus tissues were processed using no-douncing nuclei extraction to prevent fat droplets from interfering with the subsequent transposition steps. Tissue powder was incubated in the homogenization buffer for 10 min at 4°C. The tube was inverted every 2-3 minutes. The heart, liver, kidney, lung, and gastrocnemius tissues were processed using the Omni-ATAC protocol with modifications. Tissue powder was incubated in the homogenization buffer for 5 minutes on ice and dounced 10 times using pestle A and 20 times with pestle B. For both protocols, the homogenate passed through a 40 µm cell strainer to collect nuclei. Nuclei were stained with DAPI and counted using an automated cell counter. 50,000 nuclei (or max. 500 µl nuclei) were added to 1 ml ATAC-RSB buffer and spun at 1000 g for 10 minutes, and the supernatant was removed. The nuclei pellet was resuspended in 50 µl of transposition mixture and incubated at 37°C for 30 minutes with 1000 rpm shaking. The transposed DNA was purified using Qiagen MinElute Purification kits (Qiagen # 28006). The DNA product was amplified using NEBnext High-Fidelity 2x PCR Master Mix (NEB, M0541L) and custom indexed primers. 1.8x SPRIselect beads were used to clean the PCR reaction and obtain the ATAC-seq library for sequencing.

DNA sequencing, data processing, and normalization

Pooled libraries were sequenced on an Illumina NovaSeq 6000 platform (Illumina, San Diego, CA, USA) to a target depth of 35 million read pairs (70 million paired-end reads) per sample using a paired-end 50 base-pair run configuration. Reads were demultiplexed with bcl2fastq2 (v2.20.0) (Illumina, San Diego, CA, USA). Data was processed with the ENCODE ATAC-seq pipeline (v1.7.0)[60]. Samples from a single sex and training time point, e.g., males trained for 2 weeks, were analyzed together as biological replicates in a single workflow. Briefly, adapters were trimmed with cutadapt v2.5[61] and aligned to release 96 of the Ensembl *Rattus norvegicus* (rn6) genome[51] with Bowtie 2 v2.3.4.3[52]. Duplicate reads and reads mapping to the mitochondrial chromosome were removed. Signal files and peak calls were generated using MACS2 v2.2.4[62], both from reads from each sample and pooled reads from all biological replicates. Pooled peaks were compared with the peaks called for each replicate individually using Irreproducibility Discovery Rate[63] and thresholded to generate an optimal set of peaks. The cloud implementation of the ENCODE ATAC-seq pipeline and source code for the post-processing steps are available on Github[42]. Optimal peaks (overlap.optimal_peak.narrowPeak.bed.gz) from all workflows were concatenated, trimmed to

200 base pairs around the summit, and sorted and merged with bedtools v2.29.0[64] to generate a master peak list. This peak list was intersected with the filtered alignments from each sample using bedtools coverage with options -nonamecheck and -counts to generate a peak by sample matrix of raw counts. The remaining steps were applied separately on raw counts from each tissue. Peaks from non-autosomal chromosomes were removed, as well as peaks that did not have at least 10 read counts in four samples. Filtered raw counts were then quantile-normalized with limma-voom[65]. This version of the normalized data was used for downstream analyses. Batch correction was performed by including the sample processing batch, coded as a factor, as a covariate during differential analysis.

RNA sequencing

Extraction of total RNA

RNA sequencing (RNA-Seq) was performed at Stanford University and the Icahn School of Medicine at Mount Sinai. Rat tissues (30 mg for white adipose, 15 mg for brown adipose, 10 mg for other solid tissues, and 0.47 ml blood) were disrupted in Agencourt RNAdvance tissue lysis buffer (Beckman Coulter, Brea, CA) using a tissue ruptor (Omni International, Kennesaw, GA, # 19-040E). Total RNA was extracted in a BiomekFx automation workstation according to the manufacturer’s instructions for tissue-specific extraction. Total RNA from blood collected in PAXgene tubes (BD Biosciences, Franklin Lakes, NJ, # 762165) was extracted using the Agencourt RNAdvance blood specific kit (Beckman Coulter). Two tissue-specific consortium reference standards were included to monitor the sample processing QC. The RNA was quantified by NanoDrop (ThermoFisher Scientific, # ND-ONE-W) and Qubit assay (ThermoFisher Scientific), and the quality was determined by either Bioanalyzer or Fragment Analyzer analysis.

mRNA Sequence Library Preparation

Universal Plus mRNA-Seq kit from NuGEN/Tecan (# 9133) were used for generation of RNA-Seq libraries derived from poly(A)-selected RNA according to the manufacturer’s instructions. Universal Plus mRNA-Seq libraries contain dual (i7 and i5) 8 bp barcodes and an 8 bp unique molecular identifier (UMI), which enable deep multiplexing of NGS sequencing samples and accurate quantification of PCR duplication levels. Approximately 500ng of total RNA were used to generate the libraries. The Universal Plus mRNA-Seq workflow consists of poly(A) RNA selection, RNA fragmentation and double-stranded cDNA generation using a mixture of random and oligo(dT) priming, end repair to generate blunt ends, adaptor ligation, strand selection, AnyDeplete workflow to remove unwanted ribosomal and globin transcripts, and PCR amplification to enrich final library species. All library preparations were performed using a Biomek i7 laboratory automation system (Beckman Coulter). Tissue-specific reference standards provided by the consortium were included with all RNA isolations to QC the RNA.

RNA sequencing, quantification, and normalization

Pooled libraries were sequenced on an Illumina NovaSeq 6000 platform (Illumina, San Diego, CA, USA) to a target depth of 35 million read pairs (70 million paired-end reads) per sample using a paired-end 100 base pair run configuration. In order to capture the 8-base UMIs, libraries were sequenced using 16 cycles for the i7 index read and 8 cycles for the i5 index read. Reads were demultiplexed with bcl2fastq2 (v2.20.0) using options --use-bases-mask Y*,I8Y*,I*,Y* --mask-short-adapter-reads 0 --minimum-trimmed-read-length 0 (Illumina, San Diego, CA, USA), and UMIs in the index FASTQ files were attached to the read FASTQ files. Adapters were trimmed with cutadapt (v1.18), and trimmed reads shorter than 20 base pairs were removed[61]. FastQC (v0.11.8) was used to generate pre-alignment QC metrics[50]. STAR (v2.7.0d) was used to index and align reads to release 96 of the Ensembl Rattus norvegicus (rn6) genome and gene annotation[51]. Default parameters were used for STAR's genomeGenerate run mode; in STAR's alignReads run mode, SAM attributes were specified as NH HI AS NM MD nM, and reads were removed if they did not contain high-confidence collapsed splice junctions (--outFilterType BySJout). RSEM (v1.3.1) was used to quantify transcriptome-coordinate-sorted alignments using a forward probability of 0.5 to indicate a non-strand-specific protocol[66]. Bowtie 2 (v2.3.4.3) was used to index and align reads to globin, rRNA, and phix sequences in order to quantify the percent of reads that mapped to these contaminants and spike-ins[52]. UCSC's gtfToGenePred was used to convert the rn6 gene annotation (GTF) to a refFlat file in order to run Picard CollectRnaSeqMetrics (v2.18.16) with options MINIMUM_LENGTH=50 and RRNA_FRAGMENT_PERCENTAGE=0.3[67]. UMIs were used to accurately quantify PCR duplicates with NuGEN's "nodup.py" script (https://github.com/tecangenomics/nudup). QC metrics from every stage of the quantification pipeline were compiled, in part with multiQC (v1.6)[54]. The openWDL-based implementation of the RNA-Seq pipeline on Google Cloud Platform is available on Github[41]. Filtering of lowly expressed genes and normalization were performed separately in each tissue. RSEM gene counts were used to remove lowly expressed genes, defined as having 0.5 or fewer counts per million in all but one sample. These filtered raw counts were used as input for differential analysis with DESeq2[68], as described below. To generate normalized sample-level data, filtered gene counts were TMM-normalized using *edgeR::calcNormFactors*, followed by conversion to log counts per million with *edgeR::cpm*[56]. As all samples for a given tissue were processed in a single batch, batch correction was not possible or necessary.

Liquid chromatography tandem mass spectrometry (LC-MS/MS) proteomics

Tissue lysis and protein extraction

LC-MS/MS analyses of 60 samples per tissue representing full time course samples for 6 female and 6 male rats were performed at the Broad Institute of MIT and Harvard (heart and liver) and the Pacific Northwest National Laboratory (gastrocnemius, white adipose tissue, cerebellum, lung, and kidney). Sample processing for proteomic analysis was based on a protocol previously described[69], with modifications detailed below. Cryopulverized tissue

samples were suspended in cold, freshly-prepared lysis buffer (8 M urea (Sigma-Aldrich, St. Louis, Missouri), 50 mM Tris pH 8.0, 75 mM sodium chloride, 1 mM EDTA, 2 µg/ml Aprotinin (Sigma-Aldrich, St. Louis, Missouri), 10 µg/ml Leupeptin (Roche CustomBiotech, Indianapolis, Indiana), 1 mM PMSF in EtOH, 10 mM sodium fluoride, 1% phosphatase inhibitor cocktail 2 and 3 (Sigma-Aldrich, St. Louis, Missouri)), vortexed for 10 seconds, and incubated for 15 min on a thermomixer set to 4°C and 800 rpm. The lysis buffer for heart and liver tissues was supplemented with deacetylase inhibitors for acetylome analysis (10 mM Sodium Butyrate, 2 µM SAHA, and 10 mM nicotinamide). Samples were vortexed for an additional 10 seconds and incubated for 15 more minutes with the same settings, then centrifuged for 10 minutes at 4°C and 18000 rcf to remove debris. Supernatant was removed, and protein concentrations were determined by BCA assay (ThermoFisher, Waltham, Massachusetts).

Protein digestion

Protein lysate concentrations were normalized within samples of the same tissue type, and protein was reduced with 5 mM dithiothreitol (DTT, Sigma-Aldrich) for 1 hour in a thermomixer set to 37°C and 1000 rpm. Protein was then alkylated with iodoacetamide (IAA, Sigma-Aldrich) for 45 minutes in the dark in a thermomixer set to 25°C and 1000 rpm. Samples were then diluted 1:4 with 50 mM Tris-HCl, pH 8.0 to lower the urea concentration below 2 M. LysC endopeptidase (1mAU/uL, Wako Chemicals, Richmond, Virginia) was added at a 1:50 enzyme:substrate ratio, and samples were then digested for 2 hours in a thermomixer set to 25°C and 850 rpm. Sequencing grade modified trypsin (Promega, MAdison, Wisconsin) was then added at an enzyme:substrate ratio of 1:50 (or 1:10 for white adipose tissue), and samples were digested for 14 hours in a thermomixer set to 25°C and 850 rpm. Digestions were quenched by adding formic acid (FA) to a final concentration of 1%, and samples were centrifuged at 1500 rcf for 15 minutes at 4°C. Supernatants were collected into new tubes, diluted to 3 ml total volume with 0.1% FA, and desalted using Sep-Pac C18 SPE cartridges (Waters, Milford, MA). Clean peptides were concentrated in a speedvac, and peptide concentrations were determined by BCA assay.

Tandem mass tag (TMT) labeling of peptides

400 µg aliquots of every sample were prepared and dried down to be used for TMT labeling. Additionally, a common reference sample was generated within each tissue type by pooling equal amounts of peptide from each experimental sample; one 400 µg aliquot of this reference was included in each multiplex. Within each tissue type male and female rat time course samples were randomized into 6 TMT11 plexes making sure single time course samples were in the same plex and keeping 1 male and 1 female in each plex. Samples were resuspended in 200 mM HEPES pH 8.5 to a final concentration of 5 µg/uL for labeling and a reduced TMT reagent labeling approach was used[70]. Experimental samples were randomized across the first ten TMT channels (126C to 131N) and common reference aliquots were assigned to the last TMT channel (131C). TMT reagents were resuspended in anhydrous acetonitrile to a concentration of 20 µg/uL, and 400 µg of reagent was added to appropriate peptide aliquots (resulting in a 1:1 peptide:tag ratio). Labeling was allowed to proceed for 1 hour in a thermomixer set to 25°C and 400 rpm. After 1 hour, samples were diluted to 2.5 µg/uL with 20%

acetonitrile, a small aliquot was removed from each sample for labeling efficiency and mixing tests, and the remaining sample was snap-frozen in liquid N_2 and stored at -80°C. Following QC checks, reactions were quenched with hydroxylamine and samples from each multiplex were combined, concentrated in a speedvac, and desalted using Sep-Pac C18 SPE cartridges (Waters). Heart and liver samples were subjected to phosphotyrosine enrichment, while this step was skipped for the remaining tissues to perform offline bRP fractionation.

Phosphotyrosine peptide enrichment

Phosphotyrosine (pY) enrichment was performed on liver and heart tissues for improved characterization of tyrosine kinase signaling pathways, as described previously[71]. TMT-labeled and combined peptides were resuspended in 1.5 ml of IAP buffer (50 mM MOPS, 10 mM sodium phosphate dibasic, 50 mM NaCl) and cleared by centrifugation (5 min, 5000 rcf). The pellet was dissolved in 1% FA and combined with the pY enrichment flowthrough for downstream bRP fractionation, while the supernatant was used for pY enrichment. PTMScan Phospho-Tyrosine Rabbit mAb beads (Cell Signaling Technologies #8803, Danvers, Massachusetts) were washed three times with 1.5 ml IAP buffer (30 s, 2000 rcf) and incubated with the solubilized peptides (1 hour, 4°C, end-over-end rotation). Beads with pY peptides bound were centrifuged (1 minute, 1500 rcf), and the flowthrough was collected, desalted using Sep-Pac C18 SPE cartridges, and used for downstream bRP offline fractionation. The beads were washed four times using 1.5 ml PBS (30 seconds, 2000 rcf) and pY peptides were eluted by two consecutive 5-minute incubations with 0.15% trifluoroacetic acid (TFA). Eluted pY peptides were desalted using stage tips containing 2x C18 discs (Empore, CDS Analytical, Oxford, Pennsylvania). The stage tip column was conditioned with 1x 100 µL methanol, 1x 100 µL 50% acetonitrile (ACN) / 1% FA, and 2x 100 µL 0.1% FA washes. Acidified peptides were bound to the column, washed with 2x 100 µL 0.1% FA, and eluted with 50 µL 50% ACN / 0.1% FA. Eluted peptides were dried using a vacuum concentrator. Phosphotyrosine peptides were suspended in 9 µl of 0.1% FA and 3% ACN, and 2 x 4 µl were injected in an LC-MS/MS instrument.

Offline bRP fractionation

Each combined multiplex sample was fractionated by high pH reversed phase separation using a 3.5 µm Agilent Zorbax 300 Extend-C18 column (4.6 mm ID x 250 mm length). Samples were resuspended in mobile phase A (5 mM ammonium formate, pH 10, in 2% acetonitrile), centrifuged to remove debris, loaded onto the column and eluted off the column for 96 minutes at a flow rate of 1 ml/minute using mobile phase B (5 mM ammonium formate, pH 10, in 90% acetonitrile) with the following gradient (time(min): %B): 0:0; 7:0; 13:16; 73:40; 77:44; 82:60; 96:60. A total of 96 fractions were collected, immediately acidified to 0.1% formic acid, and concatenated down to 24 fractions by combining non-sequential fractions. For heart and liver tissues, the complete gradient was collected and only fractions 15-91 showing robust peptide signals were concatenated. Fractions 3-14 were collected and used as an extra fraction “A” for phosphopeptide enrichment. For the remaining tissues, 96 fractions were collected between elution time of 2.4 to 91 minutes and all fractions were used for concatenation. Five percent was removed for global proteome analysis. For phosphopeptide enrichment, the remaining 95% was

further concatenated to 12 fractions plus an additional fraction “A” for heart and liver only. These fractions were frozen in liquid nitrogen, vacuum-centrifuged to dryness, and stored at -80°C until ready for phosphopeptide enrichment.

Phosphopeptide enrichment

Phosphopeptide enrichment was performed through immobilized metal affinity chromatography (IMAC) using Fe^{3+}-NTA-agarose beads, freshly prepared from Ni-NTA-agarose beads (Qiagen, Hilden, Germany) by sequential incubation in 100 mM EDTA to strip nickel, washing with HPLC water, and incubation in 10 mM iron (III) chloride). Peptide fractions were resuspended to 0.5 µg/uL in 80% ACN + 0.1% TFA and incubated with beads for 30 minutes in a thermomixer set to 1000 rpm at room temperature. After 30 minutes, beads were spun down (1 minute, 1000 rcf) and supernatant was removed and saved as flow-through for subsequent enrichments. Phosphopeptides were eluted off IMAC beads in 3x 70 µl of agarose bead elution buffer (500 mM K_2HPO_4, pH 7.0), desalted using C18 stage tips, eluted with 50% ACN, and lyophilized. Samples were then reconstituted in 3% ACN / 0.1% FA for LC-MS/MS analysis (12 µl reconstitution / 5 µl injection for gastrocnemius, white adipose, lung, kidney, and cortex samples; 9 µl reconstitution / 4 µl injection for heart and liver samples). Flow-through from the 12 IMAC fractions were further concatenated into 4 fractions to be used for acetylpeptide enrichment only for heart and liver tissues.

Acetylpeptide enrichment

Acetylated lysine peptides were enriched using an antibody against the acetyl-lysine motif (Cell Signaling Technologies #13416, Danvers, Massachusetts). Peptide fractions were reconstituted with 1.4 ml of IAP buffer per fraction and incubated for 2 h at 4°C with pre-washed (3 times with IAP buffer) agarose beads bound to acetyl-lysine motif antibody. Peptide-bound beads were washed 4 times with ice-cold PBS followed by elution with 2 × 100 µl of 0.15% TFA. Eluents were desalted using C18 stage tips, eluted with 50% ACN, lyophilized, and reconstituted in 9 µl 3% ACN / 0.1% FA. Four µl of each fraction were injected for LC-MS/MS analysis.

K-ε-GG enrichment for ubiquitylome analysis

Ubiquitin enrichment was performed based on the UbiFast protocol[72]. Anti-K-ε-GG bead-bound antibodies from the PTM-Scan ubiquitin remnant motif kit (Cell Signaling Technologies #5562) were cross-linked using the following procedure. Beads were washed 3 times with 100 mM sodium borate (pH 9.0) and incubated with 20 mM DMP for 30 minutes at room temperature. Beads were then washed 2 times with 200 mM ethanolamine and incubated overnight at 4°C in 200 mM ethanolamine with end-over-end rotation. Following incubation, beads were washed three times with an IAP buffer and stored at 4°C at a concentration of 0.5 µg/µL. For each 11-plex experiment, 31.25 µg of cross-linked anti-K-ε-GG bead-bound antibody at 0.5 µg/µL in IAP per channel was aliquoted into 1.5 ml Eppendorf tubes on ice. Eight hundred and one thousand micrograms of total peptides were used for liver and heart tissue samples, respectively. Each sample was reconstituted to 0.5 mg/ml concentration in IAP buffer and vortexed for 10 minutes. Peptides were then centrifuged for 5 minutes at 5000 g. Each peptide

solution was added to a tube of antibody and gently rotated end-over-end at 4°C for 1 hour. Following enrichment, samples were centrifuged (1 minute, 2000 rcf) and the supernatant was removed. Beads were washed with 1.5 ml ice cold IAP followed by 1.5 ml ice cold PBS (30 seconds, 2000 rcf) and reconstituted in 200 µl 100 mM HEPES buffer. For each sample, 400 µg of TMT labeling reagent in 10 µl acetonitrile was added. For heart samples, 800 µg of TMT labeling reagent was added to improve labeling efficiency. Peptides were TMT labeled on-beads while shaking vigorously (1400 rpm) at 20°C for 10 minutes, then quenched with 8 µl 5% hydroxylamine and shaken vigorously for another 5 minutes, washed once with 1.3 ml cold IAP, and again with 1.5 ml cold IAP. Samples for each channel were resuspended and transferred to a combination tube with 130 µl cold IAP. Following combination, each now-empty tube was serially washed with 1.5 ml cold IAP to remove remaining beads. This 1.5 ml IAP fraction was added to the combination tube and used to wash the combined beads. Combined beads were then washed one final time with 1.5 ml ice cold PBS. Once the channels were combined and washed, peptides were eluted twice from the beads by resuspending with 150 µL room-temperature 0.15% TFA and incubating for 5 minutes at room temperature. Each round of acid-eluted K-ε-GG-modified peptides was desalted on an equilibrated two-punch C18 stage tip. Both elutions of K-ε-GG peptides were loaded sequentially, washed 2 times with 100 µl 0.1% FA, and eluted into an LC-MS vial with 50 µl 50% ACN / 0.1% FA. The eluted peptides were frozen, lyophilized, and reconstituted in 9 µl 3% ACN / 0.1% FA, with 4 µl injected twice for two consecutive LC-MS/MS runs.

LC-MS/MS analysis of heart and liver tissue samples

Online separation was conducted with a nanoflow Proxeon EASY-nLC 1200 UHPLC system (Thermo Fisher Scientific). In this setup, the LC system, column, and platinum wire used to deliver the electrospray source voltage were connected via a stainless steel cross (360 mm, IDEX Health & Science, UH-906x). An in-house packed 22 cm x 75 µm internal diameter C18 silica picofrit capillary column (1.9 mm ReproSil-Pur C18-AQ beads, Dr. Maisch GmbH, r119.AQ; Picofrit 10 µm tip opening, New Objective, PF360-75-10-N-5) heated at 50°C using a column heater sleeve (Phoenix-ST) was used for chromatographic separation. For global proteome analysis, ~1 µg was loaded on-column in a 2-µL volume (based on peptide-level BCA with uniformly-distributed fractionation presumed). For phosphoproteome, acetylome, and ubiquitylome analysis, 50% of each fraction sample was injected in a 4-µl volume. Mobile phase flow rate was 200 nL/min, composed of 3% acetonitrile / 0.1% formic acid (Solvent A) and 90% acetonitrile / 0.1% formic acid (Solvent B). The 110-minute LC-MS/MS method used for global proteome and IMAC phosphoproteome analysis consisted of a 10-minute column-equilibration procedure; a 20-minute sample-loading procedure; and the following gradient profile: (time (minutes):%B) 0:1; 1:6; 63:20, 85:30; 94:60; 95;90; 100:90; 101:50; 110:50. For acetylproteome, ubiquitylome, and phosphotyrosine enrichment analysis, the same LC and column setup was used, but the gradient was extended to 154 minutes with the following gradient profile: (time (minutes):%B) 0:2; 2:6; 122:30; 130:60; 133;90; 143:90; 144:50; 154:50.

For proteome analysis, samples were analyzed with a Q-Exactive Plus mass spectrometer (Thermo Fisher Scientific). Data-dependent MS/MS acquisition was performed using the following relevant parameters: positive ion mode at a spray voltage of 1.8 kV, MS1 resolution of

60,000, an AGC target of 3e6, a mass range from 300 to 1800 *m/z*, MS2 resolution of 45,000, MS2 AGC target of 5e4, isolation window of 0.7 *m/z*, MS2 maximum injection time of 105 ms, HCD collision energy of 29%, dynamic exclusion for 20 seconds, peptide match was set to preferred for monoisotopic peak determination, charge states 2-6.

For analysis of IMAC-enriched peptides (phosphoproteome), samples were analyzed with a Q-Exactive HFX mass spectrometer (Thermo Fisher Scientific). Data-dependent MS/MS acquisition was performed using the following relevant parameters: positive ion mode at a spray voltage of 1.5 kV, MS1 resolution of 60,000, an AGC target of 3e6, a mass range from 350 to 1800 *m/z*, MS2 resolution of 45,000, MS2 AGC target of 5e4, isolation window of 0.7 *m/z*, MS2 maximum injection time of 105 ms, HCD collision energy of 31%, dynamic exclusion for 15 seconds, peptide match was set to preferred for monoisotopic peak determination, charge states 2-6. The advanced precursor determination feature (APD)[73] was turned off in the tune file using a software patch provided by Thermo Fisher Scientific (Tune version 2.11). For analysis of phosphotyrosine enriched peptides (phosphoproteome), the same settings were used with the following modifications: MS2 AGC target of 2e5 and MS2 maximum injection time of 150 ms. For analysis of acetyl-lysine enriched peptides (acetylome), the same settings were used with the following modifications: MS2 AGC target of 5e5 and MS2 maximum injection time of 150 ms. For analysis of K-ε-GG enriched peptides (ubiquitylome), the same settings were used with the following modifications: MS2 AGC target of 5e4 and MS2 maximum injection time of 150 ms.

LS-MS/MS analysis of the gastrocnemius, white adipose tissue, cortex, kidney, and lung

For global proteome analysis, online separation was performed with a nanoAcquity M-Class UHPLC system (Waters) equipped with a 250 mm x 4.6 mm internal diameter Jupiter 5 µm C18 trapping column (Phenomenex). An in-house packed, 25 cm x 75 µm internal diameter C18 silica picofrit column (1.7 µm UPLC BEH particles, Waters Acquity) heated to 50°C was used for chromatographic separation. 0.25-0.5 µg per fraction (based on peptide-level BCA) was loaded on-column in a 5uL volume. Mobile phase flow rate was 200 nL/min, composed of 0.1% formic acid in H_2O (Solvent A) and 0.1% formic acid in acetonitrile (Solvent B). Following a 6-minute trap time and 27-minute sample load time in 100% solvent A, the following 120-minute gradient profile was applied: (minutes:%B) 0:8; 83:20; 96:35; 101:75; 104:95; 110:95; 111:50; 113:95. Samples were analyzed with a Q Exactive HF mass spectrometer (Thermo Fisher Scientific). Data-dependent MS/MS acquisition was performed with the following parameters: positive ion mode at a spray voltage of 1.8 kV, MS1 resolution of 60,000, an AGC target of 3e6, a mass range from 300 to 1800 *m/z*, MS2 resolution of 30,000, MS2 AGC target of 1e5, isolation window of 0.7 *m/z*, MS2 maximum injection time of 100 ms, HCD collision energy of 30%, dynamic exclusion for 45 seconds, peptide match was set to preferred for monoisotopic peak determination, charge states 2-6.

For IMAC-enriched phosphopeptide analysis, online separation was performed with a Dionex Ultimate 3000 UHPLC direct-inject system (Thermo). An in-house packed, 30 cm x 75 µm internal diameter C18 silica picofrit column (1.7 µm UPLC BEH particles, Waters Acquity) at

room temperature was used for chromatographic separation. Samples were resuspended in 12 µl Solvent A, and 5 µl was loaded on-column. Mobile phase flow rate was 200 nL/min, composed of 0.1% formic acid in H_2O (Solvent A) and 0.1% formic acid in acetonitrile (Solvent B). Following a 60-minute sample load time in 100% solvent A, the following 120-minute gradient profile was applied: (time (minutes):%B) 0:8; 85:25; 95:35; 100:75; 105:5; 110:95; 115:1. Samples were analyzed with a Q-Exactive HF-X mass spectrometer (Thermo Fisher Scientific). Data-dependent MS/MS acquisition was performed with the following parameters: positive ion mode at a spray voltage of 1.8 kV, MS1 resolution of 60,000, an AGC target of 3e6, a mass range from 300 to 1800 *m/z*, MS2 resolution of 45,000, MS2 AGC target of 2e5, isolation window of 0.7 *m/z*, MS2 maximum injection time of 100 ms, HCD collision energy of 30%, dynamic exclusion for 45 seconds, peptide match was set to preferred for monoisotopic peak determination, charge states 2-6.

Raw MS/MS data processing

Raw MS/MS data from gastrocnemius, white adipose, lung, kidney, and cortex were processed using a cloud-based proteomics pipeline developed for this project and executed in the Google Cloud Platform. Briefly, we used the Workflow Description Language (WDL) to define the workflow and parallelize the execution using Cromwell as the workflow management system. The pipeline includes the following steps and open source methods:

1. MASIC: Extract reporter ion peaks from MS2 spectra and create Selected Ion Chromatograms for each MS/MS parent ion.
2. MSConverted: Convert Thermo .raw files to .mzML files.
3. MS-GF+: Identify peptides using a fully tryptic search (for speed).
4. MZrefiner filter in MSConvert: Use mass error histograms to in-silico re-calibrate the *m/z* values in the .mzML file.
5. PPMErrorCharter: Plot the mass error histograms before and after in-silico recalibration.
6. MS-GF+: Identify peptides using a partially tryptic search.
7. MzidToTSVConverter: Create a tab-separated value file listing peptide IDs.
8. PeptideHitResultsProcessor: Create tab-delimited files required for step 7; files contain peptide IDs, unique sequence info, and residue modification details.
9. Ascore (PTM only): Localize the position of Phosphorylation on S, T, and Y residues in phosphopeptides.

For a more extensive description, please visit the pipeline source code available on Github[44].

MS2 spectra were processed and searched against the rat RefSeq protein database (downloaded in November 2018) and common contaminants by the MS-GF+ tool[74] for peptide sequence identification. Fixed modifications were cysteine carbamidomethylation and TMT11 on N-terminal and lysine residues; variable modifications were methionine oxidation for global proteomics datasets, and S,T,Y phosphorylation for phosphoproteomics datasets. Localization of phosphorylation modifications was performed using the Ascore algorithm[75]. A target-decoy approach was used to control false discovery rate to <1% first at the peptide level and then at the protein level. For global proteomics datasets, parsimonious inference was then performed

as described by[76], with peptides being preferentially mapped to the protein with the greatest total number of peptides. For the phosphoproteomics datasets, phosphopeptides were mapped to the proteins justified through proteomics inference. Parsimonious inference was applied to the remaining set of phosphopeptides that mapped to proteins not observed in the proteomics data. This approach allows phosphopeptides to map to multiple previously justified proteins; however, it produces redundancies in the final phosphosite-level quantitation data when two or more proteins previously detected in the global results (typically isoforms) shared the same phosphosites/phosphopeptides. The degree of this redundancy is dependent on the redundancy in the database used for the MS/MS searches. TMT reporter ion intensities were extracted using MASIC[77] with the following thresholds: signal-to-noise ratio = 0; interference score = 0.5. Data from all fractions of the same multiplex were aggregated to peptide-centric, site-centric, or protein-centric levels, ratioed to the common reference, and summarized in results tables using the R package "PlexedPiper" (https://github.com/PNNL-Comp-Mass-Spec/PlexedPiper).

As a sensitivity analysis, we repeated the median MAD normalization and DEA analysis after eliminating site redundancies in the gastrocnemius, white adipose, lung, kidney, and cortex. We then compared the resulting datasets without redundancies to their matching sites in the dataset that contained the redundancies. The comparison showed that the new results were minimally impacted: correlation (ρ) of t-scores was > 0.999 in each tissue, with no site exhibiting a t-score absolute difference > 0.5 or a log-fold change absolute difference > 0.1. IHW analysis on the full phosphoproteomics dataset (i.e., including all tissues) revealed that 96% of initially significant sites at 5% remained significant, and the remaining sites tended to have a low IHW FDR (q-value < 0.11). Additionally, the PTM-SEA results were highly consistent: correlation of enrichment scores was > 0.99 for all tissue, sex, and time combinations. In summary, we observed no practical impact of removing the redundancies on the statistical and biological results.

Raw MS/MS data from heart and liver samples were processed using Spectrum Mill v.7.09.215 (Broad Institute). MS2 spectra were extracted from RAW files and merged if originating from the same precursor, or within a retention time window of +/- 60 s and *m/z* range of +/- 1.4, followed by filtering for precursor mass range of 750–6000 Da and sequence tag length > 0. MS/MS search was performed against the rat RefSeq protein database (downloaded on November 2018) and common contaminants, with digestion enzyme conditions set to "Trypsin allow P", <5 missed cleavages, fixed modifications (cysteine carbamidomethylation and TMT11 on N-term and lysine), and variable modifications (oxidized methionine, acetylation of the protein N-terminus, pyroglutamic acid on N-term Q, and pyro carbamidomethyl on N-term C). Additional variable modifications were added for analysis of phosphoproteome (S,T, and Y phosphorylation), acetylome (K acetylation), and ubiquitylome (di-glycine residual in K). Matching criteria included a 30% minimum matched peak intensity and a precursor and product mass tolerance of ± 20 ppm. Peptide-level matches were validated if found to be below the 1.0% false discovery rate (FDR) threshold and within a precursor charge range of 2–6. A second round of validation was then performed for protein-level matches for proteome datasets, requiring a minimum protein score of 13 and protein level FDR of 0%. For detailed description of peptide–spectrum matching (PSM), peptide scoring and protein grouping refer to[78,79]. For Post Translational Modifications (PTMs) PSMs were combined into a single row for all non-conflicting

observations of a particular variable modification (VM) site (e.g., different missed cleavage forms, different precursor charges, confident and ambiguous localizations, and different sample-handling modifications). All protein accession numbers which contain one of the combined peptides are reported in the row for each site.

Using the SM Autovalidation and Protein/Peptide summary modules, results were filtered and reported at the protein level. Proteins were grouped if they shared a peptide with sequence length > 8. A protein group was expanded into subgroups (isoforms or family members) if distinct peptides uniquely represented a subset of the proteins in that group. For proteomics datasets, the protein grouping method "expand subgroups, top uses shared" (SGT) was employed which allocates peptides shared by protein subgroups only to the highest scoring subgroup. For the PTM datasets, the protein grouping method "unexpand subgroups" was employed which reports a VM-site once per protein group allocated to the highest scoring subgroup containing the representative peptide. A distinct peptide is the single highest scoring instance of a peptide detected through an MS/MS spectrum. Post-translational modification (PTM) site-centric tables for PTM datasets and protein-centric tables for proteome datasets, including TMT intensity values and ratio to the common reference, were exported using Spectrum Mill and used for further normalization and statistical analysis.

Proteomics data normalization

Log_2 TMT ratios to the common reference were used as quantitative values for all proteomics features (proteins, phosphosites, acetylsite, and ubiquitylsites). Proteomics datasets were examined for sample outliers by looking at the top principal components and by examining median protein abundance across samples. Outlier samples were identified for acetylome samples labeled with channel 130C, and these were suspected to originate from contaminated 130C-TMT reagent. All acetylome samples labeled with TMT channel 130C were excluded from downstream analysis. Proteomics features not fully quantified in at least two plexes within a tissue and non-rat contaminants were removed. Log_2 TMT ratios were sample-normalized by median-centering and mean absolute deviation scaling. Plex batch effects were removed using linear models implemented by the *limma::removeBatchEffect* function in R (v 3.48.0).

The PTM datasets were corrected for changes in protein abundances by fitting a global linear model between the PTM-site and the cognate protein and extracting the residuals. However, protein-corrected PTM values were not used for most analyses due to the lack of complete overlap between PTM and total proteome features (80.5%-89.7% for phosphosites, 95.6-96.9% for acetylsites, and 94.8-95.7% for ubiquitylsites). The exception was ubiquitylome differential analysis because of the more direct effect that ubiquitination may have on protein abundance due to ubiquitin-mediated proteasomal degradation[80]. Protein-corrected values for all PTMs are included in the *MotrpacRatTraining6moData* R package for exploration[39].

Non-targeted metabolomics

HILIC LC-MS positive ion mode non-targeted metabolomics

Hydrophilic interaction liquid chromatography (HILIC) analyses of polar metabolites in the positive ionization mode were conducted at the Broad Institute of MIT and Harvard.

Sample preparation

Plasma samples: Samples were stored at -80°C until extraction and were thawed and maintained on wet ice throughout processing steps. Plasma samples (10 µL) were extracted using 90 µL of 74.9/24.9/0.2 v/v/v acetonitrile/methanol/formic acid containing valine-d8 (0.2 ng/µL, Sigma-Aldrich) and phenylalanine-d8 (0.2 ng/µL, Cambridge Isotope Laboratories) internal standards. Samples were centrifuged (10 minutes, 9,000 x g, 4°C) and supernatants were transferred to autosampler vials with deactivated glass inserts (Waters).

Tissue samples: Samples were stored at -80°C until extraction and were thawed and maintained on wet ice throughout processing steps. Powdered tissue samples (10 mg) were homogenized at 4°C in 300 µL of 10/67.4/22.4/0.018 v/v/v/v water/acetonitrile/methanol/formic acid containing valine-d8 (0.2 ng/µL, Sigma-Aldrich) and phenylalanine-d8 (0.2 ng/µL, Cambridge Isotope Laboratories) internal standards using a TissueLyser II (QIAGEN) bead mill set to two 2 min intervals at 20 Hz. Samples were centrifuged (10 minutes, 9,000 x g, 4°C) and supernatants were transferred to autosampler vials with deactivated glass inserts (Waters).

LC-MS analysis

Data were acquired using an LC-MS system comprised of a Nexera X2 UHPLC (Shimadzu) coupled to a Q-Exactive hybrid quadrupole Orbitrap mass spectrometer (Thermo Fisher Scientific). Metabolite extracts were injected onto a 150 x 2 mm, 3 µm Atlantis HILIC column (Waters). The column was eluted using the gradient program in Table 2. MS analyses were carried out using electrospray ionization in the positive ion mode using full scan analysis over 70-800 *m/z* at 70,000 resolution and 3 Hz data acquisition rate. Other MS settings were: sheath gas 40, auxiliary gas 10, sweep gas 2, spray voltage 3.5 kV, capillary temperature 350°C, S-lens RF 40, heater temperature 300°C, microscans 1, automatic gain control target 1e6, and maximum ion time 250 ms.

Table 2. HILIC-pos gradient program

Time (minutes)	%A	%B	Flow (mL/min)
0	5	95	0.25
0.5	5	95	0.25
10.5	60	40	0.25
15	60	40	0.25
17	5	95	0.25
17.5	5	95	0.25
18	5	95	0.4
30.5	5	95	0.4
31.5	5	95	0.25
32	5	95	0.25

Data processing and quality control

Feature detection and alignment: Raw data from each tissue type were processed independently using TraceFinder software (version 3.3; Thermo Fisher Scientific) for targeted peak integration and manual review of a subset of identified metabolites and Progenesis QI (Nonlinear Dynamics, Waters) for peak detection and integration of both metabolites of known identity, confirmed using reference standards, and unknowns. Data for each sample type were acquired in a single batch and unknowns were aligned using Progenesis QI.

Data cleaning and degeneracy removal: Unknown features detected and integrated using Progenesis QI were de-isotoped by the software and tacked by measured mass-to-charge ratios (m/z) and retention times (RT). In order to filter redundant ion features, Spearman correlation coefficients were calculated among all LC-MS features that eluted within a sliding retention time window of 0.025 minutes. Cliques of highly correlated, co-eluting features (Spearman correlation coefficients >0.8) were then identified. When possible, mass differences among features within each clique were used to determine ion adduct types. If an [M+H]+ ion could be identified, that feature was retained; otherwise the most abundant feature was retained. All other redundant features were flagged and removed.

Drift correction and quality control: Data quality was assured by i) initially confirming LC-MS system performance by analyzing a mixture of >140 well-characterized synthetic reference compounds as well as repeated analyses of extracts from human pooled plasma (BioIVT); ii) daily evaluation of internal standard signals to ensure that each sample injected properly and to monitor MS sensitivity; and iii) analysis of four pairs of pooled extract samples per sample type that were inserted in the analysis queue at regular intervals. One sample from each pair was used to correct for instrument drift using “nearest neighbor” scaling while the second reference sample served as a passive QC for determination of the analytical coefficient of variation of every identified metabolite and unknown.

Compound identification: Except for lipids, metabolite identities were confirmed using authentic reference standards to match measured MS and RT data in the method. Lipids were identified using representative standards and identified by RT patterns and high resolution and accurate mass MS data.

Reverse phase and ion pairing LC-MS non-targeted metabolomics

Reverse-phase and ion pairing profiling of polar metabolites was conducted at the University of Michigan.

Sample preparation

Plasma samples: Samples were stored at -80°C until extraction and were thawed and maintained on wet ice throughout processing steps. Plasma (50 µl aliquot) was extracted by adding 200 µL of extraction solvent (1:1:1 v:v methanol:acetonitrile:acetone containing internal standards listed in Table 3; chemical standards were purchased from Sigma Aldrich or Cambridge Isotope Labs). Samples were vortexed 10 seconds, incubated in ice for 10 minutes, then centrifuged for 10 minutes at 15,000 rcf at 4°C to pellet precipitated proteins. 150 µL

supernatant was transferred to a glass autosampler vial with a low-volume insert and brought to dryness using a nitrogen blower at ambient temperature. Dried samples were reconstituted in 37.5 µL water:methanol (8:2 v:v) for LC-MS analysis. A QC sample was generated by pooling residual supernatant from multiple samples, then drying and reconstituting as above.

Table 3. Internal standard concentrations in extraction solvent

**Internal standard compound**	**Concentration in extraction solvent for plasma (µM, except as noted)**	**Concentration in extraction solvent for tissues (µM, except as noted)**
$^{13}C_3$ lactic acid	50	10
$^{13}C_5$ alpha-ketoglutaric acid	0.5	0.5
$^{13}C_6$ citric acid	5	0.5
$^{13}C_4$ succinic acid	0.5	0.5
$^{13}C_4$ malic acid	0.5	0.5
$^{13}C_6$ fructose 6-phosphate	-	1
$^{13}C_6$ fructose 1,6-bisphosphate	-	0.5
$^{13}C_{10}{}^{15}N_5$ adenosine monophosphate	-	0.5
$^{13}C_{10}{}^{15}N_5$ adenosine triphosphate	-	1
U-^{13}C amino acid mix (Sigma 426199)	10 µg/ml	5 µg/ml
$^{13}C_5$ glutamine	25	5
$^{13}C_6$ cystine	5	1
$^{15}N_2$ asparagine	5	1
$^{15}N_2$ tryptophan	5	1
$^{13}C_6$ glucose	250	2
D_9 L-carnitine	0.38	0.38
D_4 thymine	1	1
^{15}N anthranilic acid	1	1
Gibberelic acid	1	1
Epibrassinolide	1	1
D_3 acetylcarnitine (Cambridge NSK-B)	0.095	0.095
D_3 propionylcarnitine (Cambridge NSK-B)	0.019	0.019
D_3 butyrylcarnitine (Cambridge NSK-B)	0.019	0.019
D_9 isovalerylcarnitine (Cambridge NSK-B)	0.019	0.019
D_3 octanoylcarnitine (Cambridge NSK-B)	0.019	0.019
D_9 myristoylcarnitine (Cambridge NSK-B)	0.019	0.019
D_3 palmitoylcarnitine (Cambridge NSK-B)	0.038	0.038

Tissue samples: Frozen tissue samples were rapidly weighed into pre-tared, pre-chilled Eppendorf tubes and tissue mass was recorded to the nearest 0.1 mg. Extraction solvent was 1:1:1:1 methanol:acetonitrile:acetone:water containing internal standards. To extract samples, chilled extraction solvent was added to a tissue sample at the ratio of 1 ml solvent to 50 mg wet tissue mass. Immediately following solvent addition, the sample was homogenized using a

Branson 450 probe sonicator set to output level 4, 40% duty cycle, for 30 seconds. Tubes were subsequently mixed several times by inversion and then incubated on ice for 10 minutes. Samples were centrifuged at 15,000 rcf for 10 minutes. 300 µL of supernatant was transferred to two autosampler vials with flat-bottom inserts, dried using the nitrogen blower, and stored at -80C until the day of analysis. Samples were reconstituted in 60 µL of 8:2 water:methanol and submitted for LC-MS analysis. A QC sample was generated by pooling residual supernatant from multiple samples, then drying and reconstituting as above.

LC-MS analysis

Non-targeted reverse phase LC-MS: Samples were analyzed on an Agilent 1290 Infinity II / 6545 qTOF MS system with a JetStream electrospray ionization (ESI) source (Agilent Technologies, Santa Clara, California) using a Waters Acquity HSS T3 column, 1.8 µm 2.1 x 100 mm equipped with a matched Vanguard precolumn (Waters Corporation). Mobile phase A was 100% water with 0.1% formic acid and mobile phase B was 100% methanol with 0.025% formic acid. The gradient was as follows: Linear ramp from 0% to 100% B from 0-10 minutes, hold 100% B until 17 minutes, linear return to 0% B from 17 to 17.1 minutes, hold 0% B until 20 minutes. The flow rate was 0.45 ml/min, the column temperature was 55°C, and the injection volume was 5 µL. All solvents and mobile phase additives were LC-MS grade and purchased from Sigma-Aldrich. Each sample was analyzed twice, once in positive and once in negative ion mode MS, scan rate 2 spectra/sec, mass range 50-1200 *m/z*. Source parameters were: drying gas temperature 350°C, drying gas flow rate 10 L/min, nebulizer pressure 30 psig, sheath gas temperature 350°C and flow 11 L/minute, capillary voltage 3500 V, internal reference mass correction enabled. A QC sample run was performed at minimum every tenth injection.

Non-targeted ion pairing LC-MS: Samples were analyzed on an identically-configured LC-MS system using an Agilent Zorbax Extend C18 1.8 µm RRHD column, 2.1 x 150 mm ID, equipped with a matched guard column. Mobile phase A was 97% water, 3% methanol. Mobile phase B was 100% methanol. Both mobile phases contained 15 mM tributylamine and 10 mM acetic acid. Mobile phase C was 100% acetonitrile. Elution was carried out using a linear gradient followed by a multi-step column wash including automated (valve-controlled) backflushing (see Table 4). Column temperature was 35°C and the injection volume was 5 µL. MS acquisition was performed in negative ion mode, scan rate 2 spectra/sec, mass range 50-1200 *m/z*. Source parameters were: drying gas temperature 250°C, drying gas flow rate 13 L/min, nebulizer pressure 35 psig, sheath gas temp 325°C and flow 12 L/min, capillary voltage 3500V, internal reference mass correction enabled. A QC sample run was performed at minimum every tenth injection.

Table 4. IPC-MS gradient program

Time (minutes)	%A	%B	%C	Flow (ml/min)	Flow direction
0	100	0	0	0.25	Normal
2	100	0	0	0.25	Normal
12	1	99	0	0.25	Normal
18	1	99	0	0.25	Normal
18.05	5	0	95	0.25	Backflush

21	5	0	95	0.25	Backflush
21.50	5	0	95	0.80	Backflush
23	5	0	95	0.80	Backflush
23.2	5	0	95	0.60	Backflush
24.00	100	0	0	0.40	Backflush
27.99	100	0	0	0.40	Backflush
28.0	100	0	0	0.40	Normal
29.9	100	0	0	0.40	Normal
30	100	0	0	0.25	Normal

Iterative Data Dependent MS/MS data acquisition (iDDA): To aid in compound identification, iterative MS/MS data was acquired for both reverse phase and ion pairing methods using the pooled sample material. Eight repeated LC-MS/MS runs of the QC sample were performed at three different collision energies (10, 20, and 40) with iterative acquisition enabled. The software excluded precursor ions from MS/MS acquisition within 0.5 minute of their MS/MS acquisition time in prior runs, resulting in deeper MS/MS coverage of lower-abundance precursor ions[81,82].

Data analysis

Feature detection and alignment: Data analysis was performed using a hybrid targeted/untargeted approach. Targeted compound detection and relative quantitation was performed by automatic integration followed by manual review and correction using Profinder v8.0 (Agilent Technologies, Santa Clara, CA.) Non-targeted feature detection was performed using custom scripts that automate operation of the “find by molecular feature” workflow of the Agilent Masshunter Qualitative Analysis (v7) software package. Feature alignment and recursive feature detection were performed using Agilent Mass Profiler Pro (v8.0) and Masshunter Qualitative Analysis (“find by formula” workflow), yielding an aligned table including *m/z*, RT, and peak areas for all features.

Data Cleaning and Degeneracy Removal: A combined feature set was generated by merging untargeted features and named metabolites into a single feature list. Features missing from over 50% of all samples in a batch or over 30% of QC samples were removed prior to subsequent normalization steps. Next, the combined feature set underwent data reduction using Binner[83]. Briefly, Binner first performs RT-based binning, followed by clustering of features by Pearson’s correlation coefficient, and then assigns annotations for isotopes, adducts or in-source fragments by searching for known mass differences between highly correlated features.

Normalization and Quality Control: Data were normalized using a Systematic Error Removal Using Random Forest (SERRF) approach[84], which helps correct for drift in peak intensity over the batch using data from the QC sample runs. When necessary to correct for residual drift, peak area normalization to closest-matching internal standard was also applied to selected compounds. Both SERRF correction and internal standard normalization were implemented in R. Parameters were set to minimize batch effects and other observable drift, as visualized using principal component analysis score plots of the full dataset. Normalization performance was also validated by examining relative standard deviation values for additional QC samples not

included in the drift correction calculations. Quality control reports containing these data were generated for all datasets and uploaded to the MoTrPAC data repository along with raw and processed data.

Compound identification: Metabolites from the targeted analysis workflow were identified with high confidence (MSI level 1)[85] by matching retention time (+/- 0.1 minute), mass (+/- 10 ppm) and isotope profile (peak height and spacing) to authentic standards. MS/MS data corresponding to unidentified features of interest from the untargeted analysis were searched against a spectral library (NIST 2020 MS/MS spectral database or other public spectral databases) to generate putative identifications (MSI level 2) or compound-class level annotations (MSI level 3) as described previously[82].

Reversed phase LC-MS and LC-MS/MS non-targeted lipidomics

Non-targeted profiling of lipid metabolites was conducted at the Georgia Institute of Technology.

Sample preparation

All solvents were LC-MS grade and were purchased from ThermoFisher Scientific. All stable isotope-labeled internal standards (IS) were purchased from Avanti Polar Lipids (Alabaster, Alabama): of PC (15:0-18:1(d7)); PE (15:0-18:1(d7)); PS (15:0-18:1(d7)); PG (15:0-18:1(d7)); PI (15:0-18:1(d7)); LPC (18:1(d7)); LPE (18:1(d7)); Chol Ester (18:1(d7)); DG(15:0-18:1(d7)); TG (15:0-18:1(d7)-15:0); SM (18:1(d9)); and cholesterol (d7). IS were added to the extraction solvent at a final concentration in the 0.1-8 µg/ml range.

Plasma samples: Samples were stored at -80 ^{o}C until extraction. An ice bath was used to thaw and maintain temperature throughout preparation. Plasma samples (25 µL) were vortex mixed with 75 µL of the extraction solvent (isopropanol containing the IS mix listed above) followed by centrifugation for 5 min at 21,100 x G to pellet insoluble material. Supernatant was transferred to autosampler vial and stored at 4 ^{o}C until analysis. An aliquot from each supernatant was combined to create a pooled sample used as a quality control (QC).

Tissue samples: Samples were stored at -80 ^{o}C until extraction. An ice bath was used to thaw the samples. Powdered tissue samples (10 mg) with 400 µl of the extraction solvent (isopropanol containing IS mix listed above) were extracted by freeze-thawing in liquid nitrogen for 1 min followed by sonication in an ice bath for 3 min, repeated three times. Sample were vortex mixed for 5 min in pulsed mode followed by centrifugation for 5 min at 21,100 X G. Supernatant was transferred to autosampler vial and stored at 4 ^{o}C until analysis. An aliquot from each supernatant was combined to create a pooled sample.

Sample blanks and consortium reference samples were prepared for analysis using the same methods.

LC-MS and LC-MS/MS analysis

Lipid LC-MS data were acquired using a Vanquish (ThermoFisher Scientific) chromatograph fitted with a ThermoFisher Scientific Accucore^{TM} C30 column (2.1 × 150 mm, 2.6 µm particle size), coupled to a high-resolution accurate mass Q-Exactive HF Orbitrap mass spectrometer

(ThermoFisher Scientific) for both positive and negative ionization modes. The mobile phases were 40:60 water:acetonitrile with 10 mM ammonium formate and 0.1% formic acid (mobile phase A) and 10:90 acetonitrile:isopropyl alcohol, with 10 mM ammonium formate and 0.1% formic acid (mobile phase B). The column temperature was set to 50°C, the injection volume was 2 µL, and the gradient program is shown in Table 5.

Table 5. LC-MS gradient program

Time (min)	%A	%B	Flow (ml/min)	Curve
0	80	20	0.4	5
1	40	60	0.4	6
5	30	70	0.4	6
8	10	90	0.4	6
8.2	0	100	0.4	6
10.5	0	100	0.4	6
10.7	80	20	0.4	6
12	80	20	0.4	6

For analysis the electrospray ionization source was operated at a vaporizer temperature of 425°C, a spray voltage of 3.0 kV for positive ionization mode and 2.8 kV for negative ionization mode, sheath, auxiliary, and sweep gas flows of 60, 18, and 4 (arbitrary units), respectively, and capillary temperature of 275°C. The instrument acquired full MS data with 240,000 resolution over the 150-2000 *m/z* range. Samples were analyzed in random order with pooled QC injections collected at minimum every tenth injection.

LC-MS/MS experiments were acquired using a data dependent acquisition (DDA) strategy to aid in compound identification. MS^2 spectra were collected with a resolution of 120,000 and the dd-MS^2 were collected at a resolution of 30,000 and an isolation window of 0.4 *m/z* with a loop count of top 7. Stepped normalized collision energies of 10%, 30%, and 50% fragmented selected precursors in the collision cell. Dynamic exclusion was set at 7 seconds and ions with charges greater than 2 were omitted.

Data processing and quality control

Feature detection and alignment: Data processing steps included peak detection, spectral alignment, and gap filling and were performed with Compound Discoverer V3.0 (ThermoFisher Scientific) to yield an aligned feature table containing *m/z*, RT, and relative peak areas.

Data cleaning and degeneracy removal: Compound Discoverer was used to group isotopic peaks as well as adduct ions to simplify the feature table. Detected features were filtered with background and QC filters. Features with abundance lower than 5x the background signal in the sample blanks and that were not present in at least 50% of the QC pooled injections with a coefficient of variance (CV) lower than 30% were removed from the dataset.

Drift correction: Drift correction was performed on each individual feature, where a linear curve was fitted to the pooled QC sample peak areas across the batch and was then used to correct the peak area for that specific feature in the samples.

Quality control: System suitability was assessed prior to the analysis of each batch. A performance baseline for a clean instrument was established before any experiments were conducted. The mass spectrometers were mass calibrated, mass accuracy and mass resolution were checked to be within manufacturer specifications, and signal-to-noise ratios for the suite of IS checked to be at least 75% of the clean baseline values. For LC-MS assays, an IS mix consisting of 12 standards was injected to establish baseline separation parameters for each new column. The performance of the LC gradient was assessed by inspection of the column back pressure trace, which had to be stable within an acceptable range (less than 30% change). Each IS mix component was visually evaluated for chromatographic peak shape, retention time (lower than 0.2 min drift from baseline values) and FWHM lower than 125% of the baseline measurements. The CV of the average signal intensity and CV of the IS (<=15%) in pooled samples were also checked. These pooled QC samples were used to correct for instrument sensitivity drift over the various batches using a procedure similar to that described by the Human Serum Metabolome (HUSERMET) Consortium[86]. To evaluate the quality of the data for the samples themselves, the IS signals across the batch were monitored, PCA modeling for all samples and features before and after drift correction was conducted, and Pearson correlations calculated between each sample and the median of the QC samples.

Compound identification/annotation: Lipid annotations were accomplished based on accurate mass and relative isotopic abundances (to assign elemental formula), retention time (to assign lipid class), and MS^2 fragmentation pattern matching to local spectral databases built from curated experimental data. Lipid nomenclature followed that described by Fahy et al.[87,88]. When possible, features were matched to authentic compounds were identified with MSI level 1. Features that were matched to local databases were annotated with MSI level 2 or compound-class annotations with MSI level 3. Unknown features were assigned MSI level 4. Lipid annotations are highly subject to the available structural information to assign alkyl chain lengths, alkyl chain position, double bond position, and double bond stereochemistry. Annotations reflect the available structural information, which results in a feature with multiple possible lipid structures.

Targeted metabolomics

LC-MS/MS analysis of branched-chain keto acids

Targeted profiling of branched-chain keto acid metabolites was conducted at Duke University. Ten µl of plasma containing isotopically labeled ketoleucine (KIC)-d3, ketoisovalerate (KIV)-$^{13}C_5$ (Cambridge Isotope Laboratories), and 3-methyl-2-oxovalerate (KMV)-d8 (Toronto Research Chemicals, Canada) internal standards were deproteinated with 150 µl of 3M perchloric acid. Two hundred µl of tissue homogenate prepared at 100 mg/ml in 3M perchloric acid were centrifuged at 14,000 x g for 5 minutes. Two hundred µl of 25 M o-phenylenediamine (OPD) in 3M HCl were added to the plasma and tissue supernatants, and the samples were incubated at 80°C for 20 minutes. Keto acids were extracted with ethyl acetate as previously described[89,90]. The extracts were dried under nitrogen, reconstituted in 200 mM ammonium acetate, and analyzed on a Xevo TQ-S triple quadrupole mass spectrometer coupled to an Acquity UPLC

(Waters) controlled by the MassLynx 4.1 operating system. The analytical column (Waters Acquity UPLC BEH C18 Column, 1.7 µm, 2.1 × 50 mm) was used at 30°C. 10 µl of the sample were injected onto the column and eluted at a flow rate of 0.4 ml/min. The gradient consisted of 45% mobile phase A (5 mM ammonium acetate in water) and 55% mobile phase B (methanol) for 2 minutes, followed by a linear gradient to 95% B from 2 to 2.5 minutes, held at 95% B for 0.7 minutes, returned to 45% A, and finally the column was re-equilibrated at initial conditions for 1 minute. The total run time was 4.7 minutes. Mass transitions of *m/z* 203 → 161 (KIC), 206 → 161 (KIC-d3), 189 → 174 (KIV), 194 → 178 (KIV-$^{5}C_{13}$), 203 → 174 (KMV), and 211 → 177 (KMV-d8) were monitored in positive ion mode. The endogenous keto acids were quantified using calibrators prepared by spiking dialyzed fetal bovine serum with authentic keto acids (Sigma-Aldrich).

Flow injection MS/MS analysis of acyl CoAs

Targeted profiling of acyl CoAs was conducted at Duke University. 500 µl of tissue homogenate prepared at 50 mg/ml in isopropanol/0.1 M KH_2PO_4 (1:1) was extracted with an equal volume of acetonitrile and centrifuged at 14,000 x g for 10 min as previously described[91,92]. The supernatants were acidified with 0.25 ml of glacial acetic acid, and acyl CoAs were further purified by solid phase extraction (SPE) using 2-(2-pyridyl) ethyl functionalized silica gel (Sigma-Aldrich) as described[93]. The SPE columns were conditioned with 1 ml of acetonitrile/isopropanol/water/glacial acetic acid (9/3/4/4 : v/v/v/v). Following application and flow through of the supernatant, the SPE columns were washed with 2 ml of acetonitrile/ isopropanol/water/glacial acetic acid (9/3/4/4 : v/v/v/v). Acyl CoAs were then eluted with 2 ml of methanol/250 mM ammonium formate (4/1 : v/v) and analyzed by flow injection MS/MS analysis using positive ion mode on a Xevo TQ-S, triple quadrupole mass spectrometer (Waters), employing methanol/water (80/20, v/v) containing 30 mM ammonium hydroxide as the mobile phase[94]. Spectra were acquired in the multichannel acquisition mode monitoring the neutral loss of 507 amu (phosphoadenosine diphosphate) and scanning from *m/z* 750 to 1100. Heptadecanoyl CoA was employed as an internal standard. The endogenous Acyl CoAs were quantified using calibrators prepared by spiking tissue homogenates with authentic Acyl CoAs (Sigma-Aldrich) having saturated acyl chain lengths C0 - C18. Corrections for the heavy isotope effects, mainly ^{13}C, to the adjacent m+2 spectral peaks in a particular chain length cluster were made empirically by referring to the observed spectra for the analytical standards.

LC-MS/MS analysis of nucleotides

Targeted profiling of nucleotide metabolites was conducted at Duke University. 300 µl of tissue homogenates prepared at 50 mg/ml in 70% methanol were spiked with nine internal standards: $^{13}C^{10}$,$^{15}N^{5}$-adenosine monophosphate, $^{13}C^{10}$,$^{15}N^{5}$-guanosine monophosphate, $^{13}C^{10}$,$^{15}N^{2}$-uridine monophosphate, $^{13}C^{9}$,$^{15}N^{3}$-cytidine monophosphate, $^{13}C^{10}$-guanosine triphosphate, $^{13}C^{10}$-uridine triphosphate, $^{13}C^{9}$-cytidine triphosphate, $^{13}C^{10}$-adenosine triphosphate, and nicotinamide-1, N^{6}-ethenoadenine dinucleotide (eNAD) (Sigma-Aldrich). Nucleotides were extracted using an equal volume of hexane as described by Cordell et al. and Gooding et al.[95,96]. The samples were vortexed and centrifuged at 14,000 x g for 5 minutes. The bottom layer was collected and centrifuged again. Chromatographic separations and MS analysis of the supernatants were

performed using an Acquity UPLC system (Waters) coupled to a Xevo TQ-XS quadrupole mass spectrometer and (Waters). The analytical column (Chromolith FastGradient RP-18e 50-2mm column, EMD Millipore, Billerica, MA, USA) was maintained at 40°C. The injection volume was 2 μL. Nucleotides were separated using a mobile phase A consisting of 95% water, 5% methanol, and 5 mM dimethylhexylamine adjusted to pH 7.5 with acetic acid and a mobile phase B consisting of 20% water, 80% methanol, and 10 mM dimethylhexylamine. Flow rate was set to 0.3 ml/min. The 22-minute gradient (t=0, %B=0; t=1.2, %B=0; t=22, %B=40) was followed by a 3-minute wash and 7-minute equilibration. Nucleotides were detected in the negative ion MRM mode based on characteristic fragmentation reactions. The endogenous nucleotides were quantified using calibrators prepared by spiking tissue homogenates with authentic nucleotides (Sigma-Aldrich).

LC-MS/MS analysis of amino acids and amino metabolites

Targeted profiling of amino acids and amino metabolites was conducted at the Mayo Clinic by LC-MS as previously described[97,98]. Briefly, either 20 μL of plasma samples or 5 mg of tissue homogenates were spiked with an internal standard solution consisting of isotopically labeled amino acids (U-$^{13}C_4$ L-aspartic acid,U-$^{13}C_3$ alanine, U-$^{13}C_4$ L-threonine, U-^{13}C L-proline, U-$^{13}C_6$ tyrosine, U-$^{13}C_5$ valine, U-$^{13}C_6$ leucine, U-$^{13}C_6$ phenylalanine, U-$^{13}C_3$ serine, U-$^{13}C_5$ glutamine, U-$^{13}C_2$glycine, U-$^{13}C_5$ glutamate, U-$^{13}C_6$,$^{15}N_2$ lysine, U-$^{13}C_5$,^{15}N methionine, 1,1U-$^{13}C_2$ homocysteine, U-$^{13}C_6$ arginine, U-$^{13}C_5$ ornithine, $^{13}C_4$ asparagine, $^{13}C_2$ ethanolamine, d3 sarcosine, d6 4-aminobutyric acid) . The supernatant was immediately derivatized with 6-aminoquinolyl-N-hydroxysuccinimidyl carbamate using a MassTrak kit (Waters). A 10-point calibration standard curve underwent a similar derivatization procedure after the addition of internal standards. Both derivatized standards and samples were analyzed on a Quantum Ultra triple quadrupole mass spectrometer (ThermoFischer) coupled with an Acquity liquid chromatography system (Waters). The LC gradient is shown in Table 6. Buffer A was 0.1% formic acid, 1% Acetonitrile, 99% Water and Buffer B was 1% water, 99% Acetonitrile. Data acquisition was conducted by using selected ion monitoring (SRM) in positive ion mode using transitions shown in Table 7. Concentrations of 42 analytes of each unknown were calculated against their respective calibration curves.

Table 6. LC-MS gradient program

Time	%A	%B	Flow (mL/min)
initial	99.9	0.1	0.400
1.00	99.9	0.1	0.400
2.00	99.9	0.1	0.400
5.50	98.1	1.9	0.400
6.50	98.0	2.0	0.400
10.00	97.5	2.4	0.400
12.00	96.0	4.0	0.400
20.00	88.0	12.0	0.400
27.00	86.5	13.5	0.400
31.00	80.0	20.0	0.400

32.00	2.0	98.0	0.500
35.00	2.0	98.0	0.500
35.50	99.9	0.1	0.400
39.00	99.9	0.1	0.400

Table 7. SRM transitions

**Compound**	**Abbrev.**	**Prec ion scan 8-8-13**	**Prod Ion**	**I.S.**
Ammonia_188	ammon	188	171	u-arg
Histidine_163	his	164	171	u-arg
Hydroxyproline_302	hyPro	302	171	u-arg
1-Methylhistidine_340	1MH	340	171	u-arg
3-Methylhistidine_340	3MH	340	171	u-arg
Asparagine_303	Asn	303	171	u-asn
**U-$^{13}C_4$-Asparagine_307**	**u-Asn**	**307**	171	
Phosphoethanolamine_312	PEA	312	171	u-arg
Arginine_345	arg	345	171	u-arg
**U-$^{13}C_6$-Arginine_351**	**u-arg**	**351**	171	
Carnosine_199	carn	199	171	u-gly
Taurine_296	tau	296	171	u-gly
Anserine_206	ans	206	171	U-$^{13}C_3$-ser
Serine_276	ser	276	171	U-$^{13}C_3$-ser
**U-$^{13}C_3$-Serine_279**	**u-ser**	**279**	171	-
Glutamine_317	gln	317	171	u-gln
**U-$^{13}C_5$-Glutamine_322**	**u-gln**	**322**	171	-
Ethanolamine_232	EA	232	171	u-EA
**U-$^{13}C_2$-Ethanolamine_234**	**u-EA**	**234**	171	
Glycine_246	gly	246	171	u-gly
**U-$^{13}C_2$-Glycine_249**	**u-gly**	**249**	171	-
Aspartic Acid_304	asp	304	171	u-asp
**U-$^{13}C_4$-Aspartic acid_308**	**u-asp**	**308**	171	-
Sarcosine_260	sar	260	171	d3-sar
**d3-Sarcosine_263**	**d3-sar**	**263**	171	
Citrulline_346	cit	346	171	u-glu
Glutamic Acid_318	glu	318	171	u-glu
**U-$^{13}C_5$-glutamic acid_323**	**u-glu**	**323**	171	-
beta-Alanine_260	b-ala	260	171	u-glu
Threonine_290	thr	290	171	u-thr
**U-$^{13}C_4$-threonine_294**	**u-thr**	**294**	171	-
Alanine_260	ala	260	171	u-ala
U-$^{13}C_3$-alanine_263	**u-ala**	**263**	171	-
gamma-Amino-N-butyric-acid_274	gaba	274	171	d6-gaba
**d6-gamma-Amino-N-butyric-acid_280**	**d6-gaba**	**280**	171	

alpha-Aminoadipic-acid_332	aada	332	171	u-ala
beta-Aminoisobutyric-acid_274	baib	274	171	u-ala
Proline_286	pro	286	171	u-pro
**u-pro_291**	**u-pro**	**291**	171	-
Hydroxylysine 1_252	HL 1	252	171	u-pro
Hydroxylysine 2_252	HL 2	252	171	u-pro
alpha-Amino-N-butyric-acid_274	aaba	274	171	u-val
Ornithine_237	orn	237	171	u-orn
**$U^{13}C_5$-Ornithine_239**	**u-orn**	**239**	171	-
Cystathionine 1_282	cyst1	282	171	u-$^{15}N_2$-Lys
dervitizing agent_315	deriv agent	315	171	u-tyr
Cystathionine 2_282	cyst2	282	171	u-$^{15}N_2$-Lys
Lysine_244	lys	244	171	u-$^{15}N_2$-Lys
**U^{13}-C_6, $^{15}N_2$-Lysine_248**	**u-$^{15}N_2$-Lys**	**248**	171	-
Cystine_291	cys	291	171	u-tyr
Tyrosine_352	tyr	352	171	u-tyr
**U-$^{13}C_6$-Tyrosine_358**	**u-tyr**	**358**	171	-
Methionine_320	met	320	171	u-$^{15}N_2$-met
**U^{13}-C_5, $^{15}N_2$-met_326**	**u-$^{15}N_2$-met**	**326**	171	-
Valine_288	val	288	171	u-val
**$U^{13}C_5$-Valine_293**	**u-val**	**293**	171	-
Isoleucine_302	ileu	302	171	u-leu
allo-Isoleucine_302	aileu	302	171	u-leu
Homocystine_306	hcys	305	171	$^{13}C_2$-hys
**1,1-$^{13}C_2$-Homocystine_308**	**1,1-$^{13}C_2$-hcys**	**306**	171	-
Leucine_302	leu	302	171	u-leu
**$U^{13}C_6$-Leucine_302**	**u-leu**	**308**	171	-
Phenylalanine_335	phe	335	171	u-phe
**$U^{13}C_6$-Phenylalanine_342**	**u-phe**	**342**	171	-
Tryptophan_375	trp	375	171	u-phe

GC-MS analysis of tricarboxylic acid cycle (TCA) metabolites

Targeted profiling of TCA metabolites was conducted at the Mayo Clinic by gas chromatography mass spectrometry (GC-MS) as previously described[99,100], with a few modifications. Briefly, 5 mg of tissue were homogenized in 1X PBS on an Omni bead ruptor (Omni International, Kennesaw, GA) prior to adding 20 µl of internal solution containing U-^{13}C labeled analytes ($^{13}C_3$ sodium lactate, $^{13}C_4$ succinic acid, $^{13}C_4$fumaric acid, $^{13}C_4$ alpha ketoglutaric acid, $^{13}C_4$ malic acid, $^{13}C_4$ aspartic acid, $^{13}C_5$ 2-hydroxyglutaric acid, $^{13}C_5$glutamic acid, $^{13}C_6$ citric acid, $^{13}C_2$,^{15}N glycine, $^{13}C_2$ sodium pyruvate). For plasma, 50 µl were used. The proteins were removed by adding 300 µl of chilled methanol and acetonitrile solution to the sample mixture. After drying the supernatant in the speedvac, the sample was derivatized with ethoxime and then with MtBSTFA + 1% tBDMCS

before it was analyzed on an Agilent 5977B GC/MS (Santa Clara, California) under single ion monitoring conditions using electron ionization. Concentrations of lactic acid (*m/z* 261.2), fumaric acid (*m/z* 287.1), succinic acid (*m/z* 289.1), ketoglutaric acid (*m/z*360.2), malic acid (*m/z* 419.3), aspartic acid (*m/z* 418.2), 2-hydroxyglutaratic acid (*m/z* 433.2), cis aconitic acid (*m/z*459.3), citric acid (*m/z* 591.4), and isocitric acid (*m/z* 591.4), glutamic acid (*m/z* 432.4) were measured against 7-point calibration curves that underwent the same derivatization procedure using ethoxyamine and MtBSTFA. The injection volume was 1uL, and the flow rate was 1mL/min. Table 8 shows the temperature program. Agilent MassHunter was used for data processing.

Table 8. GC temperature gradient program

	Rate (°C/min)	Value (°C)	Hold Time (min)	Run Time (min)
**Initial**		120	0.5	0.5
**Ramp 1**	25	188	0	3.22
**Ramp 2**	7	275	0	15.649
**Ramp 3**	35	320	1.066	18

LC-MS/MS analysis of ceramides

Targeted profiling of ceramides was conducted at the Mayo Clinic. Plasma and tissue ceramides, sphinganine, sphingosine, sphingosine-1-phosphate (S1P) were measured by previously described methods[101,102]. Briefly, a 25 µl aliquot of plasma or 5 mg tissue homogenate was spiked with an internal standard mixture prior to undergoing extraction. Data acquisition was conducted in SRM mode after chromatographic separation in electrospray ionization in positive mode on Thermo Quantiva mass spectrometer coupled to a Waters Acquity UPLC system. Concentrations of each analyte were calculated against their respective calibration curves. Coefficients of variation for a healthy plasma control analyzed with each batch of 40 samples over a one-month period were 6.3%, 6.2%, 3.1%, 5.0%, 5.7%, 3.2%, 4.9% and 3.3% for sphingosine, sphingosine-1-phosphate, C16:0-ceramides, C18:0-ceramides, C20:0-ceramide, C22:0-ceramide, C24:1-ceramide and C24:0-ceramide, respectively. Table 9 shows the gradient table where Buffer A consisted of 2mM Ammonium formate, 0.1% formic acid, 1% MeOH, 99% water, and Buffer B consisted of 1mM Ammonium formate, 0.1% formic acid, 1% water, 99% MeOH. SRM transitions are listed in Table 10.

Table 9. Gradient program

Time	%A	%B	Flow (mL/min)
initial	20.0	80.0	0.400
1.50	10.0	90.0	0.400
2.50	10.0	90.0	0.400
10.00	10.0	90.0	0.400
12.00	1.0	99.0	0.400
16.00	1.0	99.0	0.400
16.20	20.0	80.0	0.400
20.00	20.0	80.0	0.400

Table 10. SRM transitions

Analyte	parent (m/z)	product (m/z)
Sph	300.30	282.30
**d7-Sph**	**307.34**	**289.27**
SPA	302.30	284.30
**d7-SPA**	**309.35**	**291.30**
S1P	380.30	264.30
**d7-S1P**	**387.29**	**271.30**
C8-Ceramide	426.40	264.25
C14-Ceramide	510.70	264.25
C16-Ceramide	538.70	264.25
**d7-C16-Ceramide**	**545.53**	**271.28**
C18:1-Ceramide	564.70	264.25
C18-Ceramide	566.60	264.25
**d7-C18-Ceramide**	**573.58**	**271.28**
C20-Ceramide	594.80	264.25
C22-Ceramide	622.60	264.25
C24:1-Ceramide	648.80	264.25
**d7-C24:1-Ceramide**	**655.65**	**271.27**
C24-Ceramide	650.80	264.25
**d7-C24-Ceramide**	**657.68**	**271.27**

LC-MS/MS analysis of acylcarnitines

Targeted profiling of acylcarnitines was conducted at the Mayo Clinic. Acylcarnitines (specifically C0-C18:1) were measured by LC-MS using established methods[100,103]. Briefly, 25 µl of plasma or 5 mg homogenized tissue were spiked with an IS consisting of isotopically-labeled acylcarnitines. The samples were then extracted with cold MeOH:DCM (1:1) followed by centrifugation at 12,000 g for 10 minutes. The supernatant was transferred to another vial, dried down and reconstituted in mobile phase. A calibration curve was built from a purchased acyl carnitine mix aliquoted at various concentrations, and spiked with the same IS as the samples. The samples and calibration standards were analyzed on a triple quadrupole mass spectrometer coupled with an UHPLC system. Separate gradient programs were used for C0-C8 species (Table 11) and C10-C18 species (Table 12), where Buffer A consisted of 2mM Ammonium formate, 0.1% formic acid, 1% MeOH, 99% water, and Buffer B consisted of 1mM Ammonium formate, 0.1% formic acid, 1% water, 99% MeOH. Data acquisition was conducted in SRM mode (Table 13). Analyte concentrations of each unknown were calculated against their perspective standard curves.

Table 11. C0-C8 gradient program

Time	%A	%B	Flow (mL/min)
initial	99	1	0.35
0.5	99	1	0.35
6.5	20	80	0.35

10	1	99	0.35
12	1	99	0.35
13	99	1	0.35
16	99	1	0.35

Table 12. C10-C18 gradient program

**Time**	**%A**	**%B**	**Flow (mL/min)**
initial	99	1	0.35
0.5	99	1	0.35
6.5	87	13	0.35
8	80	20	0.35
10.5	1	99	0.35
13.5	1	99	0.35
14	99	1	0.35
17	99	1	0.35

Table 13. SRM transitions

**analyte**	**parent m/z**	**product m/z**
Carnitine	162.10	85.02
**d9-carnitine**	**171.11**	85.02
acetylcarnitine	204.12	85.02
**d3-acetylcarnitine**	**207.12**	85.02
propionylcarnitine	218.14	85.02
**d3-propionylcarnitine**	**221.14**	85.02
butyryl/isobutyryl carnitine	232.15	85.02
**d3-butyryl carnitine**	**235.39**	85.02
isovaleryl/valeryl carnitine	246.17	85.02
**d9-isovaleryl carnitine**	**255.17**	85.02
octanoylcarnitine	288.22	85.02
**d3-octanoylcarnitine**	**291.36**	85.02
decanoylcarnitine	316.20	85.02
**d3-decanoylcarnitine**	**319.20**	85.02
Lauroylcarnitine	344.28	85.02
**d9-Lauroylcarnitine**	**353.56**	85.02
myristoylcarnitine	372.36	85.02
**d9-myristoylcarnitine**	**381.36**	85.02
palmitoylcarnitine	400.39	85.02
**d3-palmitoylcarnitine**	**403.37**	85.02
**C17-carn**	**414.39**	85.02
oleoylcarnitine	426.39	85.02
stearoylcarnitine	428.39	85.02

Targeted lipidomics of low-level lipids

Sample preparation

Targeted profiling of lipids was conducted at Emory University using previously published methods[104,105]. Powdered tissue samples (10 mg) were homogenized in 100 µl PBS with Bead Ruptor (Omni International, Kennesaw, GA). Homogenized samples were diluted with 300 µL 20% methanol and spiked with 1% BHT solution to a final BHT concentration of 0.1% and pH of 3.0 by acetic acid addition. Samples were then centrifuged (10 minutes, 14,000 rpm), and the supernatants were transferred to 96-well plates for further extraction. The supernatants were loaded to Isolute C18 SPE columns (conditioned with 1000 µL ethyl acetate and 1000 µL 5% methanol). The SPE columns were then washed with 800 µL water and 800 µL hexane. The oxylipins were eluted with 400 µl methyl formate. Automated solid phase extraction (SPE) was conducted with a Biotage Extrahera (Uppsala, Sweden). The eluate was dried with nitrogen and then reconstituted with 200 µL methanol prior to LC-MS analysis. Sample blanks, pooled extract samples used as quality controls (QC), and consortium reference samples, were prepared for analysis using the same methods. The external standards consisted of prostaglandin E2 ethanolamide, oleoyl ethanolamide, palmitoyl ethanolamide, arachidonoyl ethanolamide, docosahexaenoyl ethanolamide, linoleoyl ethanolamide, stearoyl ethanolamide, oxy-arachidonoyl ethanolamide, 2-arachidonyl glycerol, docosatetraenoyl ethanolamide, α-linolenoyl ethanolamide, oleamide, dihomo-γ-linolenoyl ethanolamide, decosanoyl ethanolamide, 9,10 DiHOME, prostaglandin E2-1-glyceryl ester, 20-HETE, 9-HETE, 14,15 DiHET, 5(S)-HETE, 12(R)-HETE, 11(12)-DiHET, 5,6-DiHET, thromboxane B2, 12(13)-EpOME, 13 HODE, prostaglandin F2α, 14(15)-EET, 8(9)-EET, 11(12)-EET, leukotriene B4, 8(9)-DiHET, 13-OxoODE, 13(S)-HpODE, 9(S)-HpODE, 9(S)-HODE, resolvin D3, resolvin E1, resolvin D1, resolvin D2, 9(S)HOTrE, 13(S)HOTrE, 8-iso Progstaglandin F2α. All external standards were purchased from Cayman Chemical (Ann Arbor, Michigan) at a final concentration in the range 0.01-20 µg/ml.

Data collection

Targeted oxylipin and endocannabinoid data were acquired using an ExionLC/Qtrap5500 (SCIEX, Waltham, MA) LC-MS system. For chromatography, a ThermoFisher Scientific AccucoreTM C18 column (100 mm × 4.6, 2.6 µm particle size) was used. The mass spectrometry data acquisition was conducted in both positive and negative ion modes. The mobile phases were water with 10 mM ammonium acetate (mobile phase A), and acetonitrile with 10 mM ammonium acetate (mobile phase B). The chromatographic method used the following gradient program shown in Table 14. The column temperature was set to 50°C, and the injection volume was 10 µL with negative ion mode and 2 µL with positive ion mode. For mass spectrometry analysis, the heated electrospray ionization source was operated at a vaporizer temperature of 650°C, a spray voltage of 5.5 kV for positive ion mode and 4.5 kV for negative ion mode, curtain gas, ion source gas 1, ion source gas 2 were 20, 60 and 50, respectively. The declustering potential, entrance potential, collision energy, and collision cell exit potential were 200, 10, 40, and 10 for the negative ion mode, respectively and 90, 10, 47

and 18, respectively, for positive ion mode. MRM transitions are shown in Table 15 (negative mode) and Table 16 (positive mode).

Table 14. Targeted lipidomics LC-MS gradient program

Time	%A	%B	Flow (mL/min)
0	90	10	0.5
0.5	90	10	0.5
1	50	50	0.5
2	50	50	0.5
2.1	25	75	0.5
5	25	75	0.5
7	15	85	0.5
12	15	85	0.5
12.1	90	10	0.5
18	90	10	0.5

Table 15. Targeted lipidomics negative mode MRM transitions

analyte	parent m/z	product m/z
9,10 DIHOME	313.2	201.0
PGE E2	351.2	315.1
20- HETE	319.2	289.2
9-HETE	319.2	167.2
14,15 DHET	337.2	207.0
5 HETE	319.2	115.1
12 R-HETE	319.2	179.1
11,12-DHET	337.2	167.1
8,9-DHET	337.2	127.2
5,6 EET	319.2	191.1
5,6-DHET	337.2	71.0
TXB2	369.2	169.1
PGF2A	353.2	193.3
14(15)-EET / 15-HETE	319.2	219.0
LTB4	335.2	195.1
8(9)-EET	319.2	69.2
11(12)-EET	319.2	167.1
Resolvin D1	375.3	215.1
Resolvin D2	375.3	215.2
Resolvin D3	375.3	147.1
Resolvin E1	349	109
Resolvin E1	349	161
12(13)-EpOME	295.2	195.1
13 HODE	295.2	195.1

13-OxoODE (13-KODE)	293.2	113.1
13(S)-HpODE	311.2	113.1
13(S)HOTrE	293.2	195.1
9(S)-HODE	295.2	277.6
9(S)-HODE	295.2	171.3
9(S)-HpODE	311.2	201.0
9(S)-HpODE	311.2	185.1
9(S)HOTrE	293.2	185.1
9(S)HOTrE	293.2	171.1
8-iso Progstaglandin F2α	353.2	255
8-iso Progstaglandin F2α	353.2	219.2
8-iso Progstaglandin F2α	353.2	193.1

Table 16. Targeted lipidomics positive mode MRM transitions

analyte	parent m/z	product m/z
PGE2 Ethanolamide (PGE2-EA)_271.3	396.5	271.3
PGE2 Ethanolamide (PGE2-EA)_358.3	396.5	358.3
PGE2 Ethanolamide (PGE2-EA)_376.3	396.5	376.3
Oleoyl Ethanolamide (OEA)	326.4	62.1
Palmitoyl Ethanolamide	300.4	62.1
ARA-Ethanolamide (AEA)	348.4	62.1
Docosahexaenoyl Ethanolamide (DHEA)	372.4	62.1
Linoleoyl Ethanolamide (LEA)	324.4	62.1
Stearoyl Ethanolamide (ceramid)	328.4	62.1
oxy-Arachidonoyl Ethanolamide (oxy-AEA)	364	76
2-Arachidonoyl Glycerol (2AG) trans1	379.4	135
2-Arachidonoyl Glycerol (2AG) trans2	379.4	161
Docosatetraenoyl Ethanolamide (DEA)	376.59	62.1
Alpha-Linolenoyl Ethanolamide (ALEA)	322.4	62.1
Oleamide	282.5	247.4
Dihomo-Gamma-Linolenoyl Ethanolamide	350.4	62.1
Docosanoyl Ethanolamide	384.5	62.1

Data processing and quality control

Sciex OS (AB SCIEX, Version 1.6.1) was used to process the raw LC-MS data. Standard curves were built for each oxylipin/endocannabinoid and calibrated against external standards, i.e, all concentration points should be in the linear portion of the curve with an R-squared value no less than 0.9. Additionally, features with a high coefficient of variation (CV), defined as CV > 150%, in pooled QCs were removed from the dataset. Pearson correlation among the QCs for each tissue type were calculated with the *Hmisc* R library, and figures documented in QC report were plotted with the *corrplot* R library[106,107].

Metabolomics data processing and normalization

Metabolomics sample pre-processing was performed by each Chemical Analysis Site (CAS) as described above for each platform. These datasets were separated into two data types: “named”, for chemical compounds confidently identified, and “unnamed”, for compounds with specific chemical properties but without a standard chemical name. Only named metabolites were included in downstream analyses. The resulting named datasets were the input for the data processing and normalization procedures described in this section. Table 17 provides a summary of features measured by each platform, and the tissues and samples analyzed by each platform are presented in Extended Data Fig. 1e. The large number of features identified by reversed-phase positive untargeted lipidomics (lrppos) is due to a large number of multi-peak isomers.

For both untargeted and targeted datasets, measurements were $\log_2$-transformed, negative values were converted to missing values, and features with >20% missing values were removed. For untargeted datasets and targeted datasets with more than 12 features, remaining missing values were imputed using K-nearest neighbors (k=10 samples). Missing values in targeted datasets with 12 or fewer features were not imputed. For outlier identification in each dataset, we calculated each sample's median correlation value against the other N-1 samples and selected a threshold to designate outliers as those with below-threshold median correlation values. All outliers were reviewed by Metabolomics CAS, and only 21 confirmed technical outliers in the untargeted datasets were removed.

To normalize untargeted datasets, we median-centered samples if neither sample medians nor upper quartiles were significantly associated with sex or sex-stratified training group (Kruskal-Wallis p-value < 0.01). Targeted datasets were not normalized as they were quantified using absolute concentrations.

A total of 1116 metabolites were measured by two or more platforms. We retained all features with repeated measurements and integrated their differential analysis results using meta-regression (see “Meta-analysis of metabolomics results”).

Table 17. Description of metabolomics platforms.

ASSAY	DESCRIPTION	SITE	TARGETED	N TISSUES	MAX N NAMED FEATURES
hilicpos	HILIC positive	Broad	0	19	306
lrppos	Untargeted lipidomics, reversed-phase positive	GTech	0	9	1341
rppos	Reversed-phase positive	UMich	0	9	211
ionpneg	Ion pairing negative	UMich	0	9	80
rpneg	Reversed-phase negative	UMich	0	9	160

ASSAY	DESCRIPTION	SITE	TARGETED	N TISSUES	MAX N NAMED FEATURES
hilicpos	HILIC positive	Broad	0	19	306
lrppos	Untargeted lipidomics, reversed-phase positive	GTech	0	9	1341
rppos	Reversed-phase positive	UMich	0	9	211
lrpneg	Untargeted lipidomics, reversed-phase negative	GTech	0	9	523
amines	Amines	Mayo	1	9	39
tca	TCA cycle	Mayo	1	9	12
etamidpos	Ethanolamides	Emory	1	9	10
oxylipneg	Oxylipins	Emory	1	9	27
ka	Ketoacids	Duke	1	5	3
acoa	Acyl-CoA	Duke	1	4	67
nuc	Nucleotides	Duke	1	4	36

Multiplexed bead-based immunoassays

Protein extraction

Tissue lysates for the immunoassays were prepared at Stanford University. All 30 samples per tissue (15+15 for testes and ovaries, 3 samples per sex per time point, 5 time points) were processed in a single batch. Upon retrieval from storage at -80°C, tissue samples were kept on dry ice until addition of a lysis buffer. Cold lysis buffer (25mM Tris-HCl, pH 7.5; 100mM NaCl; 0.01% Triton-X 100; 0.0025% NP-40) was supplemented with Halt protease inhibitor cocktail (Thermo Fisher # 78429) to 1x just prior to use. 550 µl of chilled lysis buffer were added to each tissue sample; the tube was inverted several times and placed on ice. To facilitate lysis, each sample was sonicated using a Branson Sonifier 250 with 3 1-second bursts at an output setting of 1 and constant duty cycle. Samples were rocked at 4°C for 1 hour. Sample tubes were then centrifuged at max speed (>20,000 xg) at 4°C for 10 minutes to pellet debris. The cleared supernatant from each sample was then transferred into an Eppendorf Protein Lo-Bind 1.5-ml tube. Protein concentrations of lysates were measured in duplicates using the BCA Assay (Thermo Fisher # 23225). A Tecan Infinite M1000 plate reader was used to read absorbance at 562 nm. Microsoft Excel was used to generate a standard curve based on the background-subtracted absorbance of provided BSA protein standards, and this curve was used to extrapolate the protein concentrations in each tissue sample lysate. All samples for a given tissue were then normalized to the protein concentration of the lowest sample through addition

of an appropriate amount of lysis buffer. Lysates from each tissue were then plated into 2 replicate 96-well master plates (Greiner # 651201). Plates were sealed with PCR seals and stored at -80°C prior to analysis.

Targeted multiplexed bead-based immunoassays

The immunoassays were performed by the Stanford Human Immune Monitoring Center (HIMC). The rat tissue lysates were kept at -80°C until used for immunoassays. Levels of 54 cytokines were measured in rat samples using five Luminex panels: MILLIPLEX MAP Rat Cytokine/Chemokine Magnetic Bead Panel (Millipore, RECYTMAG-65K); MILLIPLEX MAP Rat Myokine Magnetic Bead Panel (Millipore, RMYOMAG-88K); MILLIPLEX MAP Rat Metabolic Hormone Magnetic Bead Panel (Millipore, RMHMAG-84K); MILLIPLEX MAP Rat Putuitary Magnetic Bead Panel (Millipore, RPTMAG-86K); MILLIPLEX MAP Rat Adipokine Magnetic Bead Panel (Millipore, RADPKMAG-80K). All samples from the same tissue were analyzed on the same plate to avoid plate-to-plate variability. Therefore, batch correction within a tissue was not possible or necessary. Custom Assay CHEX control beads (Radix BioSolutions, Georgetown, Texas) were added to all wells to monitor instrument performance, application of the detection antibody, application of the fluorescent reporter, and nonspecific binding (CHEX1, CHEX2, CHEX3, and CHEX4, respectively)[108]. Samples were mixed with antibody-linked magnetic beads on a 96-well plate and incubated overnight at 4°C with shaking. Cold and room-temperature incubation steps were performed on an orbital shaker at 500-600 rpm. Plates were washed twice with wash buffer in a Biotek ELx405 washer. Following one-hour incubation at room temperature with biotinylated detection antibody, streptavidin-PE was added for 30 minutes with shaking. Plates were washed as described above and PBS was added to wells for reading in the Luminex FlexMap3D Instrument with a lower bound of 50 beads per sample per cytokine. Reports were generated using Masterplex QT/Mirai Bio/Hitachi software and exported as csv files. Samples were measured in singlets.

Data preprocessing

Raw mean fluorescence intensities (MFI) were $\log_2$-transformed, and measurements for analytes with fewer than 20 beads in a well were removed. Log_2-MFI for analytes measured by multiple panels were correlated to determine reproducibility. TNFA measurements were close to background levels in the Rat Metabolic Hormone Panel likely due to dilutions, while TNFA measured in Rat Cytokine/Chemokine Magnetic Bead Panel showed a dynamic range of signal intensities. Thus, TNFA measurements from the Rat Metabolic Hormone Panel were removed. For remaining measurements in each panel and tissue, samples with more than 50% missing values (i.e., due to low bead count) were removed; features with at least two missing values for a single experimental group (e.g., males trained for 2 weeks) were removed (this affected four colon analytes and one spleen analyte, all in the Rat Cytokine/Chemokine Panel). Remaining missing values were imputed with k-nearest neighbors (k=5 features). This was the version of the data used for visualization (e.g., principal component analysis). Within each panel, tissue, and analyte, we calculated the mean and standard deviation and removed outlying measurements more than 4 standard deviations away from the mean. This version of the data with extreme feature-specific outliers removed was used for differential analysis.

STATISTICAL ANALYSES

Outlier identification

Outlier identification is described in “Metabolomics data processing and normalization” for metabolomics datasets and “Multiplexed bead-based immunoassays” for immunoassay data. Otherwise, outliers were identified through a combination of principal component analysis of normalized datasets and examination of assay-specific quality control metric scores. For all proteomics, transcriptomics, RRBS, and ATAC-seq datasets, we first examined the top three principal components of each tissue separately. Samples were flagged if they fell outside of three times the interquartile range for at least one of the first three principal components. Refined methods were used for remaining data types to identify extreme outliers for exclusion from differential analysis. All identified outliers were manually inspected before removal from the final dataset used for downstream analysis.

For transcriptomics datasets, principal component analysis (PCA) was run separately on the top 1000 most variable genes in each tissue, using the TMM-normalized data described above. Outliers were defined as samples outside of five times the interquartile range for any principal component that explained at least five percent of variance in the data. A total of 16 samples were removed.

For ATAC-seq datasets, PCA was run separately on the top 10,000 most variable features in each tissue and sex, using the normalized data described above. Outliers were defined as samples outside of three times the interquartile range for any principal component that explained at least 7.5% of variance in the data. A total of 11 samples were removed.

Five RRBS samples from the kidney dataset were excluded based on their sequencing quality scores (e.g., having a low total number of reads). Two additional samples, one from lung and one from hippocampus, were removed as they fell outside of five times the interquartile range of at least one of the three first principal components.

For proteomics datasets, the following samples were removed as they were flagged by PCA and also showed large differences in median TMT reporter ion intensity: two gastrocnemius samples from the global proteome and phosphoproteome datasets; one heart sample from global proteome, phosphoproteome, and acetylome datasets; and one additional heart sample only from the ubiquitylome dataset. As detailed in the experimental methods, all acetylome samples labeled with the TMT channel 130C were suspected to be failed reactions caused by the reagent lot and excluded from downstream analysis.

1-week and 2-week female vena cava samples were excluded from all analyses due to apparent brown adipose tissue contamination, as indicated by gene expression levels of *Ucp1*, a brown adipose marker, and confirmation that brown adipose contamination was sometimes visible during vena cava dissection.

Differential analysis

All differential analyses were performed separately in each tissue and ome dataset (e.g. heart RNA-Seq). Males and females in a dataset were analyzed separately as we often observed significantly different residual variances between males and females when both sexes were fitted together. Limma with empirical Bayes variance shrinkage was used to perform differential analyses for all proteomics, metabolomics, and ATAC-seq data[109]; the edgeR pipeline for methylation analysis was used for RRBS[110]; DESeq2 was used for RNA-Seq[68]; base R linear models, *lmtest*, and *multcomp* were used for the immunoassays[111,112]. For all proteomics, ATAC-seq, immunoassays, and RRBS, the input for differential analysis was the normalized data described above. For RNA-Seq, the input was filtered raw counts, in accordance with the DESeq2 workflow. For targeted metabolomics, KNN-imputed log_2-transformed data were used for datasets with more than 12 features; otherwise, log_2-transformed data were used. For untargeted metabolomics, log_2 KNN-imputed data were used; the sample data were median-centered if neither sample medians nor upper quartiles were significantly associated with sex or sex-stratified training group (Kruskal-Wallis p-value < 0.01).

In order to select analytes that changed over the training time course, we performed F-tests (limma, *edgeR::glmQLFTest*) or likelihood ratio tests (*DESeq2::nbinomLRT*, *lrtest*) to compare a full model with ome-specific technical covariates and training group as a factor variable (i.e. sedentary, 1 week, 2 weeks, 4 weeks, 8 weeks) against a reduced model with only technical covariates. For the immunoassays, a training group was excluded from the model if there were fewer than two non-missing values. For each feature, male- and female-specific p-values were combined using Fisher's sum of logs meta-analysis to provide a single p-value, referred to as the *training p-value*. To account for false discovery rate across all statistical tests, the training p-values were adjusted across all datasets within each ome using Independent Hypothesis Weighting (IHW) with tissue as a covariate[113]. Training-differential features were selected at 5% IHW FDR.

Given the regression model of each analyte computed as explained above, we used the contrasts of each training time point versus the sex-matched sedentary controls to calculate time- and sex-specific effect sizes, their variance, and their p-values (e.g., using linear F-tests), referred to as the *timewise summary statistics*. Specifically, for limma models we used *limma::contrasts.fit* and *limma::eBayes*, for DESeq2 models we used *DESeq2::DESeq*, for edgeR models we used *edgeR::glmQLFTest*, and for the immunoassays we used *aov()* and *multcomp::glht()*. For the immunoassays, a contrast was not tested if there were fewer than two non-missing values in either training group.

Covariates were selected from assay-specific technical metrics that explained variance in the data and were not correlated with exercise training: log_2 CHEX4 for the immunoassays, which is a measure of non-specific binding[108]; RNA integrity number (RIN), median 5'-3' bias, percent of reads mapping to globin, and percent of PCR duplicates as quantified with Unique Molecular Identifiers (UMIs) for RNA-Seq; fraction of reads in peaks and library preparation batch for ATAC-seq. The same covariates were used in both differential analysis approaches described above.

Training group and sex permutation analyses

Permutation tests were based on 100 permutations of the transcriptomics data in each tissue. First, we performed permutation tests of groups within sex. For each sex, the original group labels were shuffled to minimize the number of animal pairs that remain in the same group. For example, if there were 5 animals in week 1 (1w), in the shuffled labels each animal was put randomly in a different group (controls,1w, 2w, 4w, or 8w). For differential analysis, only the group labels were shuffled and all other covariates remained as in the original data. For each permuted dataset we reran the differential abundance pipeline and counted the number of transcripts that were selected at 5% FDR adjustment. Second, we performed permutation tests of sex within groups. For each group and each sex, half of the animals were selected randomly and their sex was swapped. Only the sex labels were shuffled and all other covariates remained as in the original data. For each permutation we reran the differential analysis pipeline and extracted the timewise summary statistics. A gene was considered sexually dimorphic if for at least one time point the z-score (absolute) difference between males and females was greater than 3.

Meta-analysis of metabolomics results

The metabolomics data were collected using different platforms from six different sites. Moreover, some platforms were *targeted* (i.e., quantified a predefined small set of metabolites of interest), whereas other platforms were *untargeted*. There were 1116 cases in which at least two sites measured the same metabolite in the same tissue. In these cases, we used meta-regression to integrate the differential analysis results, implemented using R's *metafor* package[114].

For a given metabolite m, the input to this analysis included the timewise effect sizes $y_{g,p}$ and their variances $v_{g,p}$ where g denotes the analysis group, which is a combination of the training time point and the sex for which the summary statistics were computed using the regression models explained above, and p ($\in 1,...,n_m$) denotes the platform. If m had data from at least three platforms, of which at least one was untargeted and at least one was targeted, then we added nested random effects for both the platform and the targeted status. That is, in metafor's notation we used: "mods ~ 0+analysis_group" and "random = list(~analysis_group|platform, ~analysis_group|is_targeted)". The inner|outer notation defines a blockwise dependence structure for the random effects, where different outer values are assumed to be independent, and the same outer values may be dependent based on their inner values. If the targeted status was redundant (e.g., we only had two platforms, one was targeted and the other was untargeted) then we kept the platform-level random effects only.

In practice, we observed that in 154 cases the default metafor optimizer failed to converge. In these cases, we modified the default parameters to optimizer = "nloptr", algorithm = "NLOPT_LN_SBPLX". Still, optimization failed in 61 additional cases. When both the default and alternative optimizers failed, we opted for a standard fixed effect model without random effects. Other than these cases, we had 361 models with random effects for both the platform and the targeted status, and 694 models with a platform-only random effect.

As a summary of each model we kept the overall model p-value (i.e., the modifiers p-value) *QMp* (used as the training p-value), and the residual heterogeneity p-value *QEp*. We flagged 103 models with QEp < 0.001 as having excessive heterogeneity. Using this definition we partitioned the meta-analysis results into three classes: (1) excessive heterogeneity, and has a targeted platform (57 cases), (2) excessive heterogeneity, without a targeted platform (46 cases), and (3) low heterogeneity (1013 cases). For class (2) we discarded the meta-analysis and kept the all platform-level results for m as-is. For class (1) we discarded the meta-analysis and kept only the targeted platform-level results for m. For class (3) we kept the meta-analysis results only (i.e., discarded the platform-level results), and used the meta-regression model to calculate the timewise summary statistics, i.e., time- and sex-specific meta-analysis effect sizes, their variance, and their p-values.

Graphical clustering of differential analysis results

Given the timewise differential analysis summary statistics for each analyte, computed as explained above, we next clustered the analytes into homogeneous patterns. Ovary, testis, and vena cava results were excluded from this analysis because not all time points and sexes were represented in the samples, which is a prerequisite for this longitudinal approach.

Standard clustering analysis can be used for identifying the main patterns in the data. For example, by generating a matrix of z-scores from the timewise summary statistics, where analytes are the rows and the eight training groups (e.g., 1-week males) are the columns, standard clustering algorithms can be applied. However, this methodology has three main limitations. First, off-the-shelf clustering algorithms use standard distance or correlation metrics to compute analyte pairwise distances. Thus, this may give the same distance between -1 to 1 as between 1 and 3. In the z-score space, the latter may be more important as it can signify a difference between a significant discovery (z=3) and a borderline result (z=1). Moreover, standard distance metrics do not directly account for correlations over sex and time. Second, standard clustering and fuzzy clustering analyses lead, by definition, to information loss. For example, clustering the analytes has a limited descriptive power in the presence of *split* or *convergence* points over the longitudinal trajectories. For an example of a split point in time, consider a group of features that have the same upregulation pattern up to week 4, but only a subset of the features returns to baseline levels in week 8, while the rest remain up-regulated. Clustering analysis will likely result in two clusters, without showing a direct connection between them. Finally, clustering algorithms typically disagree about the number of clusters in the underlying data, especially in noisy data. For example, a parametric mixture of gaussians clustering using the *mclust* R package[115] tends to estimate the number of clusters at 28-30 (depending on internal parameters of the algorithm). However, in practice we found that the solutions of these algorithms tend to result in a very large cluster around the origin, with excessive standard deviations in each axis, resulting in many significant z-scores in this “null” cluster. Thus, this algorithm does not provide reasonable results for our matrix of z-scores. As an alternative that utilizes our additional information about the data, we implemented a solution that overcomes the limitations of standard clustering. First, we model z-scores directly using a mixture distribution to separate null from non-null cases, thereby circumventing the limitation of

naive distance metrics explained above. Second, we learn the joint distribution of events over the groups, thereby accounting for correlations over time between sexes. Finally, we utilize a graphical framework for representing the time-course of differential events. This provides an enhanced representation that allows for the identification of both split and convergence events over time.

To set the stage for our graphical clustering solution we start with some notation. Let $Z \in R^{n\,x\,t\,x\,2}$ be the timewise z-scores, where $z_{i,j,k}$ denotes the z-score of analyte *i* (∈ 1,...,n) computed at the training time point *j* (∈ 1,...,t) of sex *k* (∈ {m,f}, for males and females, respectively). Assuming that $z_{i,j,k}$ follows a mixture distribution of null and non-null z-scores (i.e., a standard two-groups model), then each $z_{i,j,k}$ has a latent configuration $h_{i,j,k} \in \{-1,0,1\}$, where -1 denotes down-regulation, 0 denotes null (no change), and 1 denotes up-regulation. For simplicity, let **h** $\in \{-1, 0, 1\}^{t\,x\,2}$ denote a *full configuration matrix* (e.g., specifying if a z-score is null, up, or down for each time point in each sex), and let $\mathbf{z}_i \in R^{t\,x\,2}$ be the matrix of all z-scores of analyte *i*. We used the expectation-maximization (EM) process of the repfdr algorithm[116,117] to estimate for each possible **h** both its prior probability π(**h**) and its posterior Pr(**h**|$\mathbf{z}_i$), for every analyte *i*. In this process, repfdr infers the marginal mixture distribution of each time point *j* and sex *k*. That is, all z-scores z_{*jk} are used to estimate the densities: $f_{j,k}(z|H_{i,j,k} = -1) = f_{-1,j,k}(z)$, $f_{j,k}(z|H_{i,j,k} = 0) = N(0,1)$, and $f_{j,k}(z|H_{i,j,k} = -1) = f_{1,j,k}(z)$. This is done using the *locfdr* R package (see [118] for details). Given these densities, the EM process is used such that Z is the observed data and H is the missing data. It iteratively updates the estimates for π(**h**) and Pr(**h**|$\mathbf{z}_i$) to increase the overall complete composite likelihood (see [117] for details).

Once the algorithm converges, we discard configurations **h** with π(**h**) < 0.001 and normalize Pr(**h**|$\mathbf{z}_i$) to sum to 1 (i.e., all posteriors given the same $\mathbf{z}_i$). The main output of the EM for our analysis are these new posteriors that can be interpreted as a soft clustering solution, where the greater the value is, the more likely it is for analyte *i* to participate in cluster **h**. Of note, repfdr makes two simplifying assumptions about the data: (1) the z-score patterns of the analytes are independent, and (2) for a specific analyte *i*, the z-scores across the groups are independent conditioned on **h**.

Given the posteriors Pr(**h**|$\mathbf{z}_i$) computed above, we assign analytes to “states”. A state is a tuple ($s_{m,j}$, $s_{f,j}$), where $s_{m,j}$ is the differential abundance state null, up, or down (0,1, and -1 in the notation above, respectively) in males at time point *j*, and $s_{f,j}$ is defined similarly for females (at time point *j*). Thus, we have nine possible states in each time point. For example, assume we inspect analyte *i* in time point *j*, asking if the abundance is up-regulated in males while null in females. Then, we sum over all posteriors Pr(h|z_i) such that $h_{m,j}$=1 and $h_{f,j}$=0. If the resulting value is greater than 0.5, then we say that analyte *i* belongs to the node set S($s_{m,j}$, $s_{f,j}$). Thus, we use S($s_{m,j}$, $s_{f,j}$) to denote all analytes that belong to a state ($s_{m,j}$, $s_{f,j}$). Then, for every pair of states from adjacent time points *j* and *j+1* we define their edge set E($s_{m,j}$, $s_{f,j}$, $s_{m,j+1}$, $s_{f,j+1}$) as the intersection of S($s_{m,j}$, $s_{f,j}$) and S($s_{m,j+1}$, $s_{f,j+1}$). Thus, the sets S and E together define a tree structure that represent different differential patterns over sex and time.

Comprehensive feature-to-gene map

We compiled a feature-to-gene map that associates every feature tested in our differential analysis with gene identifiers. All proteomics feature IDs (RefSeq accessions) were mapped to gene symbols and Entrez IDs using NCBI's "gene2refseq" mapping files (https://ftp.ncbi.nlm.nih.gov/gene/DATA/gene2refseq.gz, accessed 12/18/2020). Epigenomics features were mapped to the nearest gene using the R function *ChIPseeker::annotatePeak()*[119] with Ensembl gene annotation (*Rattus norvegicus* release 96). Gene symbols, Entrez IDs, Ensembl IDs, and Rat Genome Database (RGD) IDs were mapped to each other using the RGD rat gene annotation (https://download.rgd.mcw.edu/data_release/RAT/GENES_RAT.txt, accessed 11/12/2021).

Pathway enrichment analysis of graphical clusters

All non-metabolite training-differential features (5% IWH FDR) were mapped to Ensembl gene symbols using the feature-to-gene map described above. Training-differential metabolites were mapped to KEGG IDs. For each graphical cluster of interest (i.e., the ten largest paths, two largest nodes, and two largest single edges with at least 20 features in each tissue), we performed pathway enrichment analysis separately for the Ensembl genes (or KEGG IDs for metabolomics) associated with differential features in each ome. For most omes, background gene (or metabolite) sets were specified as the set of Ensembl IDs (or KEGG IDs) associated with all features tested within the corresponding tissue during differential analysis. For epigenomics (ATAC-seq and RRBS), the background gene sets were defined as the set of all genes expressed in each tissue, taken from our RNA-Seq data. For gene-centric omes (i.e., all but metabolomics) we performed enrichment analysis of KEGG and REACTOME rat pathways (organism "rnorvegicus") using the *gprofiler2::gost* function in R with the custom backgrounds defined above[120]. Only pathways with at least 10 and up to 200 members were tested. Because *gprofiler2::gost* only returns adjusted p-values, we recalculated nominal p-values using a one-tailed hypergeometric test, which is consistent with how *gprofiler2::gost* calculates enrichments. For metabolites, we performed enrichment of KEGG pathways using the hypergeometric method in the R *FELLA* package with custom backgrounds as defined above[121]. Pathway enrichment analysis p-values were adjusted across all results using Independent Hypothesis Weighting (IHW) with tissue as a covariate. We defined significantly enriched pathways as those with q-value < 0.1. Significant pathway enrichments driven by a single gene were removed from the results (Extended Data Table 11).

Molecular interaction networks

We considered three resources for biological interactions: BioGRID (v BIOGRID-ORGANISM-4.2.193.tab3)[21], STRING (v 10116.protein.physical.links.v11.5)[122], and biological pathways. BioGRID and STRING were used for protein-protein interactions. Biological pathways contain various types of interactions, including undirected protein interactions, directed signaling interactions, and gene-metabolite pairs. For the analysis below we ignored the type (and direction). Pathway interactions were retrieved using the *graphite* R package (v

1.37.1)[123,124]. We used all directed and undirected (but not indirect) pathway interactions from Reactome, PathBank, and KEGG[125–127]. Metabolite IDs from these pathways were mapped to KEGG and InChiKey IDs (if ones were not provided). Metabolites that only had ChEBI or CAS identifiers were mapped to KEGG and InChiKey IDs using the CTS online tool (http://cts.fiehnlab.ucdavis.edu/batch). Finally, KEGG and InChiKey IDs were mapped to RefMet IDs using the Metabolomics Workbench REST service. A few extra missing KEGG IDs were added manually (as provided by chemical analysis sites).

The rat STRING network contained 115,389 high confidence interactions (score > 500), covering 10,322 genes. The graphite rat gene-gene network contained 161,130 interactions, covering 8942 genes and metabolites. The rat gene-metabolite pathways network contained 95,295 interactions, covering 5791 genes and metabolites. In contrast, the BioGRID rat network was much smaller with 6408 interactions only. To account for this limitation we used the human and mouse networks to extend the rat BioGRID network. By mapping gene symbols from these organisms to rat gene symbols via the RGD ortholog mapping (v39), we were able to generate a BioGRID rat network with 461,685 interactions, covering 15,856 genes. When analyzing data from a specific tissue, all networks were reduced to their induced subgraphs using the background gene and metabolite sets explained above.

Network connectivity analysis

Given an interaction network G=<V,E> (where V is a set of nodes, and E is a set of undirected edges), a “reference” set of nodes S, and a node of interest v, we tested the connectivity between v and S in G using a simple hypergeometric test. Assume a null hypothesis in which all neighbors of v in G are selected at random. Under this null hypothesis, the number of neighbors of v that are in S follows a hypergeometric distribution HG(N,K,n), where N is the number of nodes in the graph G (i.e., |V| the number of nodes in V), K is the number of neighbors of v in G, and n=|S| is the number of nodes in S. Thus, for each node v in G we can obtain a p-value for the connectivity of v and S.

To illustrate the tight connectivity of our multi-omic results in the gastrocnemius (SKM-GN), we examined the sex-consistent, up-regulated analytes identified in week 8. These data contained 64 multi-omic genes identified by at least two different omes. We used this set of genes as the reference set S, and for each of the three gene networks above we computed the p-value for every node. For a node v in S the p-value was computed by taking S\{v} as the reference set (i.e., by excluding v from S). In each network we selected the significant nodes at 5% Benjamini-Hochberg FDR adjustment, resulting in 95, 51, and 326 genes in the STRING, pathways, and BioGRID networks, respectively. These sets were significantly enriched both in the multi-omic genes in S and in genes that were discovered by a single ome in our analyses (p < 10^-10 for each set using Fisher’s exact test; see Extended Data Fig. 9b).

Based on the analysis above we took the BioGRID network interactions and used them to generate an interaction graph of all genes and metabolites identified in SKM-GN 8w_F1_M1 (i.e., up-regulated in both sexes in week 8). We clustered the graph using the leading

eigenvector clustering algorithm[128], implemented in the *igraph* R package[129]. This resulted in three large connected clusters that had at least 10 nodes (Extended Data Table 13).

Transcription factor enrichment analysis methods

We conducted transcription factor (TF) motif enrichment analysis on sets of genes that satisfied the IHW-adjusted training p-value threshold of 0.05 for differential expression in a given tissue, isolating the 13 tissues that had at least 300 genes meet the differential expression threshold. The analysis was carried out by findMotifs.pl (HOMER v4.11.1)[130]. Enrichment of a motif in a tissue is quantified by the percent of promoter regions of differentially expressed genes containing that motif, with enrichment p-values calculated by comparing enrichment among target genes to enrichment among a background gene set that is normalized for GC%. To determine tissue similarity in motif enrichment, we calculated the mean absolute value of differences in enrichment over all TFs between each tissue and conducted hierarchical clustering of the tissues on their enrichment differences.

For more detailed analyses, we selected the top ten enriched TFs from each tissue and then removed any TFs whose corresponding genes were not found to be expressed in the RNA-Seq data. Pearson correlation was calculated between tissue-standardized TF motif enrichment scores and tissue-standardized TF gene expression across the set of control samples in each of the 13 tissues.

Rat-to-human ortholog map

We compiled a map between rat Ensembl IDs and human gene symbols using several external sources. GENCODE metadata and annotation files were used to map between human Ensembl transcript IDs, Entrez IDs, GENCODE IDs, and Ensembl gene IDs[131]. RGD files were used to map between human and rat gene symbols as well as between various rat gene identifiers[132].

Gene and PTM set enrichment analysis

Gene set enrichment analysis (GSEA) and PTM set enrichment analysis (PTM-SEA) were performed using the ssGSEA2.0 implementation by Krug et al.[133]. For GSEA, differential enrichment analysis t-scores (trained versus control) were used as input. Feature-level data was summarized into gene-level data using the maximum absolute t-score. The gene set database used included canonical pathways available through the Human Matrisome database available through MatrisomeDB (http://matrisome.org/), and the MitoPathways database available through MitoCarta 3.0[134–136]. Human gene symbols were mapped to rat orthologs before running the analysis. The *ssGSEA2* function was run using parameters that avoid normalization, require at least 5 overlapping features with the gene set, and use the area under the curve as the enrichment metric (sample.norm.type = "none", weight=0.75, correl.type = "rank", statistic = "area.under.RES", output.score.type = "NES", min.overlap=5).

For PTM-SEA, phosphosite-level t-scores were used as input. Phosphosite flanking sites were mapped from rats to humans as described below. The PTM set database used was the human PTMSigDB[133]. The *ssGSEA2* function was run using parameters that avoid sample normalization, require at least 5 overlapping features with the gene set, and use the area under the curve as the enrichment metric (sample.norm.type = “none”, weight = 0.75, correl.type = “rank”, statistic = “area.under.RES”, output.score.type = “NES”, min.overlap = 5).

Mapping PTMs from rat to human proteins

We used the NCBI Reference Protein Sequence database (RefSeq) to annotate protein IDs. Most of the Post-Translational Modification (PTM) resources and tools available are for humans; rat annotation is lacking. To leverage information from humans, we mapped PTM sites from rats to humans following a bioinformatics approach. Briefly, we used BLAST+ v2.11.0[137,138] to align all rat sequences to the UniProt human proteome FASTA for canonical protein sequences (UniProtKB query "reviewed:true AND proteome:up000005640", download date 02/03/2021)[139]. The median protein sequence identity between rats and humans is 85%. Only alignments with a sequence identity greater than 60% were included for mapping. For most proteins, BLAST+ outputs multiple pairwise alignments (one-to-many). In those cases, we selected the alignment with the larger "positives" and "identities" values and required an exact match for the S/T/Y residues identified in this study. As a result, we could map with confidence 73.5% of all the phosphorylation sites we identified.

Correlation of training-differential features with cell type markers

In order to characterize the extent to which the presence of different cell type populations were reflected in trajectories of specific clusters of training-differential features, we correlated these trajectories with transcript- and protein-level expression of cell markers. Well-documented, immune cell-type-specific surface markers classically employed in tissue staining and cell sorting procedures were selected through literature review and using resources from companies such as ThermoFisher and Biolegend (Extended Data Table 20). Hemoglobin genes were added to the list of erythrocyte markers. Marker of proliferation Ki-67 (*Mki67*) was also included to reflect cell proliferation[140], and markers of lymphatic tissue were curated from the literature[141]. For each marker, we calculated the Pearson correlation between the normalized expression of the marker and the average normalized expression across all features in the given cluster of interest. Correlations were performed on sample-level data, separately for each tissue and ome (transcriptomics and proteomics). For each cell or tissue type with more than one marker, we performed a one-sample t-test to assess whether or not the corresponding correlations were significantly different from zero, separately for each tissue and ome (5% BY FDR).

Immune cell type deconvolution

In addition to correlating gene expression with specific immune cell type markers, we performed immune cell type deconvolution on bulk, tissue-level transcriptomic data using CIBERSORTx (v1.05)[142]. Ensembl gene IDs were mapped human gene symbols using the rat-to-human

ortholog map described above, and raw read counts from each tissue were deconvoluted using the LM22 immune cell signature matrix generated by CIBERSORT developers[143] with the following parameters: absolute mode, B-mode batch correction, no quantile normalization, 0 permutations for significance analysis. The resulting absolute scores for each cell type in each sample were used for statistical analyses and comparisons within each tissue (Extended Data Fig. 12e).

Enrichment of LM22 immune cell types

In a parallel approach to immune cell type deconvolution, we performed enrichment of the CIBERSORT LM22 immune cell types using cell type specificity scores calculated from the LM22 leukocyte gene expression matrix[143,144]. The specificity index (SI) was calculated for each gene and cell type on a gene by cell-type matrix of the average expression for each cell type using the *pSI::specificity.index* R function with arguments e_min=0, bts=1, and SI=TRUE[145]. The resulting SI were $\log_2$-transformed and multiplied by -1 so that larger scores corresponded to greater cell type specificity. The rat-to-human ortholog map described above was used to map human gene symbols from the LM22 dataset to rat gene identifiers. For each immune cell type, tissue, and graphical node of interest (e.g., memory B cells, white adipose tissue, 8w_F1_M1), we tested for enrichment of immune cell type signatures by comparing the cell type specificity scores corresponding to the training-differential transcripts in the given tissue and cluster to the cell type specificity scores corresponding to all other genes expressed in that tissue using a one-sided Mann-Whitney U test (*wilcox.test* function in R with argument alternative = “greater”). Significant enrichments were reported at q-value < 0.05 across all tests (Extended Data Fig. 12f).

Quantification of sex differences in the training response

The extent of sex differences in the training response were characterized in two ways. First, we correlated the training $\log_2$ fold-changes between males and females for each training-differential feature in order to characterize differences in the direction of the training effect. A zero was prepended to the time-ordered vector of $\log_2$ fold-changes for each sex to indicate baseline (i.e., the $\log_2$ fold-change between the controls trained for zero weeks and themselves is zero). In order to account for spurious association due to autocorrelation[146], we first took the lagged difference of values in each vector and divided those values by the square root of the lagged differences in the time points (i.e., 0, 1, 2, 4, and 8 weeks). Then we calculated the Pearson coefficient between these transformed vectors (Extended Data Fig. 10). Second, we calculated the difference between the male and female areas under the curve (Δ_{AUC}, males - females) made by plotting time versus $\log_2$ fold-change for each training-differential feature, including a (0,0) point. This characterized the difference in the magnitude of response between the sexes. All $\log_2$ fold-changes for a feature were scaled to have unit variance before calculating sex-specific AUC using the *pracma::trapz* function in R[147]. Because Δ_{AUC} does not account for time, it is difficult to interpret alone. Therefore, we plotted the Δ_{AUC} against the correlation between male and female training $\log_2$ fold-changes (Extended Data Fig. 10) to

visualize the sex difference in both magnitude (x-axis) and direction (y-axis) of the training response for all training-differential features in each tissue.

Comparison with extant training response omics data

We first compared our results with the gene expression data meta-analysis of Amar et al.[8]. Amar et al. used meta-analysis to synthesize skeletal muscle gene expression data from 26 cohorts (n=430 subjects). Each cohort had a group of subjects that went through a training program and had a pre-training time point and at least one post-training time point. Most of the studies collected data from vastus lateralis. They identified 114 genes that were consistently up-regulated across the studies. However, these 114 genes were selected at extremely strict cutoffs, and extensive effect heterogeneity of many other genes were observed. We compared our vastus lateralis transcriptomics differential analysis results with the results of the meta-analysis. First, we computed the significance of the Spearman correlation between our estimated $\log_2$ fold-changes and the estimated $\log_2$ fold-change from the meta-analysis (Extended Data Fig. 9d-e). Second, we ranked all available human genes according to their meta-analysis statistics and then performed gene set enrichment analysis (GSEA) using our identified gene sets as the sets of interest. For this analysis we used our sex-consistent differentially expressed gene sets (i.e., F1_M1, F-1_M-1, and F0_M0 according to our graphical analysis notation) from all weeks (Extended Data Fig. 9f). For this GSEA, the human genes were ranked using: ($-\log_{10}$ meta-analysis p-value)*(meta-analysis $\log_2$ fold-change). Finally, we checked the overlap between our gene sets and the set of genes with excessive true heterogeneity from the human data. These were defined as genes with a meta-analysis I2 > 75%. We used Fisher's exact test for testing the null hypothesis of a random overlap (Extended Data Fig. 9g).

We also compared our results to the data from Bye et al.[22]. These are microarray-based gene expression data collected from the soleus of female rats before or after 8 weeks of training. Animals exercised for 1.5 hours 5 days per week with intervals between 50-90% VO_2max. Similarly to our study, animals were sacrificed 48 hours after the last exercise bout after 8 weeks of training. Bye et al. also had two study arms: high capacity runners (HCR) vs. low capacity runners (LCR). Gene expression profiles were obtained from the Gene Expression Omnibus (GEO) record GSE10527. Sample .CEL data were RMA-normalized, and all resulting expression profiles were quantile-normalized together. We then used limma for differential analysis to quantify the differential expression of genes before and after training for both the HCR and LCR groups. We then compared the resulting differential analysis results to our week 8 female transcriptomics results of the vastus lateralis and gastrocnemius (both skeletal muscle tissues, but are different from the soleus). We computed Fisher's exact test p-values, comparing the reported gene sets by both studies at 0.01 significance (Extended Data Fig. 9h).

For proteomics, we compared our results to data from Hostrup et al.[23]. In this study, eight untrained men (23–38 years of age) completed a high intensity interval training (HIIT) regimen that consisted of 5 weeks of supervised cycling, performed as 4–5x4 min intervals at a target heart rate of >90% max interspersed by 2 min of active recovery, undertaken three times weekly. Samples from the vastus lateralis were obtained before and after the regimen, and both

protein expression and lysine acetylation were quantified. We compared our gastrocnemius global protein abundance results with this study (vastus lateralis had no proteomics data, and gastrocnemius had no acetylation data). Differential abundance results for proteomics from the original study were obtained from their supplementary material. As in the Bye et al. data analysis, we used Fisher's exact test p-values, comparing the reported gene sets by both studies at 0.01 significance (Extended Data Fig. 9i).

Disease ontology enrichment analysis

We first preprocessed the disease ontology database before applying the enrichment analyses. Our rationale here was that many disease terms may be enriched with general biological processes that are relevant for many tissues both in health and disease states (e.g., cell proliferation in cancer disease terms), and are thus not likely to reflect a true association between our exercise-specific results and diseases. We therefore generated tissue-specific disease ontology terms by utilizing gene expression data from GTEx v8[148]. For each disease ontology term and a tissue (covered by GTEx) we computed the p-value for the overlap between the term's gene set and the tissue's gene set. If the p-value was greater than 0.001 then we omitted the term from the tissue's analyses. Disease ontology enrichment analysis was then performed using the *DOSE* R package[24] for each of our tissue- and ome-specific gene sets that had at least 20 genes. Identified associations were declared as significant if their FDR-adjusted p-value was <0.05 and they had at least three genes in the intersection between our set and the disease term gene set.

Metabolite enrichment by RefMet class

Enrichment of metabolite classes were calculated from training-regulated metabolites in each metabolite class and tissue using ssGSEA2.0[133]. RefMet Main Classes were used as the metabolite sets input (Extended Data Table 26), and the metabolite $-\log_{10}$ training p-values were used as input scores (Extended Data Table 25). Given that these are broad metabolite classes that may include various biological processes, we performed the enrichment using only the training-associated p-values without considering directionality or temporality. As training p-values do not indicate the temporality, direction, or sex-specificity of training-induced changes, we considered significant enrichments (q-value<0.05) with positive normalized enrichment scores (NES>0) positive results. A positive result indicates that metabolites in the given class were disproportionately regulated by training in the given tissue.

SUPPLEMENTARY DISCUSSION

Multi-omic analysis of exercise training

Details for each omic analysis performed

Transcriptomic data from RNA sequencing (RNA-Seq) were collected in all 18 solid tissues and the blood (n=5 per sex and time point); epigenomic data from transposase-accessible chromatin using sequencing (ATAC-seq) and reduced representation bisulfite sequencing (RRBS) were collected in 8 tissues (n=5 per sex and time point). Proteomic data were generated by LC-MS/MS for 7 tissues (n=6 per sex and time point) using TMT-based quantification where each plex contained a common tissue reference to enable quantification across plexes (Extended Data Fig. 2a). Global proteome and phosphoproteome data were acquired in all 7 tissues, whereas acetylome (acetylated lysines; acetylsites) and ubiquitylome (K-ε-GG ubiquitin remnants; ubiquitylsites) were acquired from the liver and heart. Metabolomic data were generated for plasma and all 18 solid tissues using up to seven targeted platforms, four untargeted metabolomic platforms, and two untargeted lipidomic platforms (n=5 per sex and time point). Multiplexed immunoassay panels were applied to 17 tissues to accurately quantify low-abundance proteins of biological relevance, including cytokines. These datasets were generated using five different rat panels (54 analytes total; n=3 per sex and time point). For each ome, QC metrics were used to ensure the data were of high quality (Extended Data Fig. 2b-k; Methods). Across assays, only 98 samples (1.0%) were identified as technical outliers and excluded from downstream analysis (Supplementary Table 1; Methods).

Permutation tests demonstrate significant responses to endurance training

We utilized permutation testing to identify tissues with significant endurance-training-induced changes in differential gene expression (Extended Data Fig. 3a-b). Except for the hypothalamus and hippocampus, every tissue was significant in at least one sex ($p \leq 0.01$), and cases with $p > 0.05$ tended to correspond to tissue and sex combinations with very few differential transcripts. We also permuted sex labels within training groups to identify tissues with a significant sexually dimorphic endurance training response (Extended Data Fig. 3c-d). Most tissues were significant at $p < 0.05$, and seven tissues were significant at $p \leq 0.01$ (adrenal gland, blood, heart, kidney, lung, vastus lateralis, and white adipose).

Multi-tissue response to training

Sexually conserved responses reveal tissue-dependent adaptations to endurance training

We identified pathways enriched by tissue-specific exercise-responsive genes (Extended Data Fig. 4b) and tabulated a subset of highly-specific genes to gain insight into tissue-specific training adaptation (Supplementary Table 4). It is important to note that the pathway enrichment results were derived from training-regulated features without constraints on when or how they were regulated during the training time course. Therefore, directionality of the enrichments is not interpretable; we instead highlight the temporal dynamics of specific features.

Focusing on sex-consistent adaptations in the lung, revealed temporal changes in features related to immune cell recruitment and tissue remodeling (Extended Data Fig. 4b). For example, the neutrophil marker S100A8 decreased at 2 weeks in both sexes, while immunoregulatory CD109[149] decreased at all training timepoints in males and at 8 weeks in females (Supplementary Table 4). THSD7A, a protein with angiogenic functions[150], increased with training in both sexes (Supplementary Table 4). These findings highlight candidate mechanisms by which exercise training promotes lung health and remodeling. The kidney displayed ribosome pathway enrichments (Extended Data Fig. 4b); specific up-regulated features included neuron-specific *Cabp1*[151] and *Hsd17b1,* which is involved in sex hormone steroidogenesis[152] (Supplementary Table 4), highlighting novel physiological adaptations in the kidney that may impact systemic homeostasis. Liver-specific training-regulated genes drove an enrichment for cofactor and cholesterol biosynthesis (Extended Data Fig. 4b) and included ACAT2 and TM7SF2 in both sexes and HMGCR and *Mvk* in males (Supplementary Table 4). Such adaptations may contribute to beneficial effects of endurance training on circulating lipid profiles[153]. In the heart, unique features up-regulated with training included genes related to ion flux, such as the potassium channel-associated genes *Kcnip2* and *Kcnk3*, which are down-regulated in heart disease and regulate cardiomyocyte polarization[154,155] and TAX1BP3, which promotes internalization of the potassium channel KCNJ4 and whose mutations are linked to dilated cardiomyopathy[156] (Supplementary Table 4). Further, both sexes showed consistently decreased phosphorylation of RRAGD T390, a transducer of amino acid-induced mTORC1 activation[157], which is key in cardiac remodeling (Supplementary Table 4). Together, these highlight potential candidate mediators of cardiac remodeling with endurance training. The gastrocnemius displayed multiple pathway enrichments related to metabolic processes and striated muscle contraction (Extended Data Fig. 4b), with downregulation of genes related to ER structure and function (LNPK, VAMP5, NRBP1, SLC37A4, *Ptpn1*), redox (NMRAL1, SELENOF), and N-glycan biosynthesis (MAN2A2) (Supplementary Table 4). In the white adipose tissue, pathway enrichments were related to extracellular matrix reorganization (Extended Data Fig. 4b), with upregulation in genes related to neurogenesis and neuroendocrine cellular regulation (MCFD2, ATP6AP1, TXLNA) (Supplementary Table 4). MCFD2 maintains stemness of adult neuronal progenitors[158]; given white adipose is primarily sympathetically innervated, this highlights a potential mechanism for enhanced neuronal control of lipolysis[159].

Endurance training induces a multi-tissue protein-specific increased in the abundance of heat shock proteins

We considered the six deeply profiled tissues for a gene-level analysis across tissues (Heart, gastrocnemius, white adipose tissue, liver, lung, and kidney). As mentioned in the main text, heat shock proteins (HSPs) showed a significant increase in protein abundance across all of these tissues (Extended Data Fig. 5). Other notable genes responding to exercise training in five of these six tissues considered include the carnitine acetyltransferase *Crat*, a regulator of metabolic flexibility[160], *Fscn1*, a marker of neurogenesis[161], *Hadha*, a mitochondrial enzyme that promotes long chain fatty acid oxidation, and *Tns1*, a focal adhesion molecule with roles in cellular signaling and proliferation, including angiogenesis[162] (Supplementary Table 3). The HSP family has important cytoprotective functions[163] and is dysregulated in metabolic diseases like diabetes[164]. In humans, the major HSPs, HSPA1A/HSPA1B and HSP90A1AA (a.k.a HSP70 and HSP90, respectively) were observed to increase in protein abundance within skeletal muscle of lifelong footballers and highly trained rowers[165,166]. HSP accumulation can therefore explain some of the cytoprotective and other health benefits associated with exercise[167,168]. The discordance between transcript and protein abundance is likely due to the sample collection occurring 48 hours after the last exercise bout, where transient changes in transcripts following acute exercise are expected to return to baseline. In agreement, HSP70 transcript levels have been described to increase immediately after exercise[169], while the increased protein abundance has been observed up to 6 days after exercise[170]. Moreover, none of the measured PTMs could explain the striking change in protein abundance. The differences in alteration of HSPs at both the protein and transcript level illustrate the importance of simultaneously measuring multiple omes to fully understand the response to endurance training, and further research is required to explain the molecular mechanism behind this striking observation.

Unique transcript and protein responses

The discordance between transcript and protein abundances of HSPs reflects a broader low to moderate correlation between the transcriptome and proteome, which, as mentioned above, is likely due to collecting samples 48 hours after the last exercise bout (Extended Data Fig. 4c). Similarly low correlation between the transcriptome and proteome was observed at the pathway level using gene set enrichment analysis (GSEA) (Extended Data Fig. 4c, Methods). Several pathways showed strong agreement while others showed ome-specific enrichment (Extended Data Fig. 4d; Supplementary Tables 5-6). For example, the oxidative phosphorylation pathway had a strong positive enrichment at both the transcript and protein levels in the gastrocnemius, while it only showed positive enrichment for the proteome in the male heart and white adipose tissue (Extended Data Fig 4d). The cilium assembly pathway was positively enriched in lung proteomics data, while only the male lung additionally showed enrichment at the transcript level (Extended Data Fig 4d). Cilia are important protective structures in the lung; therefore, its regulation has important implications for lung function[171]. The low correlation between the transcriptome and proteome is in agreement with numerous previous studies showing that exercise triggers a transient increase in transcript abundance, with longer-lasting responses at

the protein level[172–174]. For example, many myogenic and metabolic genes reach peak expression at 4-8 hours post-exercise, and their transcript levels return to baseline after 24 hours[175]. In our study, transient changes in transcripts following acute exercise are expected to return to baseline before the collection time point. Altogether, these results highlight the value of profiling multiple omes and tissues, where we observe many unique and shared exercise training responses that would not otherwise be captured.

TF activity and phosphosignatures

TF enrichment in non-skeletal muscle tissues

Inference of transcription factor activities and phosphosignature changes reveal candidate mechanisms of tissue adaptation to training. In addition to hematopoietic-associated TFs (GABPA, ETS1, KLF3, and ZNF143) and MEF2 enrichments, the small intestine, lung, and brown adipose tissue displayed enrichment in IRF8, a TF highly expressed in dendritic cells[176]. The colon displayed upregulation in the circadian regulatory CLOCK TF (Fig. 3a; Supplementary Table 7). Circadian regulation of the gut positively impacts microbial diversity and homeostasis[177], highlighting a potential mechanism by which endurance training impacts gut health.

Phosphosignature analysis reveal mechanisms for exercise adaptation in liver and heart

In the liver, we observed a robust increase in the phosphosignature related to EGFR1, a key regulator of hepatic regeneration[178], as well as HGF (Extended Data Fig 6b). The HGF phosphosignature could be explained by an increase in HGF production[179] or HGF sensitivity in the liver. Exercise enhances liver health and metabolism[180,181], yet the mechanism for improved liver health is incompletely understood. In rats, HGF treatment improves conditions of alcohol-induced fatty liver through reduced fibrosis[182–184]. In agreement, we observed phosphorylation of HGF downstream targets, STAT3 and PXN (Extended Data Fig. 6c). Altogether this analysis identifies a potential mechanism of exercise-mediated improvement in liver health through HGF signaling.

In the heart, the predicted changes in kinases basal activity can explain heart remodeling in response to endurance training (Extended Data Fig. 6d). For example, we observed a decrease in the predicted activity of some AGC protein kinases, including AKT1, which is known to be involved in cardiac hypertrophy[185]. AMPK substrates also show decreased phosphorylation, suggesting decreased AMPK activity. AMPK is an energy sensor activated in the heart immediately after exercise that acts upstream of MEF2A to promote GLUT4 translocation and facilitate glucose uptake[186,187]. Given samples were collected 48 hours after the last bout of exercise and we observed MEF2A TF enrichment (Fig. 3a) in addition to numerous metabolic pathway enrichments in the heart throughout training (Supplementary Tables 5-6), a decrease in

predicted AMPK activity may reflect improved overall metabolism in the resting heart following training. In contrast, some tyrosine kinases were predicted to have increased activity primarily at earlier time points, including SRC and mTOR. SRC and mTOR signaling has been implicated in the regulation of cardiac hypertrophy and structural remodeling[188–191], a known beneficial physiological adaptation to exercise[192,193]. As mentioned in the main text, changes in SRC signaling may explain some of the changes in extracellular matrix proteins observed in the heart of endurance trained rats (Extended Data Fig. 6f-h ; Supplementary Table 9). Further investigation of such extracellular remodeling pathways altered by exercise may help elucidate differential mechanisms between physiological and pathological cardiac hypertrophic remodeling.

Molecular hubs of exercise adaptation

Analysis of up-regulated features at 8 weeks of training in both sexes

This section describes additional observation of physiological relevance for the pathway enrichments of up-regulated features in both sexes at 8 weeks of training. These are all related to data presented in Supplementary Table 12 and Extended Data Fig. 8. From these key pathway enrichments, the hippocampus displayed expression of transcripts involved in neuroplasticity, including *Grin2d*, a component of the N-methyl-D-aspartate (NMDA) receptor and the BDNF receptor, *Ntrk2*[194,195]. Endurance training is suggested to attenuate cognitive declines with aging by preserving hippocampal volume in part through increasing circulating BDNF levels[196], highlighting a potential central sexually conserved neuroprotective response to training. The heart displayed enrichments primarily at the proteomic level for several metabolic processes, including fatty acid, glucose, and amino acid catabolism, peroxisome and mitochondrial lipid metabolism, and mitochondrial biogenesis. Numerous metabolic pathways were up-regulated in both sexes at all training timepoints, highlighting early and prolonged cardiac adaptations to training (Supplementary Table 11). The liver also displayed pathway enrichments related to lipid metabolism, including mitochondrial and peroxisomal activity and biogenesis, in addition to steroid and bile acid biosynthesis at both the transcriptome and proteome level. Relative to other tissues, 8 weeks of training had substantial impacts on chromatin accessibility pathway enrichments in the liver, with both sexes displaying enrichments in nuclear receptor signaling (e.g., *Egfr*, *Foxo3*) and cellular senescence through transcription factors key in liver homeostasis (e.g., *Cebpb*, *Jun*)[197,198]. This further highlights potential mechanisms by which training improves liver health and reduces disease risk. The spleen displayed enrichments in terms related to phagocytes and complement activation while the kidney displayed protein-based pathway enrichments related to peroxisome activity and drug metabolism. White adipose tissue displayed changes in the methylation of features related to axon guidance and the protein abundance of features related to fatty acid and steroid metabolism. The adrenal gland displayed enrichment in transcripts related to lysosomes and sphingolipid and glycosphingolipid metabolism; sphingolipids are suggested to play a key role in

steroid hormone synthesis in the adrenal gland[199]. Future integration of these findings with changes in circulating hormones and HPA axis regulation may fortify our knowledge of the effects of endurance training on the systemic endocrine response.

Network connectivity analysis

We utilized a network connectivity analysis framework to characterize the multi-omic, sex-consistent, week 8, up-regulated features in the gastrocnemius at week 8. After mapping these features to genes, we observed modest yet significant overlaps between the transcriptomic, chromatin accessibility, and proteomic assays but not with the methylation assays (Extended Data Fig. 9a). We then compared three different resources of biological interaction networks: BioGRID[21], STRING[122], and biological pathways, including interactions from Reactome, PathBank, and KEGG[125–127]. Importantly, these networks were not filtered for specific functions in order to mitigate bias towards highly investigated pathways. Network connectivity analysis showed that all three networks had significant enrichments of interactions between genes identified by a single ome and multi-omic genes (i.e., genes identified by two or more omes), as well as among these multi-omic genes (Extended Data Fig. 9b; Methods). The undirected BioGRID network of physical interactions among proteins outperformed the other networks and was used for further analysis and interpretation.

Network eigenvector clustering[128] of the BioGRID network identified three clusters of genes (Extended Data Fig. 9c; Supplementary Table 13). The largest cluster had 181 nodes, was more balanced in its omic representation, and was significantly enriched (at 5% FDR) for multiple muscle adaptation processes, including: mitochondrion organization and fusion, aerobic respiration, PPAR and insulin signaling, longevity, muscle contraction, and response to heat and a mechanical stimulus (Fig. 4d; Supplementary Table 14). This analysis complements our previous pathway analyses by illustrating a direct linkage among these pathways and putative central regulators and network hubs including HSPs (e.g., HSP90AA1, HSPA4L, and HSPA1B), HDAC4, and MEF2C (Supplementary Table 13). In agreement, MEF2C was suggested to have increased activity in our TF enrichment analysis (Fig. 3a). Training-induced phosphorylation of HDAC4 by calcium/calmodulin-dependent protein kinase (CaMK) leads to its nuclear export and subsequent regulation of target genes[200,201], including GLUT4 for glucose uptake in muscle[202]. In the cytosol, non-histone substrates of HDAC4 include the master transcription cofactor for mitochondrial and oxidative metabolism, PPARGC1A, and several of the myosin heavy chain isoforms[203], linking the central network hub to both mitochondrial and structural remodeling in skeletal muscle.

From a computational perspective, this network analysis illustrates the utility of the multiomic data: limiting the data to a single ome causes separation into smaller subnetworks. For example, JUN is up-regulated only in the ATAC-seq data, HDAC4 and MEF2C have up-regulation of phosphoproteomics only, and HSPA4L is up-regulated mostly at the protein level (with some upregulation at the transcript level) (Supplementary Table 13). Thus, examining only known pathways or a single ome would limit the identification of similar, densely-connected multi-omic networks.

Connection to human diseases and traits

We integrated our rat-based data with human exercise studies and disease ontology annotations to gain translational insight. A significant correlation was observed between the meta-analysis-inferred fold-changes and our results for both sexes at 8 weeks of training ($p<1e-20$, Extended Data Fig. 9d-e). Focusing on our training-differential, sex-consistent genes, we observed a significant and direction-consistent enrichment of our gene sets in a GSEA analysis in which genes were ranked by their human meta-analysis results ($p<1e-5$, Extended Data Fig. 9f). Human gene expression data have been shown to exhibit excessive heterogeneity of fold-changes across studies due to observed and unobserved effect modifiers, including sex, age, training duration, and transcriptomics platform[8]. These effects substantially limit the power of the meta-analysis, suggesting a high false negative rate[8]. In our analysis we observed a significant overlap between the training-regulated rat genes and human genes with high fold-change heterogeneity, suggesting that the rat data may be leveraged to help identify exercise-responsive genes that were not detected by the human meta-analysis ($p<1e-4$, Extended Data Fig. 9g).

The disease ontology enrichment analysis helped pinpoint genes regulated in our rat study that are of relevance in human diseases, e.g., type 2 diabetes (T2D), heart disease, obesity, and kidney disease (5% FDR; Extended Data Fig. 9k). Leptin (*Lep*) and matrix metallopeptidase 2 (*Mmp2*) were down-regulated in white adipose tissue and are both associated with adipogenesis, T2D, and heart disease[204,205]. The proinflammatory agent Apolipoprotein C-III (*Apoc3*), down-regulated in white adipose tissue and the kidney, is associated with T2D, heart, and kidney diseases. *Apoc3* reduction in mice was recently demonstrated to protect white adipose tissue against obesity-induced inflammation and insulin resistance[206]. This highlights the value of this rat dataset for hypothesis testing in human exercise studies.

Sex-specific responses to exercise

Transcriptional remodeling of the adrenal gland

A large set of transcripts in the adrenal gland was negatively correlated between males and females, with sustained downregulation in females and transient upregulation at 1 week in males (Extended Data Fig 11d-e). Endurance training increases adrenal gland mass in both sexes, with more pronounced hypertrophy in male rats[207]. While we did not directly quantify changes in tissue mass, the 1-week induction of these transcripts in males could be explained by transient training-induced hypertrophy of the adrenal gland. Genes in this transcript set were related to steroid hormone synthesis pathways and mitochondrial function (Supplementary Table 11). Indeed, differential expression of many mitochondria-associated genes could be explained by changes in markers of mitochondrial volume[35]. These results suggest that the adrenal gland undergoes modifications related to its major functions in production and release of steroid hormones and catecholamines in response to endurance training. Finally, motifs of 14 TFs were found to be enriched in this transcript set, including the metabolism-regulating factors

PPARg, PPARa, and ERRg (Supplementary Table 17). PPARs are known regulators of lipid metabolism in response to endurance training[173], and ERRg regulates mitochondrial metabolism in both cardiac[208] and skeletal muscles[209]. Altogether, the adrenal gland shows several sex-specific endurance training adaptations at the molecular level, which should be interpreted with known sex- and strain-specific differences in the hypothalamo-pituitary-adrenal axis function in mind[210].

Immune-related transcriptional changes in the small intestine

A set of small intestine transcripts down-regulated at 8 weeks, with a more robust response in females, was significantly enriched for pathways related to gut inflammation and positively correlated with markers of several immune cell types (Supplementary Table 11; Fig. 5b-c). This result suggests a greater decrease in immune cell type populations in the small intestine of female rats and a sex-dependent change in gut immunity. Specifically, reduced expression of transcripts in the small intestine of female rats included genes involved in B cell receptor signaling activation (*Btk*, *Cd72*, *Cd79a*) and B and T cell recruitment and activation (*Cd3e*, *Gata3*, *Il2rg*, *Ptpn22*, *Zap70*, *Cd5*, *Card11*) (Supplementary Table 11). The pathogenesis of IBD and other autoimmune disorders is associated with reduced or altered microbial diversity, inflammation, and reduced gut barrier integrity, which lead to systemic inflammation and reduced immune tolerance[211–214]. Endurance training is suggested to reduce systemic inflammation, in part by increasing gut microbial diversity and gut barrier integrity[29]. We highlighted the decreases in *Cxcr3* and *Il1a* with training (Extended Data Fig 12g), both implicated in the pathogenesis of IBD[215–218]. CXCR3 increases small intestinal permeability[30]; reduced expression of Il1a is indicative of improvements in gut tissue homeostasis[31] and promotes localized immune cell recruitment[215,219]. Consistent with reduced overall gut inflammation, the expression of transcripts involved in superoxide production in the small intestine (*Rac2*, *Ncf1*/*Ncf4*, *Cybb*)[220,221] were decreased with endurance training (Supplementary Table 2). Reactive oxygen species increase intestinal permeability and propagate gut-mediated systemic inflammation[222,223].

Multi-tissue changes in lipids

We summarized training-regulated metabolites across tissues by performing enrichment of RefMet metabolite classes (Fig. 6a; Supplementary Table 26). This analysis focused primarily on lipid classes, as 2105 out of the total 2430 annotated metabolite features in our data were classified as lipids (Supplementary Table 21). The liver showed the greatest number of significantly enriched classes, followed by the heart, lung, and hippocampus. The eicosanoid and octadecanoid lipid classes had significant enrichments in 8 and 7 tissues, respectively. Eicosanoids and octadecanoids are derived from 20- and 18-carbon polyunsaturated fatty acids, respectively, most frequently arachidonic acid (AA)[224]. The AA pathway plays a key role as an inflammatory mediator involved in many molecular and cellular functions under different physiological conditions[225]. Fatty acids and fatty esters, which serve as both energy sources and building blocks for cellular membranes, were enriched in 7 tissues. Within fatty esters,

acylcarnitines showed unique regulation across the plasma, brown and white adipose tissues, heart, and liver (Extended Data Fig. 13e). An early increase in medium- and long-chain acylcarnitines occurred in the plasma, white adipose tissue, and liver, while a decrease in short-chain acylcarnitines occurred in brown adipose tissue and the heart. Several metabolites showed similar regulation across many tissues suggesting organism-level changes, including increases in N-methylproline and ectoine and decreases in trimethylamine-N-oxide (Extended Data Fig. 13b). Together, these analyses reveal the dynamic mobilization and oxidation of lipids across many tissues in response to endurance training.

Conclusions

Mapping the molecular responses to endurance training in a whole organism is critical for gaining a holistic understanding of the mechanisms that underlie the benefits of exercise. Previous studies have provided valuable findings but are restricted in scope because they used limited molecular platforms, examined few tissues, interrogated a narrow temporal range, or were biased towards a single sex. As large-scale publicly available omic data have proven to be indispensable for rapidly advancing biomedical research, e.g, [78,148,226,227], we expect that our new resource will similarly accelerate the generation of novel hypotheses pertaining to the molecular basis of endurance training.

Each omic dataset quantifies molecules with particular biochemical characteristics and provides unique insights into exercise adaptation. However, none alone are expected to provide a complete picture[228,229]. Through multi-omic analyses we aimed to develop a holistic understanding of a complex physiological response. Representative examples of these insights were through transcriptomics and proteomics analyses that showed that most tissues have persistent protein changes with transient transcript responses, in agreement with models of tissue training adaptation[173]. However, in some cases transcript changes persist 48 hours after the last bout of exercise, showing agreement with the cognate protein, for example energy metabolism in skeletal muscles. Multi-omic integration also enhances mapping of the signaling mechanisms of adaptation; proteomics data revealed changes in the heart extracellular matrix (ECM), and through corresponding prediction of kinase activity from phosphoproteomics data, we were able to propose an underlying signaling mechanism via SRC kinase. Moreover, the regulation of kinase activity in the lung may be associated with lung mechanical stress[230,231]. Integrating metabolomics data with other omes helped explain the mechanics behind metabolic regulation, for example the association between liver lipids and the acetylation of proteins in specific pathways. Some tissues were not subjected to comprehensive multi-omic profiling, yet the discoveries from this work will motivate their deep molecular profiling in the future, such as the observed transcriptional regulation of inflammatory bowel disease genes in the small intestine.

The sex-biased responses to exercise training have not been well characterized. We observed sex differences in the training response across many tissues and omes, highlighting the critical importance of including both sexes in exercise science research. Though we recognize that sexual dimorphism is substantially greater in rats than in humans[232] and we have a limited

sample size for sex-specific comparisons, this work identified important sex-specific adaptive processes in the adrenal gland, lung, and brown and white adipose tissues, with implications for how endurance training may differentially improve health in males and females.

We note limitations in our experimental design, datasets, and analyses. In short, samples were collected 48 hours after the last training bout, therefore acute exercise changes are missed. The experiment was designed to capture persistent molecular changes in response to endurance exercise training, and as such, samples were collected 48 hours after the last training bout. Transient molecular changes induced by acute exercise that occur in minutes to hours after the training session, primarily in mRNA transcripts and metabolites, are likely not captured through this experimental design. Future studies integrating acute temporal responses to repeated bouts of endurance exercise will be required to address this question. Our assays were performed on bulk tissue and do not cover single-cell platforms. Our resource has limited omic characterization for certain tissues, and some omes with emerging biological relevance were not performed in this study, for example, microbiome profiling. Additionally, hundreds of unannotated metabolomic features remain to be explored and analyzed in detail. While the fold-changes of many training-regulated features associated with tissue remodeling during endurance exercise training are modest, they represent coherent processes. We analyzed 3-6 animals per time point and sex combination, and this sample size may limit our ability to identify modest yet physiologically relevant molecular alterations. The findings and associations we describe are from rats and may not translate directly to humans. Moreover, our results are hypothesis-generating and require biological validation. Towards this end, we have generated a large tissue bank from this study for both sexes and all training timepoints. A key mission of MoTrPAC was to make these biospecimens available to the research community to help facilitate new research on the benefits of exercise and health.

AUTHOR CONTRIBUTIONS

Joshua Adkins: Led the Proteomics Chemical Analysis Site at PNNL; collaborated with the consortium to generate the experimental design; oversaw the experimental design and implementation of proteomics workflows; reviewed data analysis platform for proteomics and proteomics interpretation; reviewed the manuscript and made suggestions for edits

Jose Juan Almagro Armenteros: Contributed to the immunological analysis of the small intestine and interpretation of the results. Contribute to the immune figure.

David Amar: Co-led the manuscript working group; compiled analysis resources and documentation. Led the statistical and computational design and analysis throughout the paper. Contributed to RNA-seq, RRBS, proteomics, and metabolomics quantification, normalization pipelines, and optimization of all differential analysis pipelines. Performed the metabolomics meta-analysis; repfdr-based Bayesian clustering of the differential analysis results; network biology analyses and tissue comparisons. Helped write, distribute, and revise the manuscript.

Mary Anne Amper: Processed GET assays, data generation, and QC.

Julian Avila-Pacheco: Performed metabolomic data QC, data preparation, analyses, and compound ID harmonization across sites. Contributed in the discussion of metabolomic related analyses in the manuscript and global tally of project samples used for each data generation platform.

Nasim Bararpour: Contributed to metabolome-related data discussion & interpretation. Contributed to supplementary immune figure panel

Bryan C Bergman: Contributed to design of study, reviewed manuscript as part of publications committee.

Sue C. Bodine: Co-lead preclinical animal study site: Contributed to the development of the animal protocol and design of the study; oversaw the implementation of the animal training protocol, participated in tissue collections and data analysis, contributed to the writing and editing of the manuscript

Frank W Booth: Contributed to data interpretation and reading and editing manuscript

Thomas Buford: Contributed to study analytic discussions; reviewed the manuscript and made suggestions for edits

Toby Chambers: Contributed to the design of the study; participated in data analysis calls, reviewed the manuscript

Clarisa Chavez: Contributed to the immunoassay literature review

Natalie M. Clark: Contributed to additional analyses, figure and manuscript preparation as part of the revision process.

Paul M. Coen: Contributed to the design of the study; participated in data analysis calls.

Elaine Cornell: University of Vermont Biorepository Senior Supervisor; oversaw all administrative aspects of the Biorepository, including MoTrPAC

Gary Cutter: Contributed to comments on analysis of results

Surendra Dasari: Processed the targeted metabolomics data.

Luis G. O. De Sousa: Assisted in animal endurance training protocols, tissue collection, data acquisition, and data entry.

Courtney Dennis: Sample processing, data generation, and QC of LC-MS metabolomics data.

Karyn A. Esser: Co-lead preclinical animal study site: Contributed to the development of the animal protocol and design of the study; helped revise and edit the manuscript

Charles Evans: Led development of cross-site MoTrPAC metabolomics method of procedure (MOP) document, prepared site-specific standard operating procedure (SOP) documents including developing and validating protocols for internal standard controlled metabolite extraction, and supervised use of these protocols to prepare tissue and plasma samples and perform untargeted metabolomics analysis

Facundo M. Fernandez: Contributed to the design and implementation of the overall lipidomics measurement approach. Led the CAS team at the Georgia Institute of Technology involved in non-targeted lipidomics measurements and lipid annotation using high resolution mass spectrometry. Conceived data dictionary approach for lipid annotation. Reviewed the manuscript and made edits.

Nicole Gagne: Biorepository technician; worked closely with Sandra May on all technical aspects of the Biorepository

David A. Gaul: Implemented non-targeted lipidomics measurement platform for a variety of tissues. Curated data dictionary for lipid annotation. Quality oversight of lipidomics datasets.

Nicole R. Gay: Co-led the manuscript working group and figure subgroups; compiled analysis resources and documentation. Analyzed RNA-seq and ATAC-seq pilot data. Contributed to RNA-seq, ATAC-seq, immunoassay, and metabolomics QC, quantification, normalization, and/or differential analysis pipelines. Performed pathway enrichment of graphical clusters; immune cell type correlation or enrichment analyses; analyses related to global, adrenal, lung, and adipose sex differences; and some multi-tissue analyses. Helped write and distribute the manuscript. Developed the MotrpacRatTraining6mo and MotrpacRatTraining6moData R packages and the tissue-level multi-omic graphical analysis reports. Coordinated and contributed to revisions.

Yongchao Ge: Developed the snakemake pipelines and the method of procedures (MOP) for rna-seq and rrbs data data processing and the QC.

Bret H. Goodpaster: Contributed to the design of the study; reviewed the manuscript and made suggestions for edits

Laurie J Goodyear: One of the lead preclinical animal study site Investigators. Contributed to the development of the animal protocol and design of the study; discussion and development of data.

Josh Hansen: Processed proteomic samples at PNNL

Andrea L. Hevener: Contributed to data interpretation, relevant citations, and review of the manuscript

Fang-Chi Hsu: Participated on data analysis calls, reviewed the manuscript and made suggestions for edits

Kim M. Huffman: Contributed to the design of the study; reviewed the manuscript and made suggestions for edits

Olga Ilkayeva: Led the Duke Metabolomics team that prepared samples and performed the targeted mass spec analysis across five tissue types.

Bailey E. Jackson: Assisted in animal endurance training protocols; tissue collection, data acquisition, and data entry.

Pierre M Jean Beltran: Co-led the manuscript writing and data analysis working groups. Led the analysis and biological interpretation of proteomics data and integration with other omics. Contributed to design and development of proteomics and metabolomics data processing, analysis, and visualization pipelines. Co-led the study design for proteomics, including sample processing, and data generation at the Broad Institute Proteomics Platform.

David Jimenez-Morales: Led the development of the data transfer guidelines. Developed the MotrpacBicQC package for QA/QC metabolomics and proteomics submissions. Map PTMs from Rat to Human proteins. Developed the cloud implementation of the proteomics data analysis pipeline. Contributed to metabolomics and proteomics normalization and differential analysis pipelines. Coordinated and performed data preparation and releases. Co-supervised BIC staff and trainees. Co-chair the manuscript working group calls. Reviewed the manuscript and made suggestions for edits. Provided extensive input and feedback on the MoTrPAC Data Hub design and functionality. Coordinated the release of public source code repositories

Christopher Jin: Contributed to the analysis/literature review of the Cytokine portion of the manuscript

Maureen Kachman: Led the Michigan MoTrPAC CAS team that prepared and analyzed samples on three Untargeted mass spectrometry platforms, which provided semiquantitative data for both unannotated and annotated metabolites across tissue types

Benjamin G. Ke: Contributed to data analysis; reviewed the manuscript

Hasmik Keshishian: Co-led the study including study design for proteomics, overseeing sample processing, data generation and analysis at the Broad Proteomics site. Reviewed the manuscript and suggested edits

Kyle S. Kramer: Assisted in animal endurance training protocols; tissue collection, data acquisition, data entry, determined training protocol adjustments when necessary.

William E. Kraus: Contributed to the design of the study; reviewed the manuscript and made suggestions for edits

Christiaan Leeuwenburgh: Contributed to the development of the initial animal protocol and design of the study

Bridget Lester: Contributed to the design of the study

Malene E. Lindholm: Contributed to study design discussions, co-led the sex differences figure subgroup, performed analyses for main and supplemental figure panels, biological data interpretation, helped write and revise the manuscript, advised motrpac-datahub development and data repository

Ana Lira: Assisted in animal endurance training protocol, data acquisition, tissue collection, and data entry.

Xueyun Liu: Contributed to lipidomics sample prep, data collections, and data analysis at Emory University.

Kristal Maner-Smith: Contributed to data interpretation and reading and editing manuscript at Emory University

Gina M Many: Contributed to data analysis and interpretation, and writing the manuscript, including revising the manuscript for additional biological interpretation

Shruti Marwaha: Contributed to immunoassay QC

Sandra May: Biorepository senior technician; oversaw the development and implementation of all technical aspects of the Biorepository including kit design, sample receipt, cryopulverization, aliquot preparation, etc.

Michael E. Miller: Contributed to the design of the study; reviewed the manuscript and made suggestions for edits

Stephen B. Montgomery: Contributed to the design of study, co-PI of Stanford GET team, co-chair of publications committee, contributed to writing and revision of manuscript

Ronald J Moore: Contributed to LC-MS methods development and data acquisition at PNNL

Samuel G Moore: Performed lipidomics sample prep, data collection, and data analysis at Georgia Tech.

Kerrie L. Moreau: Contributed to the design of the study; reviewed the manuscript and made suggestions for edits

Michael Muehlbauer: Formatted and downloaded data from the Duke Metabolomics team for the MoTrPAC BIC database.

Charles C. Mundorff: Sample processing and data analysis contributions at Broad Proteomics site

Nicolas Musi: Participated on data analysis calls, reviewed the manuscript and made suggestions for edits

Venugopalan D. Nair: Co-led the Mount Sinai GET team. Oversaw assay development and implementation of GET assays, data generation, and QC.

Archana Natarajan Raja: Contributed to the development of cloud based pipelines for the analysis of epigenomics and transcriptomics data in collaboration with Sinai and Stanford GET CAS sites. Processed rna-seq, atac-seq and rrbs datasets through these standardized cloud based pipelines. Contributed to QC of the data, standardized data submission and ingestion.

Michael Nestor: Contributed to data analysis pipeline

Eric A. Ortlund: Contributed to the design and implementation of the overall targeted lipidomics measurement approach. Led the CAS team at Emory University involved in targeted lipidomics measurements using mass spectrometry and implementation of bioinformatics analyses for both targeted and untargeted data. Reviewed the manuscript and made edits.

Marco Pahor: Contributed to the design of the study; reviewed the manuscript

Paul Piehowski: Study design and oversight of data production for proteomics at PNNL

Wei-Jun Qian: Co-led the Proteomics Chemical Analysis Site at PNNL; oversaw the experimental design and implementation of proteomics workflows; reviewed the manuscript and made suggestions for edits

Megan E. Ramaker: Participated on data analysis calls, reviewed the manuscript and made suggestions for edits

Alexander Raskind: Initial processing of untargeted metabolomics data and IT support for MoTrPAC data management by the Michigan CAS

R. Scott Rector: Contributed to data interpretation and reading and editing manuscript

Collyn Z-T. Richards: Assisted in animal endurance training protocols; tissue collection, data acquisition, and data entry.

Jessica L. Rooney: Biorepository MoTrPAC Project Manager; directly responsible for all logistic (kits, labels, etc) and technical (sample distribution design, etc) aspects of the Biorepository

Frederique Ruf-Zamojski: Contributed to the development of experimental protocols and quality control assessment of the data, reviewed the manuscript

Scott Rushing: Oversight of the programming of data collection, performed data transfer to Bioinformatics core, reviewed the manuscript and made suggestions for edits

Tyler J. Sagendorf: Contributed R code to generate enrichment heatmaps

James A. Sanford: Study design and oversight of data production for proteomics at PNNL; contributed to the development of proteomics data analysis pipeline and interpretation; co-led the immune/inflammatory figure subgroup; participated in figure generation, manuscript writing and revision.

Simon Schenk: Co-investigator for preclinical animal study site: Contributed to the development of the animal protocol and design of the study; contributed to data interpretation, and the writing, editing and revising of the manuscript

Stuart C. Sealfon: Co-led a data generation site. Writing group member. Revised the manuscript.

Nitish Seenarine: Performed and processed GET assays, data generation, and QC as a member of the Mount Sinai GET team. Developed automated Methyl Capture protocol for use

Gregory R. Smith: Co-led the sex differences figure subgroup, aided with design of analysis plans, conducted transcription factor motif enrichment analysis, studied cross-tissue sex differences, and helped write the manuscript

Kevin S. Smith: Stanford GET Project Manager

Tanu Soni: Processed Michigan metabolomics data QC and normalization.

Alec Steep: Processed metabolomics datasets comprehensively and generated official metabolomics data freezes. Performed and developed QC, cross-technology correlation, and differential analyses to guide metabolomics data processing decisions.

Cynthia L. Stowe: Contributed to the design of the study, developed manual of procedures, oversaw the development of data collection forms, performed quality control of data, reviewed the manuscript and made suggestions for edits

Yifei Sun: Contributed data analysis and data generation. Reviewed protocols used in manuscript

John Thyfault: Contributed to interpreting and development of the data and editing the manuscript

Russell Tracy: Biorepository Director; as Vice-Chair of the Steering Committee, contributed to overall study design, and all aspects of sample handling and distribution to analytical sites

Scott Trappe: Contributed to the design of the study, participated in data analysis calls, reviewed the manuscript

Todd Trappe: Contributed to the design of the study, participated in data analysis calls, reviewed the manuscript

Mital Vasoya: Processed samples and QC for epigenetic data generation

Nikolai G. Vetr: Performed analyses for and generated multiple iterations of main and supplemental figure panel drafts, contributed final figure panels, provided advice and implementation details for multiple additional panels, wrote corresponding manuscript text

Michael P. Walkup: Performed all rat randomizations, performed quality control of data, reviewed the manuscript and made suggestions for edits

Martin J. Walsh: Co-led a data generation site. Reviewed manuscript and made minor suggestions for edits.

Matthew T. Wheeler: Co-led the Bioinformatics Center. Contributed to design, QC and data analysis. Supervised BIC staff and trainees. Oversaw motrpac-datahub development and data repository. Reviewed the manuscript and made suggestions for edits.

Si Wu: Contribute to metabolic-centered figure and discussion, molecule clustering analysis exploration

Ashley Xia: Contributed to the design of the study, planning of omics assay and data management; reviewed the manuscript and made suggestions for edits

Zhen Yan: Contribute to the editing of the manuscript

Chongzhi Zang: Contributed to data analysis; reviewed the manuscript

Elena Zaslavsky: Contributed to the development of GET QC data standards and processing pipelines, reviewed the manuscript

Tiantian Zhang: Contributed to the Emory Cas Site QC analysis, data processing and performed metabolite chemical class enrichment analysis of landscape paper

Bingqing Zhao: Led or co-led the Stanford GET team on protocol optimization, method development, automation and data generation of ATAC-seq, RNA-seq and immunoassays. Co-led analysis of ATAC-seq and immunoassays. Co-led immuno figure subgroup. Contributed to manuscript writing.

Jimmy Zhen: Contributed to development of MoTrPAC Data Hub and cloud computing infrastructure; contributed to design of figure in the manuscript.

REFERENCES

47. Shavlakadze, T. *et al.* Age-Related Gene Expression Signature in Rats Demonstrate Early, Late, and Linear Transcriptional Changes from Multiple Tissues. *Cell Rep.* **28**, 3263-3273.e3 (2019).

48. Wisløff, U., Helgerud, J., Kemi, O. J. & Ellingsen, O. Intensity-controlled treadmill running in rats: VO(2 max) and cardiac hypertrophy. *Am. J. Physiol. Heart Circ. Physiol.* **280**, H1301-1310 (2001).

49. Meissner, A. *et al.* Reduced representation bisulfite sequencing for comparative high-resolution DNA methylation analysis. *Nucleic Acids Res.* **33**, 5868–5877 (2005).

50. Andrews, S. FastQC: A Quality Control Tool for High Throughput Sequence Data. https://qubeshub.org/resources/fastqc (2015).

51. Dobin, A. *et al.* STAR: ultrafast universal RNA-seq aligner. *Bioinforma. Oxf. Engl.* **29**, 15–21 (2013).

52. Langmead, B. & Salzberg, S. L. Fast gapped-read alignment with Bowtie 2. *Nat. Methods* **9**, 357–359 (2012).

53. Li, H. *et al.* The Sequence Alignment/Map format and SAMtools. *Bioinformatics* **25**, 2078–2079 (2009).

54. Ewels, P., Magnusson, M., Lundin, S. & Käller, M. MultiQC: summarize analysis results for multiple tools and samples in a single report. *Bioinforma. Oxf. Engl.* **32**, 3047–3048 (2016).

55. Jäger, M. L. MCL: Markov Cluster Algorithm. https://CRAN.R-project.org/package=MCL (2015).

56. Robinson, M. D., McCarthy, D. J. & Smyth, G. K. edgeR: a Bioconductor package for differential expression analysis of digital gene expression data. *Bioinforma. Oxf. Engl.* **26**, 139–140 (2010).

57. Bolstad, B. preprocessCore: A collection of pre-processing functions. *GitHub*

https://github.com/bmbolstad/preprocessCore (2021).

58. Buenrostro, J., Wu, B., Chang, H. & Greenleaf, W. ATAC-seq: A Method for Assaying Chromatin Accessibility Genome-Wide. *Curr. Protoc. Mol. Biol. Ed. Frederick M Ausubel Al* **109**, 21.29.1-21.29.9 (2015).

59. Corces, M. R. *et al.* An improved ATAC-seq protocol reduces background and enables interrogation of frozen tissues. *Nat. Methods* **14**, 959–962 (2017).

60. Moore, J. E. *et al.* Expanded encyclopaedias of DNA elements in the human and mouse genomes. *Nature* **583**, 699–710 (2020).

61. Martin, M. Cutadapt removes adapter sequences from high-throughput sequencing reads. *EMBnet.journal* **17**, 10–12 (2011).

62. Gaspar, J. M. Improved peak-calling with MACS2. 496521 Preprint at https://doi.org/10.1101/496521 (2018).

63. Li, Q., Brown, J. B., Huang, H. & Bickel, P. J. Measuring reproducibility of high-throughput experiments. *Ann. Appl. Stat.* **5**, 1752–1779 (2011).

64. Quinlan, A. R. & Hall, I. M. BEDTools: a flexible suite of utilities for comparing genomic features. *Bioinforma. Oxf. Engl.* **26**, 841–842 (2010).

65. Law, C. W., Chen, Y., Shi, W. & Smyth, G. K. voom: Precision weights unlock linear model analysis tools for RNA-seq read counts. *Genome Biol.* **15**, R29 (2014).

66. Li, B. & Dewey, C. N. RSEM: accurate transcript quantification from RNA-Seq data with or without a reference genome. *BMC Bioinformatics* **12**, 323 (2011).

67. Broad Institute. Picard Toolkit. *GitHub* https://broadinstitute.github.io/picard/ (2019) doi:10.1101/gr.229102.

68. Love, M. I., Huber, W. & Anders, S. Moderated estimation of fold change and dispersion for RNA-seq data with DESeq2. *Genome Biol.* **15**, (2014).

69. Mertins, P. *et al.* Reproducible workflow for multiplexed deep-scale proteome and phosphoproteome analysis of tumor tissues by liquid chromatography-mass spectrometry.

Nat. Protoc. **13**, 1632–1661 (2018).

70. Zecha, J. *et al.* TMT Labeling for the Masses: A Robust and Cost-efficient, In-solution Labeling Approach. *Mol. Cell. Proteomics MCP* **18**, 1468–1478 (2019).

71. Keshishian, H. *et al.* A highly multiplexed quantitative phosphosite assay for biology and preclinical studies. *Mol. Syst. Biol.* **17**, e10156 (2021).

72. Udeshi, N. D. *et al.* Rapid and deep-scale ubiquitylation profiling for biology and translational research. *Nat. Commun.* **11**, 359 (2020).

73. Myers, S. A. *et al.* Evaluation of Advanced Precursor Determination for Tandem Mass Tag (TMT)-Based Quantitative Proteomics across Instrument Platforms. *J. Proteome Res.* **18**, 542–547 (2019).

74. Kim, S. & Pevzner, P. A. MS-GF+ makes progress towards a universal database search tool for proteomics. *Nat. Commun.* **5**, 5277 (2014).

75. Beausoleil, S. A., Villén, J., Gerber, S. A., Rush, J. & Gygi, S. P. A probability-based approach for high-throughput protein phosphorylation analysis and site localization. *Nat. Biotechnol.* **24**, 1285–1292 (2006).

76. Zhang, B., Chambers, M. C. & Tabb, D. L. Proteomic Parsimony through Bipartite Graph Analysis Improves Accuracy and Transparency. *J. Proteome Res.* **6**, 3549–3557 (2007).

77. Monroe, M. E., Shaw, J. L., Daly, D. S., Adkins, J. N. & Smith, R. D. MASIC: a software program for fast quantitation and flexible visualization of chromatographic profiles from detected LC-MS(/MS) features. *Comput. Biol. Chem.* **32**, 215–217 (2008).

78. Satpathy, S. *et al.* A proteogenomic portrait of lung squamous cell carcinoma. *Cell* **184**, 4348-4371.e40 (2021).

79. Gillette, M. A. *et al.* Proteogenomic Characterization Reveals Therapeutic Vulnerabilities in Lung Adenocarcinoma. *Cell* **182**, 200-225.e35 (2020).

80. Swatek, K. N. & Komander, D. Ubiquitin modifications. *Cell Res.* **26**, 399–422 (2016).

81. Koelmel, J. P. *et al.* Expanding Lipidome Coverage Using LC-MS/MS Data-Dependent

Acquisition with Automated Exclusion List Generation. *J. Am. Soc. Mass Spectrom.* **28**, 908–917 (2017).

82. Anderson, B. G., Raskind, A., Habra, H., Kennedy, R. T. & Evans, C. R. Modifying Chromatography Conditions for Improved Unknown Feature Identification in Untargeted Metabolomics. *Anal. Chem.* **93**, 15840–15849 (2021).

83. Kachman, M. *et al.* Deep annotation of untargeted LC-MS metabolomics data with Binner. *Bioinforma. Oxf. Engl.* **36**, 1801–1806 (2020).

84. Fan, S. *et al.* Systematic Error Removal Using Random Forest for Normalizing Large-Scale Untargeted Lipidomics Data. *Anal. Chem.* **91**, 3590–3596 (2019).

85. Sumner, L. W. *et al.* Proposed minimum reporting standards for chemical analysis Chemical Analysis Working Group (CAWG) Metabolomics Standards Initiative (MSI). *Metabolomics Off. J. Metabolomic Soc.* **3**, 211–221 (2007).

86. Dunn, W. B. *et al.* Procedures for large-scale metabolic profiling of serum and plasma using gas chromatography and liquid chromatography coupled to mass spectrometry. *Nat. Protoc.* **6**, 1060–1083 (2011).

87. Fahy, E. *et al.* A comprehensive classification system for lipids. *J. Lipid Res.* **46**, 839–861 (2005).

88. Fahy, E. *et al.* Update of the LIPID MAPS comprehensive classification system for lipids. *J. Lipid Res.* **50 Suppl**, S9-14 (2009).

89. Kwee, L. C. *et al.* Metabolites and diabetes remission after weight loss. *Nutr. Diabetes* **11**, 10 (2021).

90. Olson, K. C., Chen, G. & Lynch, C. J. Quantification of branched-chain keto acids in tissue by ultra fast liquid chromatography-mass spectrometry. *Anal. Biochem.* **439**, 116–122 (2013).

91. Deutsch, J., Grange, E., Rapoport, S. I. & Purdon, A. D. Isolation and quantitation of long-chain acyl-coenzyme A esters in brain tissue by solid-phase extraction. *Anal.*

Biochem. **220**, 321–323 (1994).

92. Magnes, C., Sinner, F. M., Regittnig, W. & Pieber, T. R. LC/MS/MS method for quantitative determination of long-chain fatty acyl-CoAs. *Anal. Chem.* **77**, 2889–2894 (2005).

93. Minkler, P. E., Kerner, J., Ingalls, S. T. & Hoppel, C. L. Novel isolation procedure for short-, medium-, and long-chain acyl-coenzyme A esters from tissue. *Anal. Biochem.* **376**, 275–276 (2008).

94. Yenilmez, B. *et al.* Paradoxical activation of transcription factor SREBP1c and de novo lipogenesis by hepatocyte-selective ATP-citrate lyase depletion in obese mice. *J. Biol. Chem.* **298**, 102401 (2022).

95. Cordell, R. L., Hill, S. J., Ortori, C. A. & Barrett, D. A. Quantitative profiling of nucleotides and related phosphate-containing metabolites in cultured mammalian cells by liquid chromatography tandem electrospray mass spectrometry. *J. Chromatogr. B Analyt. Technol. Biomed. Life. Sci.* **871**, 115–124 (2008).

96. Gooding, J. R. *et al.* Adenylosuccinate Is an Insulin Secretagogue Derived from Glucose-Induced Purine Metabolism. *Cell Rep.* **13**, 157–167 (2015).

97. Gonsalves, W. I. *et al.* Metabolomic and Lipidomic Profiling of Bone Marrow Plasma Differentiates Patients with Monoclonal Gammopathy of Undetermined Significance from Multiple Myeloma. *Sci. Rep.* **10**, 10250 (2020).

98. Lanza, I. R. *et al.* Quantitative Metabolomics by 1H-NMR and LC-MS/MS Confirms Altered Metabolic Pathways in Diabetes. *PLOS ONE* **5**, e10538 (2010).

99. Dutta, T. *et al.* Impact of Long-Term Poor and Good Glycemic Control on Metabolomics Alterations in Type 1 Diabetic People. *J. Clin. Endocrinol. Metab.* **101**, 1023–1033 (2016).

100. Wilkins, J., Sakrikar, D., Petterson, X.-M., Lanza, I. R. & Trushina, E. A comprehensive protocol for multiplatform metabolomics analysis in patient-derived skin fibroblasts. *Metabolomics Off. J. Metabolomic Soc.* **15**, 83 (2019).

101. Blachnio-Zabielska, A. U., Persson, X.-M. T., Koutsari, C., Zabielski, P. & Jensen, M. D. A

liquid chromatography/tandem mass spectrometry method for measuring the in vivo incorporation of plasma free fatty acids into intramyocellular ceramides in humans. *Rapid Commun. Mass Spectrom. RCM* **26**, 1134–1140 (2012).

102. Savica, R. *et al.* Plasma sphingolipid changes with autopsy-confirmed Lewy Body or Alzheimer's pathology. *Alzheimers Dement. Amst. Neth.* **3**, 43–50 (2016).

103. Chace, D. H. *et al.* Electrospray Tandem Mass Spectrometry for Analysis of Acylcarnitines in Dried Postmortem Blood Specimens Collected at Autopsy from Infants with Unexplained Cause of Death. *Clin. Chem.* **47**, 1166–1182 (2001).

104. Collins, J. M. *et al.* TCA cycle remodeling drives proinflammatory signaling in humans with pulmonary tuberculosis. *PLoS Pathog.* **17**, e1009941 (2021).

105. Maner-Smith, K. M. *et al.* Alterations in the Human Plasma Lipidome in Response to Tularemia Vaccination. *Vaccines* **8**, E414 (2020).

106. Harrell Jr, F. E. *Hmisc: Harrell Miscellaneous*. (2021).

107. Wei, T. & Simko, V. *R Package 'Corrplot': Visualization of a Correlation Matrix*. (2021).

108. Montoya, J. G. *et al.* Cytokine signature associated with disease severity in chronic fatigue syndrome patients. *Proc. Natl. Acad. Sci. U. S. A.* **114**, E7150–E7158 (2017).

109. Ritchie, M. E. *et al.* limma powers differential expression analyses for RNA-sequencing and microarray studies. *Nucleic Acids Res.* **43**, e47 (2015).

110. Chen, Y., Pal, B., Visvader, J. E. & Smyth, G. K. Differential methylation analysis of reduced representation bisulfite sequencing experiments using edgeR. *F1000Research* **6**, 2055 (2017).

111. Hothorn, T., Bretz, F. & Westfall, P. Simultaneous inference in general parametric models. *Biom. J. Biom. Z.* **50**, 346–363 (2008).

112. Zeileis, A. & Hothorn, T. Diagnostic Checking in Regression Relationships. *R News* **2**, 7–10 (2002).

113. Ignatiadis, N., Klaus, B., Zaugg, J. B. & Huber, W. Data-driven hypothesis weighting

increases detection power in genome-scale multiple testing. *Nat. Methods* **13**, 577–580 (2016).

114. Viechtbauer, W. Conducting meta-analyses in R with the metafor package. *J. Stat. Softw.* **36**, 1–48 (2010).

115. Scrucca, L., Fop, M., Murphy, T. B. & Raftery, A. E. mclust 5: clustering, classification and density estimation using Gaussian finite mixture models. *R J.* **8**, 289–317 (2016).

116. Heller, R., Yaacoby, S. & Yekutieli, D. repfdr: a tool for replicability analysis for genome-wide association studies. *Bioinforma. Oxf. Engl.* **30**, 2971–2972 (2014).

117. Heller, R. & Yekutieli, D. Replicability analysis for genome-wide association studies. *Ann. Appl. Stat.* **8**, 481–498 (2014).

118. Efron, B. Size, power and false discovery rates. *Ann. Stat.* **35**, 1351–1377 (2007).

119. Yu, G., Wang, L.-G. & He, Q.-Y. ChIPseeker: an R/Bioconductor package for ChIP peak annotation, comparison and visualization. *Bioinformatics* **31**, 2382–2383 (2015).

120. Raudvere, U. *et al.* g:Profiler: a web server for functional enrichment analysis and conversions of gene lists (2019 update). *Nucleic Acids Res.* **47**, W191–W198 (2019).

121. Picart-Armada, S., Fernández-Albert, F., Vinaixa, M., Yanes, O. & Perera-Lluna, A. FELLA: an R package to enrich metabolomics data. *BMC Bioinformatics* **19**, 538 (2018).

122. Szklarczyk, D. *et al.* The STRING database in 2021: customizable protein-protein networks, and functional characterization of user-uploaded gene/measurement sets. *Nucleic Acids Res.* **49**, D605–D612 (2021).

123. Sales, G., Calura, E., Cavalieri, D. & Romualdi, C. graphite - a Bioconductor package to convert pathway topology to gene network. *BMC Bioinformatics* **13**, 20 (2012).

124. Sales, G., Calura, E. & Romualdi, C. metaGraphite-a new layer of pathway annotation to get metabolite networks. *Bioinforma. Oxf. Engl.* **35**, 1258–1260 (2019).

125. Jassal, B. *et al.* The reactome pathway knowledgebase. *Nucleic Acids Res.* **48**, D498–D503 (2020).

126. Kanehisa, M., Furumichi, M., Sato, Y., Ishiguro-Watanabe, M. & Tanabe, M. KEGG: integrating viruses and cellular organisms. *Nucleic Acids Res.* **49**, D545–D551 (2021).

127. Wishart, D. S. *et al.* PathBank: a comprehensive pathway database for model organisms. *Nucleic Acids Res.* **48**, D470–D478 (2020).

128. Newman, M. E. J. Finding community structure in networks using the eigenvectors of matrices. *Phys. Rev. E Stat. Nonlin. Soft Matter Phys.* **74**, 036104 (2006).

129. Csardi, G. & Nepusz, T. The igraph software package for complex network research. *InterJournal* **Complex Systems**, 1695 (2006).

130. Duttke, S. H., Chang, M. W., Heinz, S. & Benner, C. Identification and dynamic quantification of regulatory elements using total RNA. *Genome Res.* (2019) doi:10.1101/gr.253492.119.

131. Frankish, A. *et al.* GENCODE 2021. *Nucleic Acids Res.* **49**, D916–D923 (2021).

132. Smith, J. R. *et al.* The Year of the Rat: The Rat Genome Database at 20: a multi-species knowledgebase and analysis platform. *Nucleic Acids Res.* **48**, D731–D742 (2020).

133. Krug, K. *et al.* A Curated Resource for Phosphosite-specific Signature Analysis *[S]. *Mol. Cell. Proteomics* **18**, 576–593 (2019).

134. Rath, S. *et al.* MitoCarta3.0: an updated mitochondrial proteome now with sub-organelle localization and pathway annotations. *Nucleic Acids Res.* **49**, D1541–D1547 (2021).

135. Shao, X., Taha, I. N., Clauser, K. R., Gao, Y. T. & Naba, A. MatrisomeDB: the ECM-protein knowledge database. *Nucleic Acids Res.* **48**, D1136–D1144 (2020).

136. Subramanian, A. *et al.* Gene set enrichment analysis: a knowledge-based approach for interpreting genome-wide expression profiles. *Proc. Natl. Acad. Sci. U. S. A.* **102**, 15545–15550 (2005).

137. Altschul, S. F., Gish, W., Miller, W., Myers, E. W. & Lipman, D. J. Basic local alignment search tool. *J. Mol. Biol.* **215**, 403–410 (1990).

138. Camacho, C. *et al.* BLAST+: architecture and applications. *BMC Bioinformatics* **10**, 421

(2009).

139. UniProt Consortium. UniProt: the universal protein knowledgebase in 2021. *Nucleic Acids Res.* **49**, D480–D489 (2021).

140. Scholzen, T. & Gerdes, J. The Ki-67 protein: from the known and the unknown. *J. Cell. Physiol.* **182**, 311–322 (2000).

141. Westcott, G. P. & Rosen, E. D. Crosstalk Between Adipose and Lymphatics in Health and Disease. *Endocrinology* **163**, bqab224 (2022).

142. Steen, C. B., Liu, C. L., Alizadeh, A. A. & Newman, A. M. Profiling Cell Type Abundance and Expression in Bulk Tissues with CIBERSORTx. *Methods Mol. Biol. Clifton NJ* **2117**, 135–157 (2020).

143. Newman, A. M. *et al.* Robust enumeration of cell subsets from tissue expression profiles. *Nat. Methods* **12**, 453–457 (2015).

144. Chen, B., Khodadoust, M. S., Liu, C. L., Newman, A. M. & Alizadeh, A. A. Profiling Tumor Infiltrating Immune Cells with CIBERSORT. *Methods Mol. Biol. Clifton NJ* **1711**, 243–259 (2018).

145. Dougherty, J. D., Schmidt, E. F., Nakajima, M. & Heintz, N. Analytical approaches to RNA profiling data for the identification of genes enriched in specific cells. *Nucleic Acids Res.* **38**, 4218–4230 (2010).

146. Yule, G. U. Why do we Sometimes get Nonsense-Correlations between Time-Series?--A Study in Sampling and the Nature of Time-Series. *J. R. Stat. Soc.* **89**, 1–63 (1926).

147. Borchers, H. W. pracma: Practical Numerical Math Functions. https://CRAN.R-project.org/package=pracma (2022).

148. The GTEx Consortium. The GTEx Consortium atlas of genetic regulatory effects across human tissues. *Science* **369**, 1318–1330 (2020).

149. Lin, M. *et al.* Cell surface antigen CD109 is a novel member of the alpha(2) macroglobulin/C3, C4, C5 family of thioester-containing proteins. *Blood* **99**, 1683–1691

(2002).

150. Kuo, M.-W., Wang, C.-H., Wu, H.-C., Chang, S.-J. & Chuang, Y.-J. Soluble THSD7A is an N-glycoprotein that promotes endothelial cell migration and tube formation in angiogenesis. *PloS One* **6**, e29000 (2011).

151. Li, C. *et al.* CaBP1, a neuronal Ca2+ sensor protein, inhibits inositol trisphosphate receptors by clamping intersubunit interactions. *Proc. Natl. Acad. Sci. U. S. A.* **110**, 8507–8512 (2013).

152. Hakkarainen, J. *et al.* Hydroxysteroid (17β)-dehydrogenase 1-deficient female mice present with normal puberty onset but are severely subfertile due to a defect in luteinization and progesterone production. *FASEB J. Off. Publ. Fed. Am. Soc. Exp. Biol.* **29**, 3806–3816 (2015).

153. Mann, S., Beedie, C. & Jimenez, A. Differential effects of aerobic exercise, resistance training and combined exercise modalities on cholesterol and the lipid profile: review, synthesis and recommendations. *Sports Med. Auckl. NZ* **44**, 211–221 (2014).

154. Lambert, M. *et al.* Loss of KCNK3 is a hallmark of RV hypertrophy/dysfunction associated with pulmonary hypertension. *Cardiovasc. Res.* **114**, 880–893 (2018).

155. Nassal, D. M. *et al.* KChIP2 is a core transcriptional regulator of cardiac excitability. *eLife* **6**, e17304 (2017).

156. Reinstein, E. *et al.* Mutations in TAX1BP3 cause dilated cardiomyopathy with septo-optic dysplasia. *Hum. Mutat.* **36**, 439–442 (2015).

157. Tsun, Z.-Y. *et al.* The folliculin tumor suppressor is a GAP for the RagC/D GTPases that signal amino acid levels to mTORC1. *Mol. Cell* **52**, 495–505 (2013).

158. Toda, H. *et al.* Stem cell-derived neural stem/progenitor cell supporting factor is an autocrine/paracrine survival factor for adult neural stem/progenitor cells. *J. Biol. Chem.* **278**, 35491–35500 (2003).

159. Bartness, T. J., Liu, Y., Shrestha, Y. B. & Ryu, V. Neural innervation of white adipose tissue

and the control of lipolysis. *Front. Neuroendocrinol.* **35**, 473–493 (2014).

160. Muoio, D. M. *et al.* Muscle-specific deletion of carnitine acetyltransferase compromises glucose tolerance and metabolic flexibility. *Cell Metab.* **15**, 764–777 (2012).

161. Megiorni, F., Indovina, P., Mora, B. & Mazzilli, M. C. Minor expression of fascin-1 gene (FSCN1) in NTera2 cells depleted of CREB-binding protein. *Neurosci. Lett.* **381**, 169–174 (2005).

162. Shih, Y.-P., Sun, P., Wang, A. & Lo, S. H. Tensin1 positively regulates RhoA activity through its interaction with DLC1. *Biochim. Biophys. Acta* **1853**, 3258–3265 (2015).

163. Dukay, B., Csoboz, B. & Tóth, M. E. Heat-Shock Proteins in Neuroinflammation. *Front. Pharmacol.* **10**, 920 (2019).

164. Archer, A. E., Von Schulze, A. T. & Geiger, P. C. Exercise, heat shock proteins and insulin resistance. *Philos. Trans. R. Soc. Lond. B. Biol. Sci.* **373**, 20160529 (2018).

165. Liu, Y. *et al.* Human skeletal muscle HSP70 response to training in highly trained rowers. *J. Appl. Physiol. Bethesda Md 1985* **86**, 101–104 (1999).

166. Mancini, A. *et al.* Lifelong Football Training: Effects on Autophagy and Healthy Longevity Promotion. *Front. Physiol.* **10**, 132 (2019).

167. Powers, S. K., Quindry, J. C. & Kavazis, A. N. Exercise-induced cardioprotection against myocardial ischemia-reperfusion injury. *Free Radic. Biol. Med.* **44**, 193–201 (2008).

168. Wogensen, E., Malá, H. & Mogensen, J. The Effects of Exercise on Cognitive Recovery after Acquired Brain Injury in Animal Models: A Systematic Review. *Neural Plast.* **2015**, 830871 (2015).

169. Febbraio, M. A. Exercise metabolism in 2016: Health benefits of exercise — more than meets the eye! *Nat. Rev. Endocrinol.* **13**, 72–74 (2017).

170. Khassaf, M. *et al.* Time course of responses of human skeletal muscle to oxidative stress induced by nondamaging exercise. *J. Appl. Physiol. Bethesda Md 1985* **90**, 1031–1035 (2001).

171. Tilley, A. E., Walters, M. S., Shaykhiev, R. & Crystal, R. G. Cilia dysfunction in lung disease. *Annu. Rev. Physiol.* **77**, 379–406 (2015).

172. de Sousa Abreu, R., Penalva, L. O., Marcotte, E. M. & Vogel, C. Global signatures of protein and mRNA expression levels. *Mol. Biosyst.* **5**, 1512–1526 (2009).

173. Egan, B. & Zierath, J. R. Exercise Metabolism and the Molecular Regulation of Skeletal Muscle Adaptation. *Cell Metab.* **17**, 162–184 (2013).

174. Vogel, C. *et al.* Sequence signatures and mRNA concentration can explain two-thirds of protein abundance variation in a human cell line. *Mol. Syst. Biol.* **6**, 400 (2010).

175. Yang, Y., Creer, A., Jemiolo, B. & Trappe, S. Time course of myogenic and metabolic gene expression in response to acute exercise in human skeletal muscle. *J. Appl. Physiol. Bethesda Md 1985* **98**, 1745–1752 (2005).

176. Sichien, D. *et al.* IRF8 Transcription Factor Controls Survival and Function of Terminally Differentiated Conventional and Plasmacytoid Dendritic Cells, Respectively. *Immunity* **45**, 626–640 (2016).

177. Heddes, M. *et al.* The intestinal clock drives the microbiome to maintain gastrointestinal homeostasis. *Nat. Commun.* **13**, 6068 (2022).

178. Natarajan, A., Wagner, B. & Sibilia, M. The EGF receptor is required for efficient liver regeneration. *Proc. Natl. Acad. Sci. U. S. A.* **104**, 17081–17086 (2007).

179. Yasuda, S. *et al.* Exercise-induced hepatocyte growth factor production in patients after acute myocardial infarction: its relationship to exercise capacity and brain natriuretic peptide levels. *Circ. J. Off. J. Jpn. Circ. Soc.* **68**, 304–307 (2004).

180. Chalasani, N. *et al.* The diagnosis and management of non-alcoholic fatty liver disease: practice Guideline by the American Association for the Study of Liver Diseases, American College of Gastroenterology, and the American Gastroenterological Association. *Hepatol. Baltim. Md* **55**, 2005–2023 (2012).

181. Shephard, R. J. & Johnson, N. Effects of physical activity upon the liver. *Eur. J. Appl.*

Physiol. **115**, 1–46 (2015).

182. Li, N. *et al.* Therapeutic Effect of HGF on NASH Mice Through HGF/c-Met and JAK2-STAT3 Signalling Pathway. *Ann. Hepatol.* **17**, 501–510 (2018).
183. Matsuda, Y., Matsumoto, K., Ichida, T. & Nakamura, T. Hepatocyte growth factor suppresses the onset of liver cirrhosis and abrogates lethal hepatic dysfunction in rats. *J. Biochem. (Tokyo)* **118**, 643–649 (1995).
184. Tahara, M., Matsumoto, K., Nukiwa, T. & Nakamura, T. Hepatocyte growth factor leads to recovery from alcohol-induced fatty liver in rats. *J. Clin. Invest.* **103**, 313–320 (1999).
185. Mann, N. & Rosenzweig, A. Can exercise teach us how to treat heart disease? *Circulation* **126**, 2625–2635 (2012).
186. Coven, D. L. *et al.* Physiological role of AMP-activated protein kinase in the heart: graded activation during exercise. *Am. J. Physiol. Endocrinol. Metab.* **285**, E629-636 (2003).
187. Gong, H., Xie, J., Zhang, N., Yao, L. & Zhang, Y. MEF2A binding to the Glut4 promoter occurs via an AMPKα2-dependent mechanism. *Med. Sci. Sports Exerc.* **43**, 1441–1450 (2011).
188. Hakuno, D., Takahashi, T., Lammerding, J. & Lee, R. T. Focal adhesion kinase signaling regulates cardiogenesis of embryonic stem cells. *J. Biol. Chem.* **280**, 39534–39544 (2005).
189. Sciarretta, S., Forte, M., Frati, G. & Sadoshima, J. New Insights Into the Role of mTOR Signaling in the Cardiovascular System. *Circ. Res.* **122**, 489–505 (2018).
190. Takeishi, Y. *et al.* Src and multiple MAP kinase activation in cardiac hypertrophy and congestive heart failure under chronic pressure-overload: comparison with acute mechanical stretch. *J. Mol. Cell. Cardiol.* **33**, 1637–1648 (2001).
191. Zhai, Y. *et al.* Src-family Protein Tyrosine Kinases: A promising target for treating Cardiovascular Diseases. *Int. J. Med. Sci.* **18**, 1216–1224 (2021).
192. Nakamura, M. & Sadoshima, J. Mechanisms of physiological and pathological cardiac hypertrophy. *Nat. Rev. Cardiol.* **15**, 387–407 (2018).

193. Perrino, C. *et al.* Intermittent pressure overload triggers hypertrophy-independent cardiac dysfunction and vascular rarefaction. *J. Clin. Invest.* **116**, 1547–1560 (2006).

194. Castrén, E. & Rantamäki, T. The role of BDNF and its receptors in depression and antidepressant drug action: Reactivation of developmental plasticity. *Dev. Neurobiol.* **70**, 289–297 (2010).

195. Li, F. & Tsien, J. Z. Memory and the NMDA receptors. *N. Engl. J. Med.* **361**, 302–303 (2009).

196. Erickson, K. I. *et al.* Exercise training increases size of hippocampus and improves memory. *Proc. Natl. Acad. Sci. U. S. A.* **108**, 3017–3022 (2011).

197. Stepniak, E. *et al.* c-Jun/AP-1 controls liver regeneration by repressing p53/p21 and p38 MAPK activity. *Genes Dev.* **20**, 2306–2314 (2006).

198. Takiguchi, M. The C/EBP family of transcription factors in the liver and other organs. *Int. J. Exp. Pathol.* **79**, 369–391 (1998).

199. Lucki, N. C. & Sewer, M. B. Multiple roles for sphingolipids in steroid hormone biosynthesis. *Subcell. Biochem.* **49**, 387–412 (2008).

200. McGee, S. L., Fairlie, E., Garnham, A. P. & Hargreaves, M. Exercise-induced histone modifications in human skeletal muscle. *J. Physiol.* **587**, 5951–5958 (2009).

201. Wang, Z., Qin, G. & Zhao, T. C. HDAC4: mechanism of regulation and biological functions. *Epigenomics* **6**, 139–150 (2014).

202. Niu, Y. *et al.* Exercise-induced GLUT4 transcription via inactivation of HDAC4/5 in mouse skeletal muscle in an AMPKa2-dependent manner. *Biochim. Biophys. Acta BBA - Mol. Basis Dis.* **1863**, 2372–2381 (2017).

203. Luo, L. *et al.* HDAC4 Controls Muscle Homeostasis through Deacetylation of Myosin Heavy Chain, PGC-1α, and Hsc70. *Cell Rep.* **29**, 749-763.e12 (2019).

204. Bauters, D., Scroyen, I., Van Hul, M. & Lijnen, H. R. Gelatinase A (MMP-2) promotes murine adipogenesis. *Biochim. Biophys. Acta* **1850**, 1449–1456 (2015).

205. Palhinha, L. *et al.* Leptin Induces Proadipogenic and Proinflammatory Signaling in Adipocytes. *Front. Endocrinol.* **10**, 841 (2019).

206. Recio-López, P., Valladolid-Acebes, I., Berggren, P.-O. & Juntti-Berggren, L. Apolipoprotein CIII Reduction Protects White Adipose Tissues against Obesity-Induced Inflammation and Insulin Resistance in Mice. *Int. J. Mol. Sci.* **23**, 62 (2021).

207. Stallknecht, B. *et al.* Diminished epinephrine response to hypoglycemia despite enlarged adrenal medulla in trained rats. *Am. J. Physiol.* **259**, R998-1003 (1990).

208. Sakamoto, T. *et al.* A Critical Role for Estrogen-Related Receptor Signaling in Cardiac Maturation. *Circ. Res.* **126**, 1685–1702 (2020).

209. Rangwala, S. M. *et al.* Estrogen-related receptor gamma is a key regulator of muscle mitochondrial activity and oxidative capacity. *J. Biol. Chem.* **285**, 22619–22629 (2010).

210. Chisari, A., Carino, M., Perone, M., Gaillard, R. C. & Spinedi, E. Sex and strain variability in the rat hypothalamo-pituitary-adrenal (HPA) axis function. *J. Endocrinol. Invest.* **18**, 25–33 (1995).

211. Ghouri, Y. A., Tahan, V. & Shen, B. Secondary causes of inflammatory bowel diseases. *World J. Gastroenterol.* **26**, 3998–4017 (2020).

212. Hosseinkhani, F. *et al.* The contribution of gut bacterial metabolites in the human immune signaling pathway of non-communicable diseases. *Gut Microbes* **13**, 1–22 (2021).

213. Lin, L. & Zhang, J. Role of intestinal microbiota and metabolites on gut homeostasis and human diseases. *BMC Immunol.* **18**, 2 (2017).

214. Marchesi, J. R. *et al.* The gut microbiota and host health: a new clinical frontier. *Gut* **65**, 330–339 (2016).

215. Casini-Raggi, V. *et al.* Mucosal imbalance of IL-1 and IL-1 receptor antagonist in inflammatory bowel disease. A novel mechanism of chronic intestinal inflammation. *J. Immunol. Baltim. Md 1950* **154**, 2434–2440 (1995).

216. Jimbo, K. *et al.* Increased expression of CXCR3 axis components and matrix

metalloproteinase in pediatric inflammatory bowel disease patients. *Pediatr. Int. Off. J. Jpn. Pediatr. Soc.* **56**, 873–883 (2014).

217. Schroepf, S. *et al.* Strong overexpression of CXCR3 axis components in childhood inflammatory bowel disease. *Inflamm. Bowel Dis.* **16**, 1882–1890 (2010).

218. Singh, U. P., Venkataraman, C., Singh, R. & Lillard, J. W. CXCR3 axis: role in inflammatory bowel disease and its therapeutic implication. *Endocr. Metab. Immune Disord. Drug Targets* **7**, 111–123 (2007).

219. Chen, C.-J. *et al.* Identification of a key pathway required for the sterile inflammatory response triggered by dying cells. *Nat. Med.* **13**, 851–856 (2007).

220. Carvalho, L. *et al.* Reactive Oxygen Species Deficiency Due to Ncf1-Mutation Leads to Development of Adenocarcinoma and Metabolomic and Lipidomic Remodeling in a New Mouse Model of Dextran Sulfate Sodium-Induced Colitis. *Front. Immunol.* **9**, 701 (2018).

221. Yamauchi, A. *et al.* Rac2-deficient murine macrophages have selective defects in superoxide production and phagocytosis of opsonized particles. *J. Immunol. Baltim. Md 1950* **173**, 5971–5979 (2004).

222. Aviello, G. & Knaus, U. G. ROS in gastrointestinal inflammation: Rescue Or Sabotage? *Br. J. Pharmacol.* **174**, 1704–1718 (2017).

223. Bhattacharyya, A., Chattopadhyay, R., Mitra, S. & Crowe, S. E. Oxidative stress: an essential factor in the pathogenesis of gastrointestinal mucosal diseases. *Physiol. Rev.* **94**, 329–354 (2014).

224. Ortiz, G. G. *et al.* Oxidative Stress: Love and Hate History in Central Nervous System. *Adv. Protein Chem. Struct. Biol.* **108**, 1–31 (2017).

225. Meves, H. Arachidonic acid and ion channels: an update. *Br. J. Pharmacol.* **155**, 4–16 (2008).

226. Corces, M. R. *et al.* The chromatin accessibility landscape of primary human cancers. *Science* **362**, (2018).

227. The ENCODE Project Consortium. An integrated encyclopedia of DNA elements in the human genome. *Nature* **489**, 57–74 (2012).

228. Battle, A. *et al.* Genomic variation. Impact of regulatory variation from RNA to protein. *Science* **347**, 664–667 (2015).

229. Ghazalpour, A. *et al.* Comparative analysis of proteome and transcriptome variation in mouse. *PLoS Genet.* **7**, e1001393 (2011).

230. Gao, L. *et al.* Novel polymorphisms in the myosin light chain kinase gene confer risk for acute lung injury. *Am. J. Respir. Cell Mol. Biol.* **34**, 487–495 (2006).

231. Yang, Y. *et al.* Prognostic value of phospho-Akt in patients with non-small cell lung carcinoma: A meta-analysis. *Int. J. Cancer* **135**, 1417–1424 (2014).

232. Solleveld, H. A., Haseman, J. K. & McConnell, E. E. Natural History of Body Weight Gain, Survival, and Neoplasia in the F344 Rat. *JNCI J. Natl. Cancer Inst.* **72**, 929–940 (1984).
