# Supplementary material for: Temporal dynamics of the multi-omic response to endurance exercise training
Source: Nature. 2024 May 1;629(8010):174–83. doi: 10.1038/s41586-023-06877-w (PMC11062907; doi:10.1038/s41586-023-06877-w)
Supplement: Supplementary file 2 — Reporting Summary [file 41586_2023_6877_MOESM2_ESM.pdf]

nature portfolio

Corresponding author(s): Stephen B. Montgomery, Sue C. Bodine, Steven A. Carr, Karyn A. Esser, Simon Schenk, Michael P. Snyder, Matthew T. Wheeler

Last updated by author(s): Sep 24, 2023

Reporting Summary

Nature Portfolio wishes to improve the reproducibility of the work that we publish. This form provides structure for consistency and transparency in reporting. For further information on Nature Portfolio policies, see our Editorial Policies and the Editorial Policy Checklist.

Statistics

For all statistical analyses, confirm that the following items are present in the figure legend, table legend, main text, or Methods section.

n/a	Confirmed	
☐	☒	The exact sample size (*n*) for each experimental group/condition, given as a discrete number and unit of measurement
☐	☒	A statement on whether measurements were taken from distinct samples or whether the same sample was measured repeatedly
☐	☒	The statistical test(s) used AND whether they are one- or two-sided<br>*Only common tests should be described solely by name; describe more complex techniques in the Methods section.*
☐	☒	A description of all covariates tested
☐	☒	A description of any assumptions or corrections, such as tests of normality and adjustment for multiple comparisons
☐	☒	A full description of the statistical parameters including central tendency (e.g. means) or other basic estimates (e.g. regression coefficient) AND variation (e.g. standard deviation) or associated estimates of uncertainty (e.g. confidence intervals)
☐	☒	For null hypothesis testing, the test statistic (e.g. *F*, *t*, *r*) with confidence intervals, effect sizes, degrees of freedom and *P* value noted<br>*Give P values as exact values whenever suitable.*
☒	☐	For Bayesian analysis, information on the choice of priors and Markov chain Monte Carlo settings
☒	☐	For hierarchical and complex designs, identification of the appropriate level for tests and full reporting of outcomes
☐	☒	Estimates of effect sizes (e.g. Cohen's *d*, Pearson's *r*), indicating how they were calculated

Our web collection on statistics for biologists contains articles on many of the points above.

Software and code

Policy information about availability of computer code

Data collection	The following software were used for the MoTrPAC RNA-seq pipeline (https://github.com/MoTrPAC/motrpac-rna-seq-pipeline, v1.0.0): star (v2.7.0d); cutadapt (v1.18); picard tools (v2.8.16); samtools (v1.3.1); rsem (v1.3.1); multiqc (v1.6); bowtie2 (v2.3.4.3); fastqc (v0.11.8); subread (v1.6.3); ucsc-gtftogenepred (v366). The following software were used for the MoTrPAC RRBS Pipeline (https://github.com/MoTrPAC/motrpac-rrbs-pipeline, v1.1): fastqc (v0.11.8); cutadapt (v1.18); trim_galore (v0.5.0); samtools (v1.3.1); bowtie2 (v2.3.4.3); multiqc (v1.6); bismark (v0.20.0). The ENCODE ATAC-seq pipeline (https://github.com/ENCODE-DCC/atac-seq-pipeline, v1.7.0) was used to process ATAC-seq data; custom post-processing scripts are available at https://github.com/MoTrPAC/motrpac-atac-seq-pipeline. The following software were used for the MoTrPAC proteomics pipeline (https://github.com/MoTrPAC/motrpac-proteomics-pipeline): MASIC (v3.2.7901); MSGFPlus (v2021.09.06); Mzid2Tsv (v1.4.3); PHRP (v1.5.7458); PlexedPiper (v0.3.6); PPMErrorCharter (v1.2.7632); AScore (v1.0.8315). For untargeted metabolomics data processing, the following software were used: TraceFinder (v3.3); Progenesis QI (2021); Profinder (v8.0); Agilent Masshunter Qualitative Analysis (v7.0); Agilent Mass Profiler Pro (v8.0); Masshunter Qualitative Analysis; Binner (v1.0.0); Compound Discoverer (v3.0). For targeted metabolomics data processing, the following software were used: Sciex OS (v1.6.1); TargetLynx (v4.1.1.0); SoftMax Pro (v5.4); Discovery Workbench; NeoLynx (v4.0.6.0); Xcalibur Quant (v4.5.445.18); MassHunter Quant.

Data analysis

QA/QC was performed using the MotrpacBicQC R package (https://github.com/MoTrPAC/MotrpacBicQC/, v0.6.7). Normalization and QC scripts are available at https://github.com/MoTrPAC/MotrpacRatTraining6moQCRep. Code used to perform the main computational analyses presented in the manuscript are provided in the MotrpacRatTraining6mo R package (https://motrpac.github.io/MotrpacRatTraining6mo/, v1.6.4). Specific R package dependencies for this package are available at https://github.com/MoTrPAC/MotrpacRatTraining6mo/blob/main/DESCRIPTION. These dependencies include but are not limited to: DESeq2, edgeR, limma, IHW, multcomp, metafor, repfdr, gprofiler2, igraph, ssGSEA2. Additional R packages used include: mclust, graphite, pSI, pracma, DOSE. The specific version of R and R packages used were analyst-dependent. Additional software used include: HOMER (v4.11.1), BLAST+ (v2.11.0), CIBERSORTx (v1.05).

For manuscripts utilizing custom algorithms or software that are central to the research but not yet described in published literature, software must be made available to editors and reviewers. We strongly encourage code deposition in a community repository (e.g. GitHub). See the Nature Portfolio guidelines for submitting code & software for further information.

Data

Policy information about availability of data

All manuscripts must include a data availability statement. This statement should provide the following information, where applicable:

- Accession codes, unique identifiers, or web links for publicly available datasets
- A description of any restrictions on data availability
- For clinical datasets or third party data, please ensure that the statement adheres to our policy

MoTrPAC data are publicly available via http://motrpac-data.org/data-access. Data access inquiries should be sent to motrpac-helpdesk@lists.stanford.edu. Additional resources can be found at http://motrpac.org and https://motrpac-data.org/. Interactive data visualizations are provided through a website (https://data-viz.motrpac-data.org) and HTML reports summarizing the multi-omic graphical analysis results in each tissue. Processed data and analysis results are additionally available in the MotrpacRatTraining6moData R package (https://github.com/MoTrPAC/MotrpacRatTraining6moData).

Raw and processed data for each ome were also deposited in the appropriate public repositories as follows. RNA-Seq, ATAC-seq, and RRBS: SRA (PRJNA908279) and GEO (GSE242358); multiplexed immunoassays: IMMPORT (SDY2193); metabolomics: Metabolomics Workbench (Project ID PR001020); proteomics: MassIVE (MSV000092911, MSV000092925, MSV000092922, MSV000092924, MSV000092923, MSV000092931).

We used the following external datasets: release 96 of the Ensembl Rattus norvegicus (rn6) genome (https://ftp.ensembl.org/pub/release-96/fasta/rattus_norvegicus/dna/) and gene annotation (https://ftp.ensembl.org/pub/release-96/gtf/rattus_norvegicus/Rattus_norvegicus.Rnor_6.0.96.gtf.gz); RefSeq protein database (https://ftp.ncbi.nlm.nih.gov/refseq/R_norvegicus/, downloaded 11/2018); NCBI's "gene2refseq" mapping files (https://ftp.ncbi.nlm.nih.gov/gene/DATA/gene2refseq.gz, accessed 12/18/2020); RGD rat gene annotation (https://download.rgd.mcw.edu/data_release/RAT/GENES_RAT.txt, accessed 11/12/2021); BioGRID v4.2.193 (https://downloads.thebiogrid.org/File/BioGRID/Release-Archive/BIOGRID-4.2.193/BIOGRID-ORGANISM-4.2.193.tab3.zip); STRING v11.5 (https://stringdb-downloads.org/download/protein.physical.links.v11.5/10116.protein.physical.links.v11.5.txt.gz); GENCODE release 39 metadata and annotation files (https://ftp.ebi.ac.uk/pub/databases/gencode/Gencode_human/release_39/, accessed 1/20/2022); MatrisomeDB (https://doi.org/10.1093/nar/gkac1009); MitoPathways database available through MitoCarta (https://personal.broadinstitute.org/scalvo/MitoCarta3.0/); PTMSigDB v1.9.0 PTM set database (https://doi.org/10.1074/mcp.TIR118.000943); UniProt human proteome FASTA for canonical protein sequences (UniProtKB query "reviewed:true AND proteome:up000005640", download date 02/03/2021); CIBERSORT's LM22 leukocyte gene signature matrix (https://doi.org/10.1007%2F978-1-4939-7493-1_12); published results from Amar et al. (https://doi.org/10.1038/s41467-021-23579-x), Bye et al. (https://doi.org/10.1152/physiolgenomics.90282.2008), and Hostrup et al. (https://doi.org/10.7554/elife.69802); GTEx v8 gene expression data (dbGaP Accession phs000424.v8.p2). See details in the Methods (Supplementary Information).

Human research participants

Policy information about studies involving human research participants and Sex and Gender in Research.

Reporting on sex and gender	N/A - no human participants
Population characteristics	N/A - no human participants
Recruitment	N/A - no human participants
Ethics oversight	N/A - no human participants

Note that full information on the approval of the study protocol must also be provided in the manuscript.

Field-specific reporting

Please select the one below that is the best fit for your research. If you are not sure, read the appropriate sections before making your selection.

☒ Life sciences ☐ Behavioural & social sciences ☐ Ecological, evolutionary & environmental sciences

For a reference copy of the document with all sections, see nature.com/documents/nr-reporting-summary-flat.pdf

Life sciences study design

All studies must disclose on these points even when the disclosure is negative.

Sample size	Sample sizes were dictated by a combination of resource limitations and assay-specific expertise given that biological replicates were from an inbred strain.
Data exclusions	98 (1.0%) of 9466 samples were identified as outliers and excluded from downstream analysis. For metabolomics datasets, we calculated each sample's median correlation value against the other N-1 samples and selected a threshold to designate outliers as those with below-threshold median correlation values. For immunoassay data, measurements for analytes with fewer than 20 beads in a well were removed due to lack of accuracy; samples with more than 50% missing values were removed due to high missingness; features with at least two missing values for a single experimental group (e.g., males trained for 2 weeks) were removed due to lack of power. For all proteomics, transcriptomics, RRBS, and ATAC-seq datasets, we examined the top three principal components of each tissue separately. Samples were flagged if they fell outside of three times the interquartile range for at least one of the first three principal components. All identified outliers were manually inspected before removal from the final dataset used for downstream analysis. Specific reasons for excluding each sample are provided in Supplementary Table 1.
Replication	3-6 biological replicates were analyzed per sex/tissue/time point combination. Additionally, we found moderate agreement between our results and comparable existing mouse and human studies (Amar et al., Bye et al., Hostrup et al.). Given the scale of the animal experiment and resulting dataset, it would have been prohibitively expensive to replicate the study.
Randomization	Following the initial acclimation period, rats went through a 12-day treadmill familiarization protocol to expose the rats to the treadmill and to identify potential non-compliant rats. Those rats that were unable to run on the treadmill for 5 minutes at a speed of 10 m/min and grade of 0° were classified as non-compliant and removed from the study. Rats that successfully completed the 12-day familiarization protocol were entered in the rat database and randomized into a control or training group so that mean body weight of the groups were equal. The 8-week rats were randomly assigned to control or training within sex and tertile of weight. 4-week rats were assigned to control without randomization. 1- and 2- week rats were randomly assigned to 1- or 2-week training within sex and tertile of weight.
Blinding	Each Chemical Analysis Site had a single Batching Officer with access to the unblinded phenotypic data, which was necessary to determine batches of samples that were well-balanced in terms of sex, intervention group, and time point. Otherwise, investigators were blinded to the experimental group during sample collection and sample processing. As this is a discovery study, investigators were not blinded to phenotypic data for computational analyses.

Reporting for specific materials, systems and methods

We require information from authors about some types of materials, experimental systems and methods used in many studies. Here, indicate whether each material, system or method listed is relevant to your study. If you are not sure if a list item applies to your research, read the appropriate section before selecting a response.

Materials & experimental systems

n/a	Involved in the study	
☐	☒	Antibodies
☒	☐	Eukaryotic cell lines
☒	☐	Palaeontology and archaeology
☐	☒	Animals and other organisms
☒	☐	Clinical data
☒	☐	Dual use research of concern

Methods

n/a	Involved in the study	
☒	☐	ChIP-seq
☒	☐	Flow cytometry
☒	☐	MRI-based neuroimaging

Antibodies

Antibodies used	Levels of 54 cytokines and hormones were measured in rat samples using five Luminex® panels: MILLIPLEX MAP Rat Cytokine/ Chemokine Magnetic Bead Panel (Millipore, RECYTMAG-65K); MILLIPLEX MAP Rat Myokine Magnetic Bead Panel (Millipore, RMYOMAG-88K); MILLIPLEX MAP Rat Metabolic Hormone Magnetic Bead Panel (Millipore, RMHMAG-84K); MILLIPLEX MAP Rat Putuitary Magnetic Bead Panel (Millipore, RPTMAG-86K); MILLIPLEX MAP Rat Adipokine Magnetic Bead Panel (Millipore, RADPKMAG-80K). Luminex® Magnetic Beads are antibody-conjugated beads in solution (capture or primary antibody), with premixed formats available for select kits.
Validation	Custom Assay CHEX control beads (Radix BioSolutions, Georgetown, Texas) were added to all wells to monitor instrument performance, application of the detection antibody, application of the fluorescent reporter, and nonspecific binding (CHEX1, CHEX2, CHEX3, and CHEX4, respectively) (Montoya et al., 2017).

Animals and other research organisms

Policy information about studies involving animals; ARRIVE guidelines recommended for reporting animal research, and Sex and Gender in Research

Laboratory animals	Adult male and female Fischer 344 (F344) inbred rats were obtained from the National Institute on Aging (NIA) rodent colony. All animals were 6 months old at the beginning of the intervention.
Wild animals	The study did not involve wild animals.
Reporting on sex	Equal numbers of male and female animals were included in the study. Sex-biased results are described extensively.
Field-collected samples	The study did not involve samples collected from the field.
Ethics oversight	All animal procedures were approved by the Institutional Animal Care and Use Committee at the University of Iowa.

Note that full information on the approval of the study protocol must also be provided in the manuscript.
